# What research evidence is required on violence against women to inform future pandemic preparedness? A scoping review of the research evidence and gaps

**DOI:** 10.1136/bmjgh-2024-015877

**Published:** 2024-12-11

**Authors:** Aoibheann Brennan-Wilson, Qurat Ul Ain, Canan Ozkaya, Avni Amin, Claudia García-Moreno, Allen Thurston, Alison Mackenzie, Susan Lagdon, Patrick Stark, Maria Lohan

**Affiliations:** 1 School of Nursing & Midwifery, Queen's University Belfast, Belfast, UK; 2 School of Social Sciences, Education, and Social Work, Queen's University Belfast, Belfast, UK; 3 Department of Sexual and Reproductive Health, World Health Organization, Geneva, Switzerland; 4 School of Psychology, Ulster University, Coleraine, UK; 5 Hitotsubashi Institute for Advanced Study, Hitotsubashi University, Kunitachi, Tokyo, Japan

**Keywords:** Review, COVID-19, Public Health

## Abstract

**Introduction:**

Violence against women (VAW) during the COVID-19 pandemic was described as the ‘shadow pandemic’ with an increase in reports of VAW. As countries now focus on becoming more resilient to future pandemics, it is critical to understand what we learnt about evidence on VAW burden, prevention, and response during the COVID-19 pandemic. The WHO commissioned this scoping review to gain an understanding of the research evidence on VAW during COVID-19 and to inform future pandemic preparedness efforts.

**Methods:**

Terms relating to VAW and COVID-19 were used to search six databases between 1 January 2020 and 11 February 2023, inclusive of all study designs. Data on thematic focus (ie, burden of violence and/or interventions/services), types of violence, study design, study setting and participant characteristics were synthesised.

**Results:**

Of 25 080 identified records, 694 publications were reviewed in full text and 419 publications were included. The majority (>95%) of the published research was devoted to documenting the burden of VAW, while only 6.2% studied solutions (interventions/services), with even less emphasis on identifying how to prevent VAW in a pandemic context (1%). Gaps in research on VAW that existed prior to the pandemic on women who face multiple intersecting forms of disadvantage endured. Outstanding also was the gap in research on digital-mediated violence (<5%), even as reports of online facilitated violence soared. Additionally, gaps in evidence on specific types of violence such as femicide, forced marriage and acid attacks persisted.

**Conclusion:**

That VAW will be a critical concern, and its impacts worsened during pandemics in the future is certain. The research community does not need to spend more on understanding the burden of forms of VAW but pivot to research to adapt and innovate how to deliver prevention and support services, especially to populations who are disproportionately impacted. In addition to addressing this broader gap in prevention and response during pandemics, further evidence is required on the specific area of technology-mediated violence, femicide, forced marriage and acid attacks.

**WHAT IS ALREADY KNOWN ON THIS TOPIC?**

**WHAT THIS STUDY ADDS**

- This is the first scoping review to provide an overview of the available published evidence on all forms of VAW during the COVID-19 pandemic, and to identify gaps in this evidence for future pandemic preparedness.
- The review identified that the majority (>95%) of the published research was devoted to describing, measuring, and documenting the burden of VAW.
- The review identified a significant gap in that only 6.2% of published research studied solutions (interventions/services), with even less emphasis on identifying how to prevent VAW in a pandemic context (1% of evidence).

**HOW THIS STUDY MIGHT AFFECT RESEARCH, PRACTICE OR POLICY**

- This review makes clear that for future pandemic preparedness, we need to focus our research attention on solutions to VAW, including services, prevention interventions, and policy responses.
- Solutions to VAW also need to be informed by a deeper understanding of less researched types of violence and lesser-reached groups of women.

## Introduction

Violence against women (VAW) is defined by the United Nations as ‘any act of gender-based violence that results in, or is likely to result in, physical, sexual or psychological harm or suffering to women, including threats of such acts, coercion or arbitrary deprivation of liberty, whether occurring in public or in private life.’^1^ The WHO estimates that almost one in three women have been subjected to physical and/or sexual violence by a current or former male intimate partner or sexual violence by someone who is not a current or former intimate partner at least once in their lifetime.^2^ The health burden of such violence is also considerable as established by data on the physical and mental health consequences.^3^ It is also estimated that pandemics may exacerbate vulnerability to violence and the associated health consequences. For example, widespread reports during the Ebola and Zika viruses highlighted significant increases in physical and sexual violence towards women.^4^ A report by the Global Development Agency^4^ suggests that this is because pandemics create environments which may exacerbate or trigger various forms of VAW. More recently, the United Nations Agency for Gender Equality and Women’s Empowerment (UN Women)^5^ described the increase in reported VAW during COVID-19 as the ‘shadow pandemic’. The reports were disturbing yet predictable, as measures employed to limit the spread of the virus (ie, lockdowns/stay-at-home orders and physical distancing) confined women to ‘lock down’ with their abusers.^6^ Additionally, addressing the pandemic led to funding cuts and operational changes that reduced service access and the availability of social support for survivors of VAW.^5^

Between 2020 and 2023, there was an increase in studies examining the impact of COVID-19 on the burden of VAW, service availability and efforts to prevent and respond to such violence. There was also an increase in systematic reviews of this evidence. Most systematic reviews published to date focus on the burden of VAW during the pandemic.^7^,^16^ Only one review synthesised evidence of intervention studies,^17^ but this review included intervention studies conducted before the COVID-19 pandemic and focused on how these interventions could be adapted and delivered in the context of COVID-19 physical distancing restrictions, such as technology-facilitated delivery. The majority of existing reviews also focused specifically on intimate partner violence (IPV)^7 9 12 13^ or domestic violence (DV).^8 10 14 17 18^ The WHO defines IPV as behaviour that ‘causes physical, sexual or psychological harm, including acts of physical aggression, sexual coercion, psychological abuse and controlling behaviours’.^19^ DV is defined by the UN^20^ as any pattern of behaviour that is used to ‘gain or maintain power and control over an intimate partner’ that results in physical, emotional, financial or psychological harm or pain. Only one review synthesised evidence on VAW more broadly and this was published early during the pandemic.^11^ The majority of ongoing systematic reviews on VAW and COVID-19 that are registered with PROSPERO continue this focus on IPV or DV in the context of the COVID-19 pandemic, although among specific groups, such as ‘urban poor’,^21^ or specific populations, such as Latin American women,^22^ or specific geographical areas including Africa,^23^ Australia,^24^ Brazil^25^ and Southern Asia.^26^

The scope of the current review is broader than the currently available reviews and those underway as it pertains to all types of VAW, including but not limited to those that describe perpetrator relationships such as IPV (ie, former or current partner) and DV (ie, perpetrator residing in the same household). This includes, for example, femicide, forced marriage and acid attacks. In line with recent efforts to better understand the scale and scope of technology-facilitated gender-based violence, our review also summarises the available evidence on face-to-face and technology-mediated violence during COVID-19.^27^ The WHO commissioned this scoping review to better understand the scope of the evidence generated during the COVID-19 pandemic on VAW burden, prevention and response. The purpose of this review is to inform the evidence, programmes, services and policies that are required on VAW for future pandemic preparedness and in meeting the sustainable development goals (SDGs) on preventing and responding to VAW.^27^ The specific research questions are as follows:

1. What are the thematic topics or foci that have been addressed in the literature on VAW during the COVID-19 pandemic?
2. What are the types of research designs used?
3. What are the main gaps in this research on VAW during COVID-19 to inform future pandemic preparedness?

## Methods

This review employs a scoping review method as scoping reviews have been identified as the most appropriate method for providing an overview of the available research evidence.^28^ This method was also selected because our initial searches indicated a large number of studies^29^ and scoping reviews facilitate a rapid synthesis of the available research evidence through a systematic search of the literature^30^ while also offering more timely completion to inform decision-making.^31^,^33^ A scoping review is ideally followed by in-depth focused systematic reviews of areas of research identified in a scoping review.^34^,^36^ The results of this scoping review are reported according to the Preferred Reporting Items for Systematic Reviews and Meta-Analyses extension for Scoping Reviews (PRISMA-ScR) reporting guidelines.^37^ The completed PRISMA-ScR checklist is provided in Supplemental File, online supplemental appendix A. An assessment of the methodological quality of included studies is not recommended for scoping reviews^28^ and is not, therefore, included in the current review. The full methods are described in the prepublished protocol.^29^

### Search strategy

A prepublished search strategy was developed in consultation with WHO (AA) and combined search strings using Boolean operator AND terms relating to violence AND women AND COVID-19.^29^ This search strategy was used to systematically search six electronic databases: MEDLINE, PsycINFO, Social Science Citation Index–expanded, Cochrane Library (including CENTRAL), Campbell Systematic Reviews Journal and Scopus. Studies published between 1 January 2020 and 11 February 2023 were included. The start date coincides with the earliest possible mention of the COVID-19 pandemic as a novel respiratory infection. The end date was close to the final end date of the pandemic, which was declared to be 5 May 2023, by the WHO.^38^ The full search strategy is provided in Supplemental File, online supplemental appendix B.

### Study selection

This review considered all evidence relating to VAW, including the prevalence and experience of VAW and types of violence. We also considered interventions that involved providing services to survivors of VAW in the context of the COVID-19 pandemic, as well as any interventions or services aimed at reducing VAW, including interventions with perpetrators of VAW.

To manage an anticipated large volume of studies, searches were limited to English language peer-reviewed publications only. All quantitative study designs were included. Qualitative studies with 18 or more participants or interviews were also included. This minimum sample size was selected as we sought to include qualitative studies with a reasonably good number of study participants and to maximise the potential for data saturation within diverse types of qualitative studies.^29 39^ We included studies involving women aged 15 years or older to align with the age range on which the WHO estimates of VAW are based. Studies involving men were included where the focus was on interventions or services provided to women and men, or studies involving men as perpetrators of VAW.

Records identified from the search of each database were imported into EPPI Reviewer (V.4.14.1.0). After removing duplicates, remaining records were screened for inclusion by title and abstract. To ensure quality control, the first 100 records, chosen at random, were independently dual-screened. Kappa was calculated to assess inter-rater reliability using the recommended method from the Cochrane Handbook.^40^ Analysis of the first 10 articles screened for inclusion showed very good inter-rater reliability between three reviewers (CO, QUA and AB-W). There were no disagreements between reviewer 1 and reviewer 2 (kappa=1), between reviewer 1 and reviewer 3 (kappa=1) or between reviewer 3 and reviewer 2 (kappa=1). We attribute this to the extensive preparation undertaken, which developed shared understanding between the reviewers prior to the commencement of screening. Agreements and disagreements on further screening were discussed by the two reviewers assigned to the task and any disagreements were discussed with a third member of the team (AT, AM or ML). The full text of potentially relevant records was retrieved and screened to determine if they met the inclusion criteria.

For quality control purposes, dual data extraction was conducted on the first 30 records (AB-W, CO and QUA) and the team discussed any differences in data extraction with ML. Following this, data extraction was conducted by three coauthors (CO, QUA and AB-W). The full list of data extraction codes is provided in Supplemental File, online supplemental appendix C. The extracted data has been summarised and analysed using narrative synthesis methods.^41^

### Patient and public involvement

Patients were not involved in the design, or conduct, or reporting of this study.

## Results

**Figure 1:**
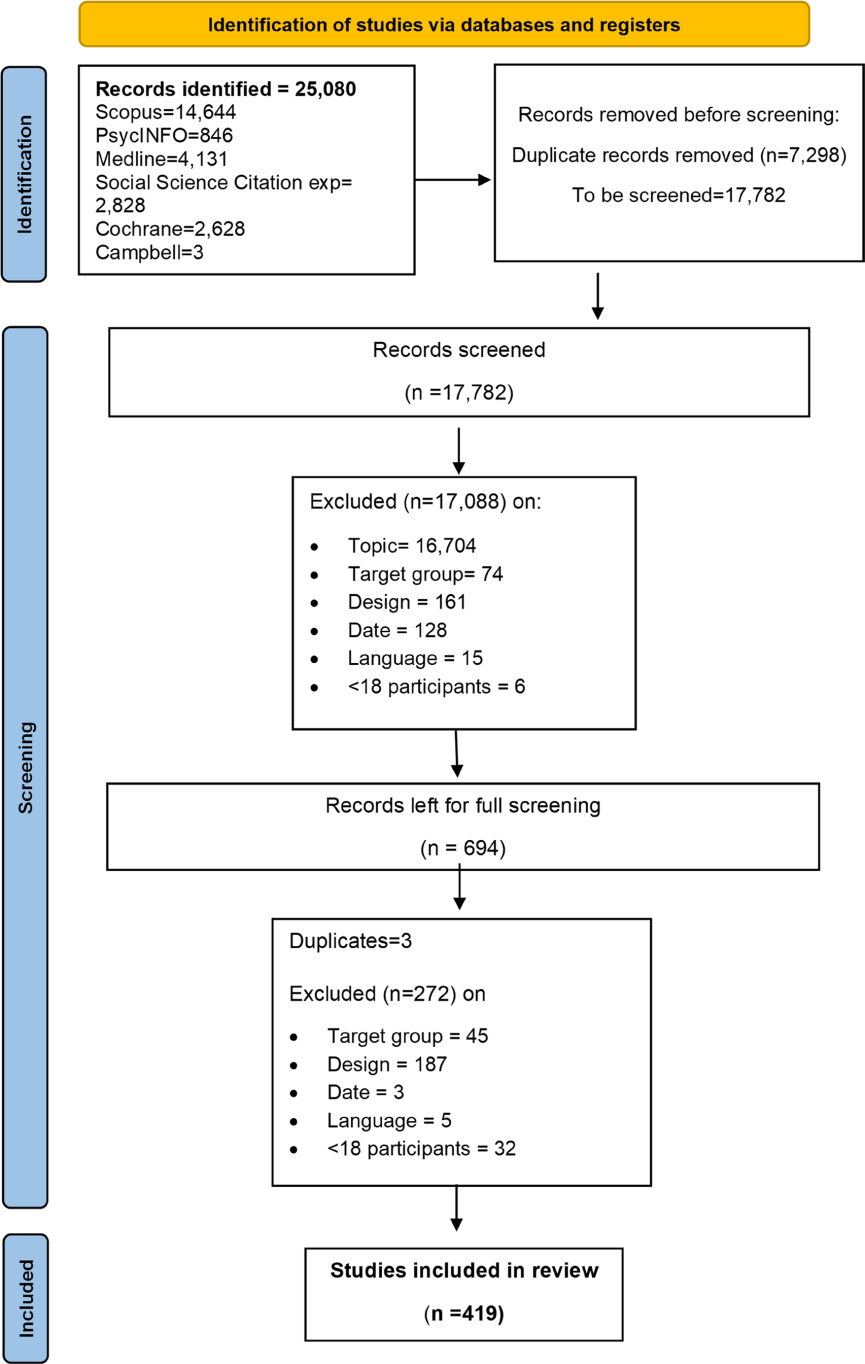
PRISMA flow diagram showing identification, screening, exclusion and selection of studies. Adapted from Page *et al*^66^. PRISMA, Preferred Reporting Items for Systematic Reviews and Meta-Analyses.

A total of 25 080 records were identified for the current review (see figure 1). After removing duplicates, a total of 17 782 records were screened on title and abstract. A further 17 088 records were excluded at this stage and 694 records were retrieved for full-text screening. A total of 419 studies were included in the final review. No studies were retracted after data extraction was completed. A summary of study characteristics of the 419 studies can be found in Supplemental File, online supplemental appendix D. Here, we describe the study characteristics in relation to the predefined review questions below. Percentages were calculated using the number of included studies (n=419) as the denominator.

### What thematic topics were addressed?

Most studies identified in this review addressed ‘burden of violence’ (n=401; 95.7%). Only a small number of studies focused on the thematic category of ‘interventions/services to address violence’ (n=26; 6.2%) and nine (2.2%) studies addressed both thematic categories. We allowed for multiple thematic focus of studies and hence the proportions add to more than 100%. Within the 6.2% of studies that addressed interventions/services, the dominant focus was on services or interventions delivered in response to violence, while only four of these studies included a focus on services or interventions to prevent violence. Only one study (0.2%) focused on ‘interventions/services with perpetrators to address violence’.^42^ Of the 95.7% of studies that addressed the burden of violence, a total of 237 studies reported incidents of violence and a total of 184 reported the impact of violence through standard measures and/or women’s own reported experience of violence.

#### What types of violence were studied?

**Figure 2:**
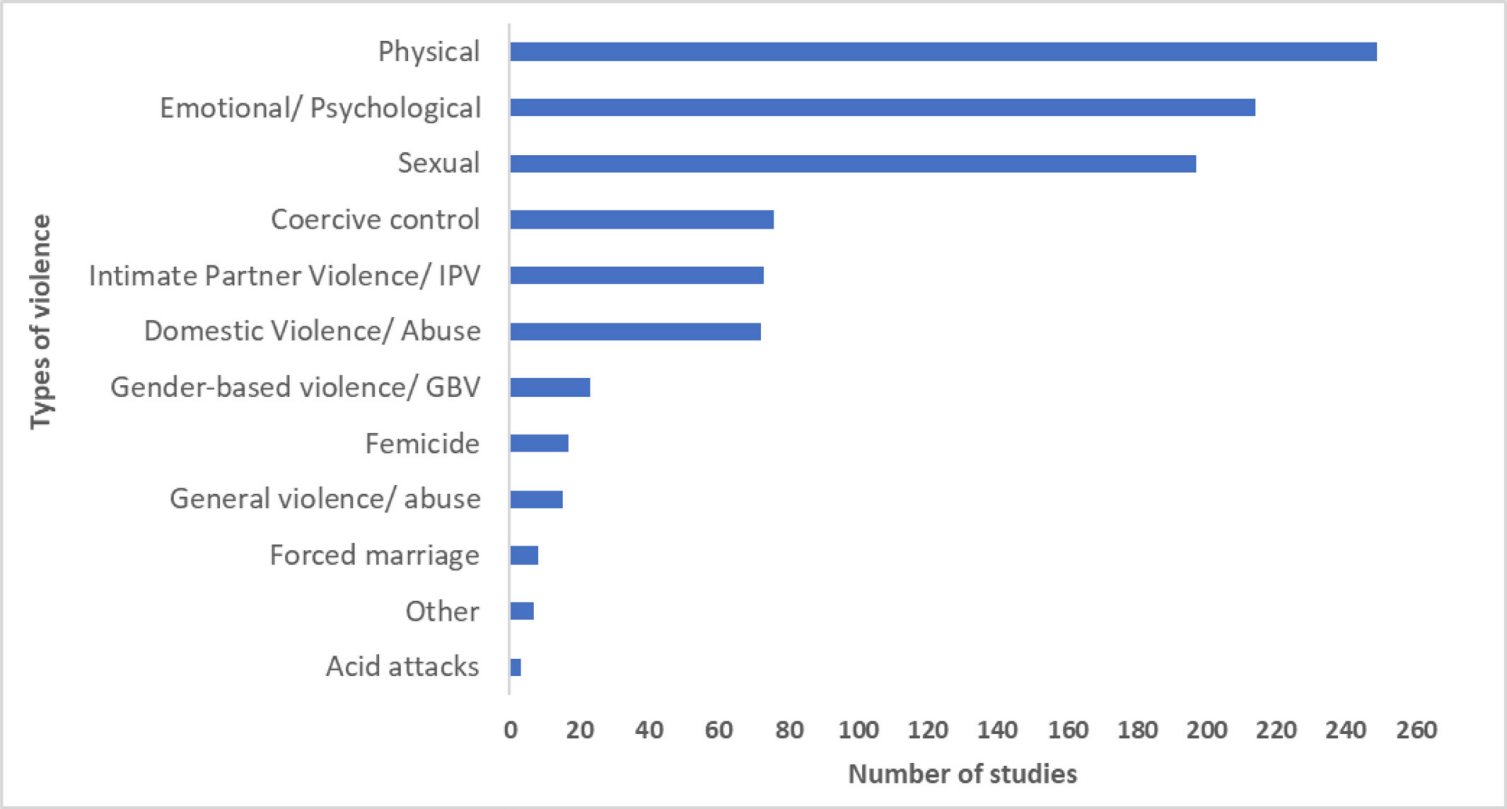
Types of violence addressed by studies.

While studies usually addressed multiple forms of violence, the most common type of violence addressed by studies was physical violence (n=250; 59.7%), followed by emotional/psychological violence (n=212; 50.6%), and then sexual violence (n=198; 47.3%). There was an an additional 76 (18.1%) specifically on ‘coercive control’. Coercive control is used to describe acts or patterns of behaviour used to harm, punish or frighten. Fewer studies addressed femicide (n=17; 4.1%), forced marriage (n=8; 1.9%) and acid attacks (n=3; 0.7%). Many studies also described perpetrator relationships such as DV (ie, by a perpetrator residing in the same household) (n=72; 17.2%) and IPV (ie, current or former partner) (n=73; 17.4%). Finally, a few studies referred to categories such as gender-based violence (n=23; 5.5%), or just ‘violence/abuse’ without disaggregating into types and relationship with perpetrators (n=15; 3.6%). Figure 2 shows the number of studies on different types of violence (see online supplemental appendix E for more information on these studies).

In relation to modality of violence, most studies (n=378; 90.2%) addressed in-person experiences/perpetration of violence. A small number (n=19; 4.5%) addressed online mediated experiences/perpetration of violence. Just over half of these online studies (n=10) addressed online mediated violence exclusively (2.4% of included studies), while the other nine addressed both online and in-person violence. A small number of studies did not specify the modality of violence (n=34; 8.1%).

### What research designs were used?

Quantitative research designs were the most common (n=320; 76.4%) compared with qualitative (n=69; 16.5%) and mixed-methods (n=30; 7.2%) research designs. This breakdown of research designs was largely consistent across studies in lower-income and middle-income countries (LMICs) and high-income countries (HICs). Most studies collected primary data (n=318; 75.9%) compared with using secondary data (n=107; 25.5%) and used virtual data collection methods (n=264 online (63.0%) and n=64 telephone (15.3%)). See Supplemental File, online supplemental appendix F for a more detailed analysis of research designs.

As noted under the thematic breakdown of studies above, 401 studies (95.7%) focused on examining the burden of violence, and approximately half of these (n=203) used quantitative methods. The dominant aim of these quantitative studies was to measure incidents or reports of violence using self-report surveys (n=136/203 studies). Given the dominance of research examining the incidence of violence, we examined these studies (n=136) closer in terms of methods of measurement. We found a small number of these studies (17 out of 136) used the full or an adapted version of the WHO multicountry study on women’s health and DV survey. Further, only 12 of these 136 studies used the WHO survey instrument and additionally reported methods (data collection, safety, signposting) that align with the WHO guidelines on ethical measurement of VAW (see Supplemental File, online supplemental appendix G). The remaining studies that used self-report surveys to measure incidents of VAW used either bespoke measures (n=66 out of 136) or standardised measures (n=53 out of 136) such as the Revised Conflicts Tactics Scale.

We also examined the use of representative samples within this body of 136 studies that used surveys to examine reports of VAW. A significant proportion (n=40) involved a representative sample. However, of these, only four studies used a representative sample in combination with the WHO questions and guidelines for ethical research on VAW. Overall, of the 40 studies using representative samples, these were somewhat more likely to be conducted in LMICs (n=23/40 studies) compared with HICs (n=16/40). Studies of reports of violence using non-representative samples tended to be on specific groups of populations, such as pregnant and postpartum women or adolescent women. See below for a further discussion of target groups and Supplemental File, online supplemental appendix H for a further analysis of studies examining VAW by thematic focus, country income context and research design.

### What are the main gaps in the research evidence?

Already our findings have highlighted gaps in the evidence in relation to the thematic focus of studies, types of violence studied and research methods. We also examined for additional gaps in relation to target groups, participant characteristics, research settings and geographical regions.

**Figure 3:**
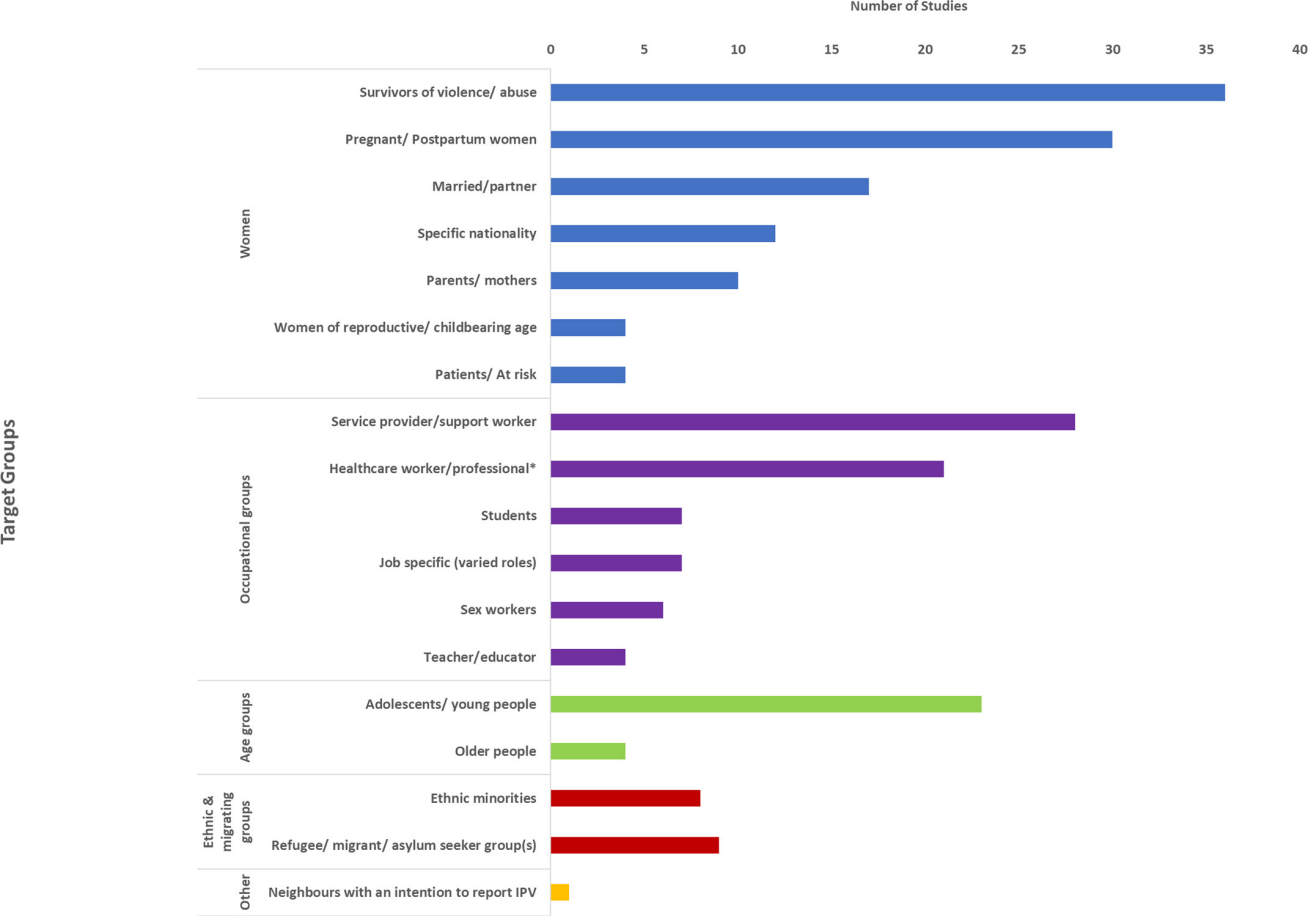
Number of studies within target group categories. IPV, intimate partner violence.*We distinguish between studies where the target group is ‘healthcare workers/professionals’ who have experienced violence, and studies where the target group is ‘service providers/support workers’ who work with survivors.

Some studies recruited or targeted specific groups of women or professions based on the target groups’ increased vulnerability to COVID and/or violence, their experience of violence, or to provide context-specific evidence of VAW during COVID-19. Target populations included but were not limited to, survivors of VAW (n=36; 8.6%), pregnant/postpartum women (n=30; 7.2%), healthcare professionals (n=21; 5.0%), service providers/support workers (n=28; 6.7%), adolescents/young people (n=23; 5.5%) and specific nationalities in geographically defined areas (n=12; 2.9%) (figure 3). Notable gaps lay in relation to groups of women who may experience multiple or intersecting forms of disadvantage/adversities, including asylum seekers/refugees (n=12; 2.9%), ethnically diverse groups (n=8; 1.9%) and sex workers (n=4; 1.0%) (see Supplemental File, online supplemental appendix I). Examples of studies where intersectional disadvantage was studied included studies in the USA on low-income Hispanic, African American and Asian participants, as well as sexual minority participants^43^,^46^ and studies conducted in Uganda, Nigeria and Mozambique addressing sex workers, women at risk of HIV and young refugees.^47^,^49^

**Figure 4:**
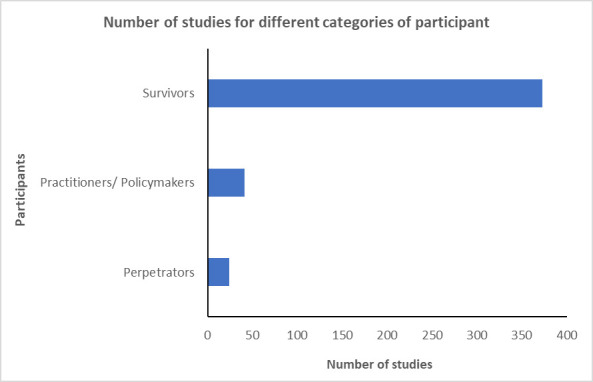
Number of studies for different categories of participants.

As shown in figure 4, most study participant groups included survivors of VAW (n=373; 89.0%), leaving an evidence gap in research with practitioners (n=41; 9.8%) and perpetrators of violence (n=24; 5.7%) which aligns with our earlier reported finding on the limited research evidence on services and interventions to address VAW. Our findings also highlight some important gaps in relation to sexual and gender-diverse groups in included studies. For example, of the studies that reported the gender of participants (n=395; 94.3%), most involved women (n=383), and many also included men as participants (n=182) (seeonline supplemental appendix J). However, the results suggest that there are few studies that included participants with non-binary gender identities, including transgender women (n=13; 3.1%), non-binary or gender fluid individuals (n=14; 3.3%) and ‘other’ gender identities (n=31; 7.4%) who experienced violence during the COVID-19 pandemic. It is worth noting that our search strategy which focused on terms relating to ‘women’ may not have captured studies that were focused more specifically on other gender identities. See Supplemental File, online supplemental appendix J for an additional analysis of the terminology used in the included studies in reporting sex or gender identity.

The results also highlight a gap in the evidence in relation to the reporting of the sexual orientation of women who experienced violence during the COVID-19 pandemic. Only a small number of studies (n=35; 8.3%) reported the sexual orientation of the participants. Most of these studies that reported the sexual orientation included participants who identified as heterosexual (n=34; 8.1%), but some of these studies also included participants who identified as lesbian (n=24; 5.7%), gay (n=19; 4.5%), bisexual (n=27; 6.4%), lesbian, gay, bisexual, transgender, queer, questioning, intersex, asexual (LGBTQI+; n=5; 1.2%) and ‘other’ sexual orientation (n=27; 6.4%) (see Supplemental File, online supplemental appendix J for a more detailed analysis).

Of those studies that did report a specific setting (n=195), the majority were conducted in healthcare settings (n=82), followed by community settings (n=59). This included urban (n=22 of 59 studies), rural (n=12) and a mixture of urban and rural community settings (n=25). A smaller number of studies were conducted in justice/policing settings (n=23 of 195 studies), followed by online (n=13), education (n=9) and domestic settings (n=9) (see Supplemental File, online supplemental appendix K).

**Figure 5:**
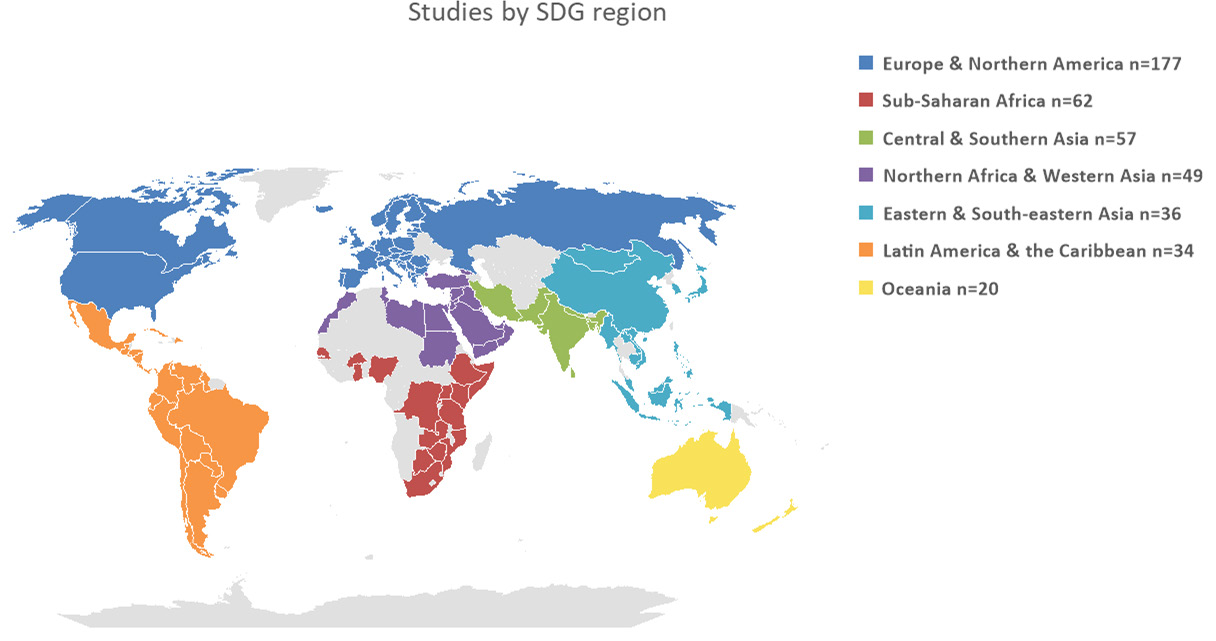
Studies by SDG Region. SDG, Sustainable Development Goal.

An analysis of the SDG regions (figure 5) reveals that most studies were conducted in the ‘Europe and Northern America’ region (n=177; 42.0%). Smaller numbers of studies were conducted in ‘sub-Saharan Africa’ (n=62; 14.7%), ‘Central and Southern Asia’ (n=57; 13.6%), ‘Northern Africa and Western Asia’ (n=49; 11.6%), ‘Eastern and South-Eastern Asia’ (n=36; 8.6%), ‘Latin America and the Caribbean’ (n=34; 8.1%) and Oceania (n=20; 4.8%) regions (see the Supplemental File, online supplemental appendix L). An additional analysis based on the WHO world regions is provided in (Supplemental File, online supplemental appendix M).

Similar numbers of studies were conducted in LMICs (n=207; 49.4%) and HICs (n=203; 48.5%), with only 10 across both income contexts (2.4%). A more nuanced picture emerges when we look at what the focus of the research was and where it was conducted. Within the burden of violence thematic category, similar numbers of studies were conducted in LMICs (n=200) and HICs (n=185), with nine across both income contexts. Within the intervention/service category, studies were more likely to be conducted in HICs (n=18) than LMICs (n=7) with only one conducted across both income contexts.

## Discussion

As countries now focus on becoming more resilient to future pandemics it is critical to understand what we learnt about the evidence on VAW data, prevention and response during the COVID-19 pandemic. To the best of our knowledge, this is the most comprehensive and up-to-date scoping review of the research conducted on VAW during the COVID-19 pandemic. Our systematic search for prior reviews of VAW in the context of the COVID-19 pandemic shows that the available systematic review evidence,^9^,^1317 18 50 51^ including registered protocols,^21^,^24^ only includes systematic reviews of DV and IPV during COVID-19 rather than VAW more broadly.

This scoping review contributes to the field of VAW in several ways. First, consistent with those of previous reviews that focus specifically on IPV and DV during COVID-19,^9 10 12 13 17 50^ our review identifies that the primary thematic focus in the broader VAW and COVID-19 research has been on the burden of violence (95.7%), leaving a relative research deficit on interventions and services addressing violence (6.2%), a key finding that needs addressing if VAW is to be reduced or prevented. Second, and of significance for future research, is that within this relatively small category of research on interventions/services during COVID-19, we identified that a very small proportion of the research had a specific focus on prevention in a pandemic context (1%).^16^ Overall, our review demonstrates that there is a deficit of evaluation research, using any research designs, of interventions and services on VAW during the COVID-19 pandemic to adequately inform future pandemic preparedness, especially in terms of prevention and response.

Moreover, we also identified gaps in research on types of VAW and did so by considering a wider range of VAW than in the available systematic reviews which focus on IPV and DV.^9 10 12 13 17 50^ Our review identifies notable gaps in relation to femicide, forced marriage and acid attacks (<7% of included studies in combination). Importantly, there was a gap in the evidence on online mediated violence and abuse (4.5% of included studies). The growing use of digital devices and social media was brought into sharp focus during the pandemic, and along with it, the rise in technology-facilitated VAW. This requires much more attention in terms of understanding the scale, impacts and ways to prevent it and the global symposium on technology-facilitated violence^27^ is one such welcome effort in the past year.

Our review is also more inclusive than other available reviews because it considers a large range of participant characteristics including study ‘target’ groups such as older women, pregnant and postpartum women, and adolescents. We identified a gap in research on gender-diverse populations (<5% of included studies), and studies that reported the sexual orientation of participants (<10%), as well as in relation to ethnically diverse groups (<2%), and refugees and migrating populations (2.1%), and, therefore, an overall gap in research on VAW and other intersectional forms of disadvantage. There were other notable gaps in relation to occupational target groups studied, such as the police (<1%) and sex workers (1.4%).

Our review is distinct in that it offers a global regional breakdown of available research on VAW during COVID-19, and in relation to a breakdown of research conducted, respectively, in LMICs and HICs. While we found a relatively even split in the proportion of studies across LMICs (51.3%) and HICs (49.6%), there was, however, especially little evaluation research of interventions/services in LMICs. Overall, the largest regional cluster of studies was conducted in the North America and Europe regions (54% combined), consistent with findings reported by one previous systematic review^9^ in relation to IPV and DV only.

Our results also highlight a larger number of quantitative studies with gaps in the evidence in relation to qualitative and mixed-methods studies (<25% of included studies). The lower proportion of qualitative research was likely owing to the difficulty of in-person research during COVID-19 and we also acknowledge here our exclusion criteria of qualitative studies with <18 participants, which resulted in 38 identified studies being excluded from this review. Nonetheless, while the review has demonstrated a disproportionate emphasis on measuring and understanding the burden of VAW, consistent with another review in the field,^52^ we observed that less than 10% of these studies used the WHO multicountry study on women’s health and DV survey instrument and the WHO ethical standards of conducting research to measure incidence of VAW, considered to be the most reliable and ethical way to measure VAW. In addition, only four studies used a representative sample in combination with the WHO questions and guidelines for ethical research on VAW to examine prevalence. Given a dearth of research using reliable and ethical measures of prevalence, and robust samples, it is challenging to establish the robustness of the widespread conclusion that prevalence of violence increased during the pandemic, largely because of methodological issues of measurement and sampling. Surveys did not use comparable measures or methods, and, at the moment, neither is there data from methodologically similar and high-quality surveys on prevalence of VAW conducted before and during the pandemic to analyse and arrive at conclusions.

Finally, we reflect on the nature of the new evidence collected on VAW during COVID-19 in comparison to systematic reviews of evidence on VAW prior to COVID-19.^53^,^64^ Prior to the pandemic, there was a greater focus in systematic reviews on intervention/service evaluation, especially with regard to treatment, while prevention interventions were still relatively less present in reviews.^54 61 62 64^ Prior to the pandemic, systematic reviews also tended to focus on IPV and DV or physical/sexual violence,^56^,^64^ neglecting other forms of violence and were more likely to include studies conducted in HICs. Our review incorporated a greater number of published studies in LMICs than in systematic reviews prior to the pandemic,^55 56^ suggesting a stronger investment in research in LMICs over time. The momentum of growing research in LMICs needs to be strengthened. In particular, intervention and service development research studies focused on codesign and evaluation of prevention actions, service responses and policy interventions are required to meet the SDGs on preventing and responding to VAW.^65^

### Limitations

As the literature search took place shortly before the COVID-19 pandemic was declared over by WHO, on 5 May 2023,^38^ research that is currently being completed and published on VAW during the pandemic will be missed by the scoping review and a further update may be warranted. Our search syntax, while comprehensive, excluded the use of controlled vocabulary (eg, MeSH terms in Medline). This is a limitation of our scoping review. The decision to focus on peer-reviewed research has been to improve the quality of evidence examined, but it means that grey literature on VAW during the pandemic has not been assessed. The exclusion of articles in languages other than English is another limitation of our scoping review.

### Conclusion

The research and VAW prevention and response field needs to balance its efforts and investments to focus more on how to adapt and innovate methods to prevent violence and deliver services. This is especially important with reference to populations who are disproportionately impacted because of intersecting vulnerabilities. The pandemic has brought into sharp focus the social and economic pressures faced by these groups during outbreaks and emergencies—conditions in which exposure to violence increases.

While the global health community is considering how to prepare for the inevitable future infectious diseases pandemics, it is imperative that it is prepared to prevent and respond to ‘shadow pandemics’ such as those related to VAW that are not inevitable and entirely preventable.

## Supplementary material

10.1136/bmjgh-2024-015877 online supplemental file 1

## Data Availability

All data analysis/data coding is made available in online supplemental appendices. All requests for access to additional data, including EPPI data extraction records, should be addressed to the corresponding author.

## References

[1] United Nations (1993). Declaration on the elimination of violence against women.

[2] WHO (2021). global, regional and national prevalence estimates for intimate partner violence against women and global and regional prevalence estimates for non-partner sexual violence against women. Executive summary.

[3] García-Moreno C, Abrahams N, Devries K (2013). Global and regional estimates of violence against women: prevalence and health effects of intimate partner violence and non-partner sexual violence.

[4] Peterman A, Potts A, O’Donnell M (2020). Framing the issue: pandemics and violence against women and children.

[5] United Nations Women (2021). Measuring the Shadow Pandemic. United Nations.

[6] Roesch E, Amin A, Gupta J (2020). Violence against women during covid-19 pandemic restrictions. BMJ.

[7] Alamolhoda S-H, Zare E, Doulabi MA (2023). Domestic Violence against Women and COVID-19 Quarantine: A Systematic Review Study. *CWHR*.

[8] Eapen DJ, Tsusaki RB, Mullassery DG (2023). A Systematic Review of Women’s Experiences of Interpersonal Violence During the COVID-19 Pandemic. Nurs Womens Health.

[9] Kourti A, Stavridou A, Panagouli E (2023). Domestic Violence During the COVID-19 Pandemic: A Systematic Review. Trauma Violence Abuse.

[10] Lausi G, Pizzo A, Cricenti C (2021). Intimate Partner Violence during the COVID-19 Pandemic: A Review of the Phenomenon from Victims’ and Help Professionals’ Perspectives. Int J Environ Res Public Health.

[11] Nasution LA, Fitriana LA (2020). Factors Affecting Violence Againts Women during the COVID-19 Pandemic: A Systematic Review. *JPKI*.

[12] Piquero AR, Jennings WG, Jemison E (2021). Domestic violence during the COVID-19 pandemic - Evidence from a systematic review and meta-analysis. J Crim Justice.

[13] Thiel F, Büechl VCS, Rehberg F (2022). Changes in Prevalence and Severity of Domestic Violence During the COVID-19 Pandemic: A Systematic Review. Front Psychiatry.

[14] Uzoho IC, Baptiste-Roberts K, Animasahun A (2023). The Impact of COVID-19 Pandemic on Intimate Partner Violence (IPV) Against Women. *Int J Soc Determinants Health Health Serv*.

[15] Sánchez OR, Vale DB, Rodrigues L (2020). Violence against women during the COVID-19 pandemic: An integrative review. Int J Gynaecol Obstet.

[16] Ndlovu S, Mulondo M, Tsoka-Gwegweni J (2022). COVID-19 impact on gender-based violence among women in South Africa during lockdown: a narrative review. Afr J Reprod Health.

[17] Weeks LE, Stilwell C, Rothfus M (2024). A Review of Intimate Partner Violence Interventions Relevant to Women During the COVID-19 Pandemic. Violence Against Women.

[18] Wake AD, Kandula UR (2022). The global prevalence and its associated factors toward domestic violence against women and children during COVID-19 pandemic-"The shadow pandemic": A review of cross-sectional studies. Womens Health (Lond).

[19] WHO Definition of intimate partner violence. https://apps.who.int/violence-info/intimate-partner-violence/.

[20] UN Definition of domestic violence. https://www.un.org/en/coronavirus/what-is-domestic-abuse.

[21] Negi S, Sahoo KC, Samantaray K (2022). Gender-based violence among urban poor during covid-19 pandemic in low- and middle-income countries: a systematic review. https://www.crd.york.ac.uk/prospero/display_record.php?ID=CRD42022378338.

[22] Gonzalez Alvarez MA, Pineda Roberto MA, Buitrago López CM (2021). Domestic violence against latin american women - frequency and trends in the covid-19 pandemic context: a systematic review. https://www.crd.york.ac.uk/prospero/display_record.php?ID=CRD42021272160.

[23] Tadesse DB, Gebrewahd GT, Gerensea H (2020). Intimate partner violence against women during covid-19 pandemic in africa: a systematic review and meta-analysis. https://www.crd.york.ac.uk/prospero/display_record.php?ID=CRD42020212631.

[24] Addo I, Asare B, Mensah E (2020). A systematic review of covid-19 pandemic and associated domestic violence in australia. https://www.crd.york.ac.uk/prospero/display_record.php?ID=CRD42020222069.

[25] Carvalho J, Gatinho G, Mourão A (2023). Domestic violence against women during the covid-19 pandemic in brazil: a systematic literature review.

[26] Mahmud S, Mohsin M, Ferdous F (2022). Measuring prevalence and monitoring the risk factors of violence against woman in southern asia pre- and during covid-19 pandemic: a systematic review and meta-analysis.

[27] UNFPA (2023). 2022 Global symposium on technology-facilitated gender-based violence results: building a common pathway.

[28] Peters MDJ, Marnie C, Tricco AC (2020). Updated methodological guidance for the conduct of scoping reviews. *JBI Evid Synth*.

[29] Ain QU, Ozkaya C, Amin A (2023). Violence against women during the Covid-19 Pandemic: Scoping review of the literature in collaboration with the World Health Organization protocol. Int J Educ Res Open.

[30] Tricco AC, Antony J, Zarin W (2015). A scoping review of rapid review methods. BMC Med.

[31] Plüddemann A, Aronson JK, Onakpoya I (2018). Redefining rapid reviews: a flexible framework for restricted systematic reviews. *BMJ EBM*.

[32] Robinson M, Aventin Á, Hanratty J (2021). Nothing so practical as theory: a rapid review of the use of behaviour change theory in family planning interventions involving men and boys. Reprod Health.

[33] Campbell F, Tricco AC, Munn Z (2023). Mapping reviews, scoping reviews, and evidence and gap maps (EGMs): the same but different— the “Big Picture” review family. Syst Rev.

[34] Ruane-McAteer E, Gillespie K, Amin A (2020). Gender-transformative programming with men and boys to improve sexual and reproductive health and rights: a systematic review of intervention studies. BMJ Glob Health.

[35] Ruane-McAteer E, Amin A, Hanratty J (2019). Interventions addressing men, masculinities and gender equality in sexual and reproductive health and rights: an evidence and gap map and systematic review of reviews. BMJ Glob Health.

[36] Ruane-McAteer E, Hanratty J, Lynn F (2018). Protocol for a systematic review: Interventions addressing men, masculinities and gender equality in sexual and reproductive health: An evidence and gap map and systematic review of reviews. *Campbell Syst Rev*.

[37] Tricco AC, Lillie E, Zarin W (2018). PRISMA Extension for Scoping Reviews (PRISMA-ScR): Checklist and Explanation. Ann Intern Med.

[38] WHO (2023). WHO chief declares end to covid-19 as a global health emergency. https://news.un.org/en/story/2023/05/1136367.

[39] Hennink MM, Kaiser BN, Marconi VC (2017). Code Saturation Versus Meaning Saturation. Qual Health Res.

[40] Higgins JPT, Green S (2011). Cochrane Handbook for Systematic Reviews of Interventions.

[41] Snilstveit B, Oliver S, Vojtkova M (2012). Narrative approaches to systematic review and synthesis of evidence for international development policy and practice. J Dev Eff.

[42] Cortis N, Smyth C, Valentine K (2021). Adapting Service Delivery during COVID-19: Experiences of Domestic Violence Practitioners. Br J Soc Work.

[43] Eaton AA, Ramjee D, Saunders JF (2023). The Relationship between Sextortion during COVID-19 and Pre-pandemic Intimate Partner Violence: A Large Study of Victimization among Diverse U.S Men and Women. *Victims & Offenders*.

[44] Riggle EDB, Drabble LA, Bochicchio LA (2021). Experiences of the COVID-19 Pandemic Among African American, Latinx, and White Sexual Minority Women: A Descriptive Phenomenological Study. Psychol Sex Orientat Gend Divers.

[45] Diaz A, Nucci-Sack A, Colon R (2022). Impact of COVID-19 Mitigation Measures on Inner-City Female Youth in New York City. J Adolesc Health.

[46] Peitzmeier SM, Fedina L, Ashwell L (2022). Increases in Intimate Partner Violence During COVID-19: Prevalence and Correlates. J Interpers Violence.

[47] Aantjes C, Muchanga V, Munguambe K (2022). Exposed and unprotected: Sex worker vulnerabilities during the COVID-19 health emergency in Mozambique. Glob Public Health.

[48] Folayan MO, Arije O, Enemo A (2022). Associations between COVID-19 vaccine hesitancy and the experience of violence among women and girls living with and at risk of HIV in Nigeria. Afr J AIDS Res.

[49] Logie CH, Okumu M, Latif M (2021). Exploring resource scarcity and contextual influences on wellbeing among young refugees in Bidi Bidi refugee settlement, Uganda: findings from a qualitative study. Confl Health.

[50] de Souza Santos D, Bittencourt EA, de Moraes Malinverni AC (2022). Domestic violence against women during the Covid-19 pandemic: A scoping review. Forensic Sci Int Rep.

[51] Viero A, Barbara G, Montisci M (2021). Violence against women in the Covid-19 pandemic: A review of the literature and a call for shared strategies to tackle health and social emergencies. Forensic Sci Int.

[52] Peterman A, Devries K, Guedes A (2023). Ethical reporting of research on violence against women and children: a review of current practice and recommendations for future guidelines. BMJ Glob Health.

[53] Deering KN, Amin A, Shoveller J (2014). A systematic review of the correlates of violence against sex workers. Am J Public Health.

[54] Arango DJ, Morton M, Gennari F (2014). *Interventions to Prevent or Reduce Violence Against Women and Girls: a Systematic Review of Reviews*.

[55] Ellsberg M, Arango DJ, Morton M (2015). Prevention of violence against women and girls: what does the evidence say?. The Lancet.

[56] Stöckl H, Devries K, Rotstein A (2013). The global prevalence of intimate partner homicide: a systematic review. The Lancet.

[57] Abrahams N, Devries K, Watts C (2014). Worldwide prevalence of non-partner sexual violence: a systematic review. The Lancet.

[58] Li Y, Marshall CM, Rees HC (2014). Intimate partner violence and HIV infection among women: a systematic review and meta‐analysis. J Int AIDS Soc.

[59] Devries KM, Mak JY, Bacchus LJ (2013). Intimate partner violence and incident depressive symptoms and suicide attempts: a systematic review of longitudinal studies. PLoS Med.

[60] Devries KM, Child JC, Bacchus LJ (2014). Intimate partner violence victimization and alcohol consumption in women: a systematic review and meta-analysis. Addiction.

[61] Rivas C, Ramsay J, Sadowski L (2016). Advocacy Interventions to Reduce or Eliminate Violence and Promote the Physical and Psychosocial Well‐Being of Women who Experience Intimate Partner Abuse: A Systematic Review. Campbell Syst Rev.

[62] Regehr C, Alaggia R, Dennis J (2013). Interventions to Reduce Distress in Adult Victims of Sexual Violence and Rape: A Systematic Review. Campbell Syst Rev.

[63] Lagdon S, Armour C, Stringer M (2014). Adult experience of mental health outcomes as a result of intimate partner violence victimisation: a systematic review. Eur J Psychotraumatol.

[64] Emezue C, Chase J-AD, Udmuangpia T (2022). Technology-based and digital interventions for intimate partner violence: A systematic review and meta-analysis. *Campbell Syst Rev*.

[65] Rahman MM, Rouyard T, Khan ST (2023). Reproductive, maternal, newborn, and child health intervention coverage in 70 low-income and middle-income countries, 2000-30: trends, projections, and inequities. Lancet Glob Health.

[66] Page MJ, McKenzie JE, Bossuyt PM (2021). The PRISMA 2020 statement: an updated guideline for reporting systematic reviews. BMJ.

