# Supplementary material for: What research evidence is required on violence against women to inform future pandemic preparedness? A scoping review of the research evidence and gaps
Source: BMJ Glob Health. 2024 Dec 11;9(12):e015877. doi: 10.1136/bmjgh-2024-015877 (PMC11647320; doi:10.1136/bmjgh-2024-015877)
Supplement: online supplemental file 1 [file bmjgh-9-12-s001.pdf]

What research evidence is required on Violence Against Women to inform future pandemic preparedness? A scoping review of the research evidence and gaps

Supplementary File

Contents

Appendix A: Preferred Reporting Items for Systematic reviews and Meta-Analyses extension for Scoping Reviews (PRISMA-ScR) Checklist 1

Appendix B: Search Strategy 3

Appendix C: Data Extraction 5

Appendix D: Summary of Included Studies 8

Appendix E: Analysis by Type of Violence Addressed by Studies 80

Appendix F: Analysis by study research design 82

Appendix G: Breakdown of studies that measure self-reported incidents 86

Appendix H: Analysis by thematic topic, income context and research design 87

Appendix I: Analysis by study participants/target groups 89

Appendix J: Analysis by participants' age, gender, sex, sexual orientation 92

Appendix K: Analysis by study setting 95

Appendix L: Analysis by sustainable development goals (SDG) region of studies 97

Appendix M: Analysis by WHO region of studies 98

References (Included Studies) 100

Appendix A: Preferred Reporting Items for Systematic reviews and Meta-Analyses extension for Scoping Reviews (PRISMA-ScR) Checklist

SECTION	ITEM	PRISMA-ScR CHECKLIST ITEM	REPORTED ON PAGE #
**TITLE**			
Title	1	Identify the report as a scoping review.	Title page
**ABSTRACT**			
Structured summary	2	Provide a structured summary that includes (as applicable): background, objectives, eligibility criteria, sources of evidence, charting methods, results, and conclusions that relate to the review questions and objectives.	Page 1
**INTRODUCTION**			
Rationale	3	Describe the rationale for the review in the context of what is already known. Explain why the review questions/objectives lend themselves to a scoping review approach.	Page 4
Objectives	4	Provide an explicit statement of the questions and objectives being addressed with reference to their key elements (e.g., population or participants, concepts, and context) or other relevant key elements used to conceptualize the review questions and/or objectives.	Page 4
**METHODS**			
Protocol and registration	5	Indicate whether a review protocol exists; state if and where it can be accessed (e.g., a Web address); and if available, provide registration information, including the registration number.	Page 6
Eligibility criteria	6	Specify characteristics of the sources of evidence used as eligibility criteria (e.g., years considered, language, and publication status), and provide a rationale.	Page 6
Information sources*	7	Describe all information sources in the search (e.g., databases with dates of coverage and contact with authors to identify additional sources), as well as the date the most recent search was executed.	Page 6
Search	8	Present the full electronic search strategy for at least 1 database, including any limits used, such that it could be repeated.	Supplementary file page X
Selection of sources of evidence†	9	State the process for selecting sources of evidence (i.e., screening and eligibility) included in the scoping review.	Page 6-7
Data charting process‡	10	Describe the methods of charting data from the included sources of evidence (e.g., calibrated forms or forms that have been tested by the team before their use, and whether data charting was done independently or in duplicate) and any processes for obtaining and confirming data from investigators.	Page 6-7
Data items	11	List and define all variables for which data were sought and any assumptions and simplifications made.	Page 7
Critical appraisal of individual sources of evidence§	12	If done, provide a rationale for conducting a critical appraisal of included sources of evidence; describe the methods used and how this information was used in any data synthesis (if appropriate).	N/A
Synthesis of results	13	Describe the methods of handling and summarizing the data that were charted.	Page 7
**RESULTS**			
Selection of sources of evidence	14	Give numbers of sources of evidence screened, assessed for eligibility, and included in the review, with reasons	Page 7

SECTION	ITEM	PRISMA-ScR CHECKLIST ITEM	REPORTED ON PAGE #
		for exclusions at each stage, ideally using a flow diagram.	
Characteristics of sources of evidence	15	For each source of evidence, present characteristics for which data were charted and provide the citations.	Table 1
Critical appraisal within sources of evidence	16	If done, present data on critical appraisal of included sources of evidence (see item 12).	N/A
Results of individual sources of evidence	17	For each included source of evidence, present the relevant data that were charted that relate to the review questions and objectives.	Page 7/ Table 1
Synthesis of results	18	Summarize and/or present the charting results as they relate to the review questions and objectives.	Page 82-93
**DISCUSSION**			
Summary of evidence	19	Summarize the main results (including an overview of concepts, themes, and types of evidence available), link to the review questions and objectives, and consider the relevance to key groups.	Page 94
Limitations	20	Discuss the limitations of the scoping review process.	Page 96
Conclusions	21	Provide a general interpretation of the results with respect to the review questions and objectives, as well as potential implications and/or next steps.	Page 96-97
**FUNDING**			
Funding	22	Describe sources of funding for the included sources of evidence, as well as sources of funding for the scoping review. Describe the role of the funders of the scoping review.	Table 1 & Page 97

JBI = Joanna Briggs Institute; PRISMA-ScR = Preferred Reporting Items for Systematic reviews and Meta-Analyses extension for Scoping Reviews.

* Where *sources of evidence* (see second footnote) are compiled from, such as bibliographic databases, social media platforms, and Web sites.

† A more inclusive/heterogeneous term used to account for the different types of evidence or data sources (e.g., quantitative and/or qualitative research, expert opinion, and policy documents) that may be eligible in a scoping review as opposed to only studies. This is not to be confused with *information sources* (see first footnote).

‡ The frameworks by Arksey and O'Malley (6) and Levac and colleagues (7) and the JBI guidance (4, 5) refer to the process of data extraction in a scoping review as data charting.

§ The process of systematically examining research evidence to assess its validity, results, and relevance before using it to inform a decision. This term is used for items 12 and 19 instead of "risk of bias" (which is more applicable to systematic reviews of interventions) to include and acknowledge the various sources of evidence that may be used in a scoping review (e.g., quantitative and/or qualitative research, expert opinion, and policy document).

From: Tricco AC, Lillie E, Zarin W, O'Brien KK, Colquhoun H, Levac D, et al. PRISMA Extension for Scoping Reviews (PRISMAScR): Checklist and Explanation. Ann Intern Med. 2018;169:467–473. doi: 10.7326/M18-0850.

Appendix B: Search Strategy

The six databases outlined below were searched for studies published between 01/01/2020 and 11/02/2023 (search date).

DATABASE	SEARCH STRING
**Scopus**<br><br>**Search date:** 11/02/2023	Violence* OR abuse* OR harass* OR abusive* OR rape OR sexual* OR victimi* OR doxing OR surveillance OR pile-on OR sex-offense OR offens* OR gaslight* OR troll* OR beat* OR exploit* OR intimid* OR aggress* OR femicide OR female-genital-mutilation OR FGM<br><br>Covid* OR corona* OR COV* OR SARS-CoV-2 OR pandemic OR lockdown<br><br>Wom*n OR girl* OR female* OR mother* OR daughter* OR wife OR wives
**PSYCINFO**<br><br>**Search date:** 11/02/2023	Violence* OR abuse* OR harass* OR abusive* OR rape OR sexual* OR victimi* OR doxing OR surveillance OR pile-on OR sex-offense OR offens* OR gaslight* OR troll* OR beat* OR exploit* OR intimid* OR aggress* OR femicide OR female-genital-mutilation OR FGM<br><br>Covid* OR corona* OR COV* OR SARS-CoV-2 OR pandemic OR lockdown<br><br>Wom*n OR girl* OR female* OR mother* OR daughter* OR wife OR wives
**MEDLINE**<br><br>**Search date:** 11/02/2023	Violence/ OR abuse*OR harass* OR abusive* OR rape OR sexual* OR victimi* OR doxing OR surveillance OR pile-on OR sex-offense OR offens* OR gaslight* OR troll* OR beat* OR exploit* OR intimid* OR aggress* OR femicide OR female-genital-mutilation OR FGM<br><br>Covid* OR corona* OR COV* OR SARS-CoV-2 OR pandemic OR lockdown<br><br>Women OR woman/ OR girl* OR female* OR mother* OR daughter* OR wife OR wives
**Social Science Citation – expanded**<br><br>**Search date:** 11/02/2023	Violence* OR abuse* OR harass* OR abusive* OR rape OR sexual* OR victimi* OR doxing OR surveillance OR pile-on OR sex-offense OR offens* OR gaslight* OR troll* OR beat* OR exploit* OR intimid* OR aggress* OR femicide OR female-genital-mutilation OR FGM<br><br>Covid* OR corona* OR COV* OR SARS-CoV-2 OR pandemic OR lockdown<br><br>Wom*n OR girl* OR female* OR mother* OR daughter* OR wife OR wives
**Cochrane**<br><br>**Search date:** 11/02/2023	Violence/ OR abuse*OR harass* OR abusive* OR rape OR sexual* OR victimi* OR doxing OR surveillance OR pile-on OR sex-offense OR offens* OR gaslight* OR troll* OR beat* OR exploit* OR intimid* OR aggress* OR femicide OR female-genital-mutilation OR FGM

	Covid* OR corona* OR COV* OR SARS-CoV-2 OR pandemic OR lockdown<br><br>Women OR woman/ OR girl* OR female* OR mother* OR daughter* OR wife OR wives
**Campbell**<br><br>**Search date:** 11/02/2023	Violence/ OR abuse*OR harass* OR abusive* OR rape OR sexual* OR victimi* OR doxing OR surveillance OR pile-on OR sex-offense OR offens* OR gaslight* OR troll* OR beat* OR exploit* OR intimid* OR aggress* OR femicide OR female-genital-mutilation OR FGM<br><br>Covid* OR corona* OR COV* OR SARS-CoV-2 OR pandemic OR lockdown<br><br>Women OR woman/ OR girl* OR female* OR mother* OR daughter* OR wife OR wives

Table 1. Search Strategy

Appendix C: Data Extraction

Methods/Study Characteristics

- Ref Detail
  - ID Number
- Funder
- Funded
- Not specified/unclear
- Not funded
- Study Design [Select **primary** study design]
- Quantitative
  - Experimental
  - Quasi-experimental
  - Multi-stage quantitative study
  - Longitudinal quantitative
  - Quantitative media analysis
  - Quantitative Helpline calls analysis
- Qualitative
  - Multi-stage qualitative study
  - Longitudinal qualitative
- Mixed Methods (where any mixed methods are used)
  - Mixed methods primary method
  - Mixed methods secondary method
  - Qualitative media analysis
  - Qualitative Helpline calls analysis
    - Not specified/ Unclear

Data type [select all that apply]

- Primary
- Secondary
  - Administrative

Data collection [select all that apply]

- Face to face
- Online
- Telephone
- Not specified/ Unclear

Sample Size

- N=
- Not specified/ Unclear

Sample Size (Multi-stage studies)

- Primary sample size N= [type]
- Secondary sample size N= [type]
- Tertiary sample size N= [type]
- Not specified/ Unclear

Sample Size (where mixed methods are used)

- Primary sample size N= [type]
- Secondary sample size N= [type]
- Tertiary sample size N= [type]
- Not specified/ Unclear

- Country
  [Select all that apply]
  - Not specified/ Unclear
- WHO Region
  - Africa

- Eastern Mediterranean
- Europe
- South-East Asia
- The Americas
- Western Pacific
- N/A

- SDG Region
  - Central & Southern Asia
  - Eastern & South-Eastern Asia
  - Europe & Northern America
  - Latin America & the Caribbean
  - Northern Africa & Western Asia
  - Oceania
  - Sub-Saharan Africa
- Income Context
  - Lower/Middle-income country
  - High income country
- Setting
  - Community-Urban
  - Community-Rural
  - Community-Mixed
  - Healthcare
  - Education
  - Justice/policing
  - Church/religious
  - Domestic
  - Workplace (more generally)
  - Not specified/ Unclear

Groups/Sample Characteristics

- Participants
  - Survivors
  - Perpetrators
  - Practitioners/policymakers
- Sex reported?
  - Female
  - Male
  - Other
- Gender reported?
  - Women
  - Men
  - Transgender women
  - Transgender men
  - Intersex
  - Non-binary/gender fluid
  - Other
- Age Group (only specify age groups where this is clearly specified in the study design. Otherwise, please select 'Mixed')
  - 15 - 24
  - 25 – 50

- 50 +
- Mixed
- Not specified/ Unclear
- Sexual orientation reported? [Select if sexuality/sexual orientation is clearly reported in the study - as a follow up, select all sexual orientations reported below. If not clearly reported - leave unselected and do select any of the subcategories below.]
    - Heterosexual
    - Gay
    - Lesbian
    - Bisexual
    - LGBTQI+
    - Other
- Target Group
    - Sex workers
    - Study is **primarily** focused on ethnic minority (context specific)
    - Police
    - Healthcare worker/professional
    - Teacher/educator
    - Religious leader
    - Community/youth worker
    - Other (students, public servants, other) - specify
    - Not specified / unclear

Thematic Focus - Topics addressed in the literature

[Select all that apply]

- Prevalence/Incidence
    - Survey
        - Representative Sample
        - Non-representative Sample
    - Administrative/Service level data
    - Media reports
- Experience of violence
- Interventions with survivors to address violence
- Interventions with perpetrators to address violence

Thematic Focus - Type(s) of Violence (Based on WHO definition/SDG alignment)

[Select all that apply]

- Physical violence
- Sexual violence
- Emotional/Psychological
- Acid attacks
- Femicide
- Forced marriage.
- Coercive control
- Other

Modality of violence
[select all that apply]

- Online
- Face to Face/In-Person
- Not specified / unclear

Appendix D: Summary of Included Studies

Ref Code	Author, Year Reference	WHO Region & Income Context	Thematic Focus & Types of Violence	Funding Status	Study design	Data Type	Data Collection Interface	Participants
S001	Aantjes et al., 2022 [1]	• Africa<br>• Lower/ Middle Income Country	• Experience of violence<br>• Physical violence<br>• Sexual violence	• Funded	• Qualitative	• Primary data collection	• Face to face<br>• Telephone	• Survivors<br>• Age 15 - 24
S002	Abdallah et al., 2021 [2]	• The Americas<br>• High Income Country	• Experience of violence<br>• Physical violence	• Funding not specified/Unclear	• Quantitative	• Secondary data analysis	• Online	• Survivors<br>• Mixed Age Groups
S003	Aborisade, 2021 [3]	• Africa<br>• Lower/ Middle Income Country	• Experience of violence<br>• Physical violence<br>• Sexual violence	• Funding not specified/Unclear	• Qualitative	• Primary data collection	• Online<br>• Telephone	• Survivors<br>• Age group(s) not specified/ Unclear
S004	Aborisade, 2022a [4]	• Africa<br>• Lower/ Middle Income Country	• Experience of violence<br>• Sexual violence	• Not funded	• Qualitative	• Primary data collection	• Face to face	• Survivors<br>• Practitioner/ Policymaker<br>• Age 15 - 24
S005	Aborisade, 2022b [5]	• Africa<br>• Lower/ Middle Income Country	• Experience of violence<br>• Sexual violence	• Funding not specified/Unclear	• Qualitative	• Primary data collection	• Face to face<br>• Online<br>• Telephone	• Survivors<br>• Mixed Age Groups
S006	Abrahams & Lund, 2022 [6]	• Africa<br>• Lower/ Middle Income Country	• Experience of violence<br>• Physical violence<br>• Sexual violence<br>• Emotional/Psychological	• Funded	• Quantitative	• Primary data collection	• Face to face	• Survivors<br>• Mixed Age Groups

S007	Abrahams et al., 2022 [7]	• Africa<br>• Lower/ Middle Income Country	• Experience of violence<br>• Physical violence<br>• Sexual violence<br>• Emotional/Psychological	• Funded	• Quantitative	• Primary data collection	• Telephone	• Survivors<br>• Mixed Age Groups
S008	Abu-Elenin et al., 2022 [8]	• Eastern Mediterranean<br>• Lower/ Middle Income Country	• Experience of violence<br>• Physical violence<br>• Sexual violence<br>• Emotional/Psychological<br>• Coercive control	• Funded	• Quantitative	• Primary data collection	• Online	• Survivors<br>• Mixed Age Groups
S009	Abuhammad, 2021 [9]	• Eastern Mediterranean<br>• Lower/ Middle Income Country	• Prevalence/Incidence<br>• Physical violence<br>• Emotional/Psychological	• Funding not specified/Unclear	• Quantitative	• Primary data collection	• Online	• Survivors<br>• Mixed Age Groups
S010	Abujilban et al., 2022 [10]	• Eastern Mediterranean<br>• Lower/ Middle Income Country	• Prevalence/Incidence<br>• Physical violence<br>• Sexual violence<br>• Emotional/Psychological<br>• Coercive control	• Not funded	• Quantitative	• Primary data collection	• Online	• Survivors<br>• Mixed Age Groups
S011	Adams-Clark & Freyd, 2022 [11]	• The Americas<br>• High Income Country	• Prevalence/Incidence<br>• Sexual violence	• Funding not specified/Unclear	• Quantitative	• Primary data collection	• Online	• Survivors<br>• Age 15 - 24
S012	Adibelli et al., 2021 [12]	• Europe<br>• Lower/ Middle Income Country	• Prevalence/Incidence<br>• Physical violence<br>• Sexual violence<br>• Emotional/Psychological<br>• Coercive control	• Funding not specified/Unclear	• Quantitative	• Primary data collection	• Online	• Survivors<br>• Age 15 - 24

S013	Afif et al., 2022 [13]	• The Americas<br><br>• High Income Country	• Prevalence/Incidence<br><br>• Physical violence<br>• Femicide	• Not funded	• Quantitative	• Secondary data analysis	• Online	• Survivors<br><br>• Age 15 - 24
S014	Aghamohseni et al., 2021 [14]	• Eastern Mediterranean<br><br>• Lower/ Middle Income Country	• Prevalence/Incidence<br><br>• Physical violence<br>• Sexual violence<br>• Emotional/Psychological<br>• Coercive control	• Not funded	• Quantitative	• Primary data collection	• Online	• Survivors<br><br>• Mixed Age Groups
S015	Aguero, 2021 [15]	• The Americas<br><br>• Lower/ Middle Income Country	• Prevalence/Incidence<br><br>• Physical violence<br>• Sexual violence<br>• Other	• Funding not specified/Unclear	• Quantitative	• Secondary data analysis	• Telephone	• Survivors<br><br>• Age group(s) not specified/ Unclear
S016	Akalin & Ayhan, 2022 [16]	• Europe<br><br>• Lower/ Middle Income Country	• Prevalence/Incidence<br><br>• Physical violence<br>• Sexual violence<br>• Emotional/Psychological<br>• Coercive control	• Funding not specified/Unclear	• Quantitative	• Primary data collection	• Online	• Survivors<br><br>• Mixed Age Groups
S017	Akel et al., 2022 [17]	• Eastern Mediterranean<br><br>• Lower/ Middle Income Country	• Prevalence/Incidence<br><br>• Physical violence<br>• Sexual violence<br>• Emotional/Psychological	• Not funded	• Quantitative	• Primary data collection	• Face to face	• Survivors<br><br>• Mixed Age Groups
S018	Alcantara-Lopez et al., 2022 [18]	• Europe<br><br>• High Income Country	• Experience of violence<br><br>• Physical violence<br>• Emotional/Psychological	• Funded	• Quantitative	• Primary data collection	• Telephone	• Survivors<br><br>• Mixed Age Groups

S019	Alexandru et al., 2021 [19]	• Europe<br><br>• Lower/ Middle Income Country	• Prevalence/Incidence<br><br>• Other	• Funding not specified/Unclear	• Qualitative	• Primary data collection	• Online	• Practitioner/ Policymaker<br><br>• Mixed Age Groups
S020	Alharbi et al., 2021 [20]	• Eastern Mediterranean<br><br>• High Income Country	• Prevalence/Incidence<br><br>• Physical violence<br>• Sexual violence<br>• Emotional/Psychological	• Not funded	• Quantitative	• Secondary data analysis	• Online	• Survivors<br><br>• Mixed Age Groups
S021	Ali & Khalid, 2021 [21]	• Eastern Mediterranean<br><br>• Lower/ Middle Income Country	• Experience of violence<br><br>• Physical violence<br>• Emotional/Psychological<br>• Coercive control	• Funding not specified/Unclear	• Qualitative	• Secondary data analysis	• Online	• Survivors<br><br>• Age group(s) not specified/ Unclear
S022	Alimoradi et al., 2023 [22]	• Eastern Mediterranean<br><br>• Lower/ Middle Income Country	• Prevalence/Incidence<br><br>• Other	• Funded	• Quantitative	• Primary data collection	• Online	• Survivors<br><br>• Mixed Age Groups
S023	Al-Rawi et al., 2021 [23]	• N/A	• Experience of violence<br><br>• Other	• Funded	• Qualitative	• Secondary data analysis	• Not specified/unclear	• Not specified/ Unclear<br><br>• Age group(s) not specified/ Unclear

S024	Anam et al., 2021 [24]	• Western Pacific<br><br>• Lower/ Middle Income Country	• Experience of violence<br><br>• Physical violence<br>• Emotional/Psychological<br>• Coercive control	• Funding not specified/Unclear	• Qualitative	• Secondary data analysis	• Online	• Survivors<br><br>• Age group(s) not specified/ Unclear
S025	Andreeva et al., 2021 [25]	• Europe<br><br>• Lower/ Middle Income Country	• Experience of violence<br><br>• Emotional/Psychological<br>• Coercive control<br>• Other	• Not funded	• Qualitative	• Secondary data analysis	• Online	• Not specified/ Unclear<br><br>• Age group(s) not specified/ Unclear
S026	Angwenyi et al., 2021 [26]	• Africa<br><br>• Lower/ Middle Income Country	• Experience of violence<br><br>• Emotional/Psychological	• Funded	• Quantitative	• Primary data collection	• Telephone	• Survivors<br><br>• Mixed Age Groups
S027	Anitha & Gill, 2022 [27]	• Europe<br><br>• High Income Country	• Experience of violence<br><br>• Physical violence<br>• Sexual violence<br>• Emotional/Psychological<br>• Forced marriage<br>• Other	• Funded	• Qualitative	• Primary data collection	• Online	• Practitioner/ Policymaker<br><br>• Age group(s) not specified/ Unclear
S028	Aolymat, 2021 [28]	• Eastern Mediterranean<br><br>• Lower/ Middle Income Country	• Prevalence/Incidence<br><br>• Other	• Funding not specified/Unclear	• Quantitative	• Primary data collection	• Online	• Survivors<br><br>• Mixed Age Groups
S029	Arafa et al., 2022 [29]	• Eastern Mediterranean<br><br>• Lower/ Middle Income Country	• Prevalence/Incidence<br><br>• Physical violence<br>• Emotional/Psychological	• Not funded	• Quantitative	• Primary data collection	• Face to face	• Survivors<br><br>• Mixed Age Groups

S030	Araujo-Chaveron et al., 2022 [30]	• Europe<br><br>• High Income Country	• Prevalence/Incidence<br><br>• Other	• Not funded	• Quantitative	• Primary data collection	• Online	• Survivors<br><br>• Mixed Age Groups
S031	Archer-Kuhn et al., 2023 [31]	• The Americas<br><br>• High Income Country	• Experience of violence<br><br>• Emotional/Psychological<br>• Coercive control	• Funded	• Qualitative	• Primary data collection	• Online	• Survivors<br><br>• Mixed Age Groups
S032	Aristegui et al., 2022 [32]	• The Americas<br><br>• Lower/ Middle Income Country	• Prevalence/Incidence<br><br>• Sexual violence<br>• Coercive control	• Funded	• Quantitative	• Primary data collection	• Online	• Survivors<br><br>• Mixed Age Groups
S033	Asadi et al., 2022 [33]	• Eastern Mediterranean<br><br>• Lower/ Middle Income Country	• Experience of violence<br><br>• Physical violence<br>• Sexual violence<br>• Emotional/Psychological<br>• Coercive control	• Not funded	• Quantitative	• Primary data collection	• Online	• Survivors<br><br>• Mixed Age Groups
S034	Asik & Ozen, 2021 [34]	• Eastern Mediterranean<br><br>• Lower/ Middle Income Country	• Prevalence/Incidence<br><br>• Femicide	• Funding not specified/Unclear	• Quantitative	• Secondary data analysis	• Not specified/unclear	• Survivors<br><br>• Age group(s) not specified/ Unclear

S035	Asratie, 2022 [35]	• Africa<br><br>• Lower/ Middle Income Country	• Prevalence/Incidence<br><br>• Physical violence<br>• Sexual violence<br>• Emotional/Psychological	• Funding not specified/Unclear	• Quantitative	• Primary data collection	• Face to face	• Survivors<br><br>• Mixed Age Groups
S036	Atilla et al., 2023 [36]	• Europe<br><br>• Lower/ Middle Income Country	• Prevalence/Incidence<br><br>• Physical violence<br>• Sexual violence<br>• Emotional/Psychological<br>• Coercive control	• Not funded	• Quantitative	• Primary data collection	• Face to face	• Survivors<br><br>• Mixed Age Groups
S037	Atuhaire et al., 2021 [37]	• Africa<br><br>• Lower/ Middle Income Country	• Experience of violence<br><br>• Physical violence<br>• Emotional/Psychological	• Funded	• Qualitative	• Primary data collection	• Face to face	• Survivors<br><br>• Mixed Age Groups
S038	Augusti et al., 2021 [38]	• Europe<br><br>• High Income Country	• Prevalence/Incidence<br><br>• Physical violence<br>• Sexual violence<br>• Emotional/Psychological<br>• Other	• Funded	• Quantitative	• Primary data collection	• Online	• Survivors<br><br>• Age 15 - 24
S039	Bagheri Lankarani et al., 2022 [39]	• Eastern Mediterranean<br><br>• Lower/ Middle Income Country	• Prevalence/Incidence<br><br>• Physical violence<br>• Sexual violence<br>• Emotional/Psychological<br>• Coercive control	• Funded	• Quantitative	• Primary data collection	• Online	• Survivors<br>• Perpetrators<br><br>• Mixed Age Groups

S040	Baidoo et al., 2021 [40]	• The Americas<br><br>• High Income Country	• Prevalence/Incidence<br><br>• Other	• Funded	• Quantitative	• Secondary data analysis	• Not specified/unclear	• Survivors<br><br>• Mixed Age Groups
S041	Ballester-Arnal et al., 2021 [41]	• Europe<br><br>• High Income Country	• Prevalence/Incidence<br><br>• Sexual violence	• Funding not specified/Unclear	• Quantitative	• Primary data collection	• Online	• Survivors<br>• Perpetrators<br><br>• Mixed Age Groups
S042	Balmori de la Miyar et al., 2022 [42]	• The Americas<br><br>• Lower/ Middle Income Country	• Experience of violence *use of violence as a strategy for conflict resolution*<br><br>• Physical violence	• Funding not specified/Unclear	• Quantitative	• Secondary data analysis	• Not specified/unclear	• Perpetrators<br><br>• Age group(s) not specified/ Unclear
S043	Banerjee et al., 2022 [43]	• South-East Asia<br><br>• Lower/ Middle Income Country	• Experience of violence<br><br>• Other	• Funding not specified/Unclear	• Qualitative	• Secondary data analysis	• Online	• Not specified/ Unclear<br><br>• Age group(s) not specified/ Unclear
S044	Bankovskaya et al., 2021 [44]	• Eastern Mediterranean<br><br>• Lower/ Middle Income Country	• Prevalence/Incidence<br><br>• Physical violence<br>• Emotional/Psychological<br>• Coercive control	• Funding not specified/Unclear	• Qualitative	• Primary data collection	• Online<br>• Telephone	• Survivors<br><br>• Mixed Age Groups
S045	Barchielli et al., 2021 [45]	• Europe	• Prevalence/Incidence<br><br>• Physical violence	• Not funded	• Quantitative	• Secondary data analysis	• Not specified/unclear	• Survivors<br>• Perpetrators

		• High Income Country	• Sexual violence<br>• Emotional/Psychological<br>• Coercive control					• Mixed Age Groups
S046	Belay et al., 2022 [46]	• Africa<br><br>• Lower/ Middle Income Country	• Prevalence/Incidence<br><br>• Physical violence<br>• Sexual violence<br>• Emotional/Psychological<br>• Coercive control	• Funded	• Quantitative	• Primary data collection	• Face to face	• Survivors<br><br>• Mixed Age Groups
S047	Bevilacqua et al., 2022 [47]	• Africa<br><br>• Lower/ Middle Income Country	• Prevalence/Incidence<br><br>• Sexual violence	• Funded	• Quantitative	• Primary data collection	• Telephone	• Survivors<br><br>• Mixed Age Groups
S048	Bhandari et al., 2022 [48]	• Western Pacific<br><br>• Lower/ Middle Income Country	• Experience of violence<br><br>• Physical violence<br>• Emotional/Psychological	• Funded	• Quantitative	• Primary data collection	• Online	• Survivors<br><br>• Mixed Age Groups
S049	Bhattaram et al., 2022 [49]	• South-East Asia<br><br>• Lower/ Middle Income Country	• Prevalence/Incidence<br><br>• Other	• Not funded	• Quantitative	• Secondary data analysis	• Not specified/unclear	• Survivors<br><br>• Mixed Age Groups
S050	Bidstrup et al., 2022 [50]	• Europe<br><br>• High Income Country	• Prevalence/Incidence<br><br>• Sexual violence	• Funding not specified/Unclear	• Quantitative	• Secondary data analysis	• Face to face	• Survivors<br><br>• Mixed Age Groups
S051	Biehler-Gomez et al., 2022 [51]	• Europe<br><br>• High Income Country	• Prevalence/Incidence<br><br>• Femicide	• Funded	• Quantitative	• Secondary data analysis	• Not specified/unclear	• Survivors<br><br>• Mixed Age Groups

S052	Blair et al., 2022 [52]	• The Americas<br>• High Income Country	• Prevalence/Incidence<br>• Other	• Not funded	• Quantitative	• Primary data collection	• Online<br>• Telephone	• Survivors<br>• Mixed Age Groups
S053	Boyd et al., 2022 [53]	• The Americas<br>• High Income Country	• Prevalence/Incidence<br>• Physical violence<br>• Emotional/Psychological	• Funded	• Quantitative	• Primary data collection	• Online	• Survivors<br>• Mixed Age Groups
S054	Brody et al., 2023 [54]	• Western Pacific<br>• Lower/ Middle Income Country	• Prevalence/Incidence<br>• Physical violence<br>• Sexual violence<br>• Emotional/Psychological<br>• Coercive control	• Funded	• Quantitative	• Primary data collection	• Telephone	• Survivors<br>• Mixed Age Groups
S055	Brown et al., 2021 [55]	• Europe<br>• High Income Country	• Prevalence/Incidence<br>• Physical violence<br>• Sexual violence<br>• Emotional/Psychological<br>• Acid attacks<br>• Coercive control	• Not funded	• Quantitative	• Secondary data analysis	• Not specified/unclear	• Survivors<br>• Mixed Age Groups
S056	Buchner et al., 2023 [56]	• Europe<br>• High Income Country	• Experience of violence<br>• Other	• Not funded	• Quantitative	• Secondary data analysis	• Online	• Survivors<br>• Not specified/ Unclear<br>• Age group(s) not specified/ Unclear
S057	Bueso-Izquierdo et al., 2022 [57]	• Europe<br>• High Income Country	• Experience of violence<br>• Other	• Funding not specified/Unclear	• Quantitative	• Primary data collection	• Face to face	• Survivors<br>• Age 25 - 50

S058	Bukuluki et al., 2023 [58]	• Africa<br><br>• Lower/ Middle Income Country	• Experience of violence<br><br>• Physical violence<br>• Sexual violence<br>• Emotional/Psychological<br>• Forced marriage<br>• Coercive control<br>• Other	• Funded	• Quantitative	• Secondary data analysis	• Online<br>• Telephone	• Survivors<br>• Not specified/ Unclear<br><br>• Mixed Age Groups
S059	Çağlar et al., 2021 [59]	• Europe<br><br>• Lower/ Middle Income Country	• Prevalence/Incidence<br><br>• Physical violence<br>• Emotional/Psychological	• Not funded	• Quantitative	• Primary data collection	• Online	• Survivors<br><br>• Mixed Age Groups
S060	Cameron et al., 2021 [60]	• The Americas<br><br>• High Income Country	• Prevalence/Incidence<br><br>• Physical violence<br>• Sexual violence<br>• Emotional/Psychological<br>• Femicide<br>• Forced marriage<br>• Coercive control	• Funding not specified/Unclear	• Quantitative	• Secondary data analysis	• Not specified/unclear	• Survivors<br><br>• Mixed Age Groups
S061	C. E. Cannon et al., 2022 [61]	• The Americas<br><br>• High Income Country	• Experience of violence<br><br>• Physical violence<br>• Sexual violence<br>• Emotional/Psychological<br>• Coercive control	• Funding not specified/Unclear	• Quantitative	• Primary data collection	• Online	• Survivors<br><br>• Mixed Age Groups
S062	Cantor et al., 2022[62]	• The Americas<br><br>• High Income Country	• Prevalence/Incidence<br><br>• Femicide	• Funded	• Quantitative	• Secondary data analysis	• Not specified/unclear	• Survivors<br><br>• Mixed Age Groups

S063	Caridade et al., 2021 [63]	• Europe<br><br>• High Income Country	• Interventions/services with Survivors to address violence<br><br>• Other	• Funded	• Quantitative	• Primary data collection	• Online	• Practitioner/ Policymaker<br><br>• Age group(s) not specified/ Unclear
S064	Cano-Lozano et al., 2021 [64]	• Europe<br><br>• High Income Country	• Prevalence/Incidence<br><br>• Physical violence<br>• Emotional/Psychological<br>• Coercive control	• Funded	• Quantitative	• Primary data collection	• Online	• Perpetrators<br><br>• Mixed Age Groups
S065	Carrington et al., 2021 [65]	• Western Pacific<br><br>• High Income Country	• Interventions/services with Survivors to address violence<br><br>• Emotional/Psychological<br>• Coercive control	• Funded	• Quantitative	• Primary data collection	• Online	• Survivors<br>• Practitioner/ Policymaker<br><br>• Mixed Age Groups
S067	Casanovas et al., 2022 [66]	• Europe<br><br>• High Income Country	• Prevalence/Incidence<br><br>• Sexual violence	• Funded	• Quantitative	• Primary data collection	• Online	• Survivors<br><br>• Age 15 - 24<br>• Age 25 - 50
S068	Chandan et al., 2021 [67]	• Europe<br><br>• High Income Country	• Experience of violence<br><br>• Other	• Not funded	• Quantitative	• Secondary data analysis	• Online	• Survivors<br><br>• Mixed Age Groups

S069	Chang et al., 2022 [68]	• Western Pacific<br><br>• Lower/ Middle Income Country	• Interventions/services with Survivors to address violence<br><br>• Physical violence<br>• Emotional/Psychological	• Funded	• Quantitative	• Primary data collection	• Online	• Survivors<br><br>• Age 15 - 24<br>• Age 25 - 50
S070	Chhabra et al., 2022 [69]	• South-East Asia<br><br>• Lower/ Middle Income Country	• Prevalence/Incidence<br><br>• Other	• Not funded	• Quantitative	• Primary data collection	• Face to face	• Survivors
S071	Chimbindi et al., 2022 [70]	• Africa<br><br>• Lower/ Middle Income Country	• Experience of violence<br><br>• Physical violence<br>• Sexual violence	• Funded	• Qualitative	• Primary data collection	• Telephone	• Survivors<br><br>• Age 15 - 24
S072	Chime et al., 2022 [71]	• Africa<br><br>• Lower/ Middle Income Country	• Prevalence/Incidence<br><br>• Physical violence<br>• Sexual violence<br>• Emotional/Psychological<br>• Other	• Funding not specified/Unclear	• Quantitative	• Secondary data analysis	• Not specified/unclear	• Survivors<br><br>• Mixed Age Groups
S073	Chowdhury et al., 2021 [72]	• South-East Asia<br><br>• Lower/ Middle Income Country	• Experience of violence<br><br>• Emotional/Psychological	• Not funded	• Quantitative	• Primary data collection	• Online	• Survivors<br><br>• Age 15 - 24<br>• Age 25 - 50
S074	Chowdhury et al., 2022 [73]	• South-East Asia<br><br>• Lower/ Middle Income Country	• Experience of violence<br><br>• Physical violence<br>• Emotional/Psychological	• Not funded	• Quantitative	• Primary data collection	• Face to face<br>• Online	• Survivors<br><br>• Mixed Age Groups

S075	Clisby & Choudhury, 2022 [74]	• South-East Asia<br><br>• Lower/ Middle Income Country	• Experience of violence<br><br>• Physical violence<br>• Sexual violence<br>• Coercive control	• Funded	• Qualitative	• Primary data collection	• Face to face	• Survivors<br><br>• Mixed Age Groups
S076	Cornell et al., 2022 [75]	• Lower/ Middle Income Country	• Experience of violence<br><br>• Other	• Funded	• Mixed Methods	• Secondary data analysis	• Not specified/unclear	• Survivors<br><br>• Mixed Age Groups
S077	Cortis et al., 2021 [76]	• Western Pacific<br><br>• High Income Country	• Prevalence/Incidence<br>• Interventions/services with Survivors to address violence<br>• Interventions/services with perpetrators to address violence<br><br>• Other	• Funded	• Mixed Methods	• Primary data collection	• Online	• Practitioner/ Policymaker<br><br>• Age group(s) not specified/ Unclear
S078	Costa et al., 2022 [77]	• Europe<br><br>• Lower/ Middle Income Country<br>• High Income Country	• Experience of violence<br><br>• Physical violence<br>• Emotional/Psychological	• Funded	• Quantitative	• Primary data collection	• Online	• Survivors<br><br>• Age 15 - 24<br>• Age 25 - 50
S079	Cunha et al., 2023 [78]	• Europe<br><br>• High Income Country	• Prevalence/Incidence<br><br>• Physical violence<br>• Sexual violence<br>• Emotional/Psychological<br>• Coercive control	• Funded	• Quantitative	• Primary data collection	• Online	• Survivors<br>• Perpetrators<br><br>• Mixed Age Groups

S080	Das et al., 2021 [79]	• South-East Asia<br><br>• Lower/ Middle Income Country	• Experience of violence<br><br>• Physical violence<br>• Emotional/Psychological	• Not funded	• Quantitative	• Primary data collection	• Face to face	• Survivors<br><br>• Age 15 - 24<br>• Age 25 - 50
S081	Daugherty et al., 2022 [80]	• Europe<br><br>• High Income Country	• Experience of violence<br><br>• Physical violence<br>• Sexual violence<br>• Emotional/Psychological	• Not funded	• Quantitative	• Primary data collection	• Telephone<br>• Not specified/unclear	• Survivors<br><br>• Age 15 - 24<br>• Age 25 - 50
S082	Davidson et al., 2023 [81]	• The Americas<br><br>• High Income Country	• Prevalence/Incidence<br><br>• Physical violence<br>• Sexual violence<br>• Emotional/Psychological	• Not funded	• Quantitative	• Primary data collection	• Online	• Survivors<br><br>• Mixed Age Groups
S083	Day et al., 2023 [82]	• Europe<br><br>• High Income Country	• Prevalence/Incidence<br><br>• Physical violence<br>• Sexual violence<br>• Emotional/Psychological<br>• Coercive control	• Not funded	• Quantitative	• Secondary data analysis	• Online	• Survivors<br><br>• Mixed Age Groups
S084	de Gennaro et al., 2022 [83]	• Europe<br><br>• High Income Country	• Experience of violence<br><br>• Emotional/Psychological	• Funding not specified/Unclear	• Qualitative	• Primary data collection	• Face to face	• Practitioner/ Policymaker<br><br>• Age group(s) not specified/ Unclear

S085	De la Cerda-Vargas et al., 2022 [84]	• The Americas<br><br>• Lower/ Middle Income Country	• Prevalence/Incidence<br><br>• Physical violence<br>• Sexual violence<br>• Emotional/Psychological	• Funding not specified/Unclear	• Quantitative	• Primary data collection	• Online	• Survivors<br><br>• Age 25 - 50
S086	de Oliveira et al., 2021 [85]	• The Americas<br><br>• Lower/ Middle Income Country	• Prevalence/Incidence<br><br>• Physical violence<br>• Sexual violence	• Not funded	• Quantitative	• Secondary data analysis	• Online	• Survivors<br>• Perpetrators<br><br>• Age 15 - 24
S087	Basílio de Simões et al., 2022 [86]	• Europe<br><br>• High Income Country	• Prevalence/Incidence<br>• Experience of violence<br><br>• Sexual violence<br>• Emotional/Psychological	• Funded	• Qualitative	• Primary data collection	• Online	• Survivors<br><br>• Age 15 - 24<br>• Age 25 - 50
S088	Decker et al., 2021 [87]	• Africa<br><br>• Lower/ Middle Income Country	• Prevalence/Incidence<br><br>• Physical violence<br>• Sexual violence<br>• Other	• Funding not specified/Unclear	• Mixed Methods	• Primary data collection	• Telephone	• Survivors<br><br>• Age 15 - 24<br>• Age 25 - 50
S089	Decker, Wood, et al., 2022 [88]	• Africa<br><br>• Lower/ Middle Income Country	• Prevalence/Incidence<br><br>• Physical violence<br>• Sexual violence<br>• Emotional/Psychological	• Funded	• Quantitative	• Primary data collection	• Not specified/unclear	• Survivors<br><br>• Mixed Age Groups

S090	Decker, Bevilacqua, et al., 2022 [89]	• Africa<br><br>• Lower/ Middle Income Country	• Prevalence/Incidence<br><br>• Physical violence<br>• Sexual violence<br>• Emotional/Psychological<br>• Coercive control	• Funded	• Mixed Methods	• Primary data collection	• Online<br>• Telephone	• Survivors<br>• Practitioner/ Policymaker<br><br>• Age 15 - 24
S091	Del Casale et al., 2022 [90]	• Europe<br><br>• High Income Country	• Experience of violence<br><br>• Other	• Funded	• Quantitative	• Secondary data analysis	• Not specified/unclear	• Survivors<br><br>• Age group(s) not specified/ Unclear
S092	Desai et al., 2022 [91]	• Europe<br><br>• High Income Country	• Experience of violence<br><br>• Emotional/Psychological<br>• Coercive control	• Funded	• Qualitative	• Primary data collection	• Telephone	• Survivors<br><br>• Age 25 - 50<br>• Age 50 +
S093	Devoto et al., 2022 [92]	• The Americas<br><br>• High Income Country	• Experience of violence<br><br>• Other	• Funding not specified/Unclear	• Quantitative	• Primary data collection	• Online	• Survivors<br><br>• Mixed Age Groups
S094	Dholakia et al., 2022 [93]	• The Americas<br><br>• High Income Country	• Prevalence/Incidence<br><br>• Physical violence<br>• Emotional/Psychological	• Funding not specified/Unclear	• Quantitative	• Primary data collection	• Online	• Survivors<br><br>• Age group(s) not specified/ Unclear
S095	Di Franco et al., 2021 [94]	• Europe<br><br>• High Income Country	• Experience of violence<br><br>• Other	• Funded	• Quantitative	• Primary data collection<br>• Secondary data analysis	• Face to face	• Survivors<br><br>• Mixed Age Groups

S096	Diaz et al., 2022 [95]	• The Americas<br><br>• High Income Country	• Experience of violence<br><br>• Sexual violence<br>• Other	• Funded	• Quantitative	• Primary data collection	• Online	• Survivors<br><br>• Age 15 - 24<br>• Age 25 - 50
S097	Ditekemena et al., 2021 [96]	• Africa<br><br>• Lower/ Middle Income Country	• Experience of violence<br><br>• Physical violence<br>• Sexual violence<br>• Emotional/Psychological	• Funded	• Quantitative	• Primary data collection	• Online	• Survivors<br><br>• Mixed Age Groups
S098	Dogar et al., 2022 [97]	• Eastern Mediterranean<br><br>• Lower/ Middle Income Country	• Experience of violence<br><br>• Other	• Not funded	• Qualitative	• Primary data collection	• Face to face<br>• Telephone	• Survivors
S099	Domínguez D et al., 2022 [98]	• Western Pacific<br><br>• High Income Country	• Prevalence/Incidence<br><br>• Physical violence<br>• Other	• Funded	• Quantitative	• Secondary data analysis	• Not specified/unclear	• Survivors<br><br>• Mixed Age Groups
S100	Doncarli et al., 2021 [99]	• Europe<br><br>• High Income Country	• Experience of violence<br><br>• Other	• Funded	• Quantitative	• Primary data collection	• Online	• Survivors<br><br>• Age 15 - 24<br>• Age 25 - 50
S101	dos Santos et al., 2022 [100]	• The Americas<br><br>• Lower/ Middle Income Country	• Prevalence/Incidence<br><br>• Femicide	• Funded	• Quantitative	• Secondary data analysis	• Not specified/unclear	• Survivors<br><br>• Mixed Age Groups

S102	Drandić et al., 2022 [101]	• Europe<br><br>• Lower/ Middle Income Country<br>• High Income Country	• Experience of violence<br><br>• Physical violence<br>• Emotional/Psychological	• Funded	• Quantitative	• Primary data collection	• Online	• Survivors<br><br>• Age 15 - 24<br>• Age 25 - 50
S103	Drotning et al., 2023 [102]	• The Americas<br><br>• High Income Country	• Prevalence/Incidence<br><br>• Physical violence<br>• Emotional/Psychological<br>• Coercive control<br>• Other	• Funded	• Quantitative	• Primary data collection	• Online	• Survivors<br><br>• Mixed Age Groups
S104	Du & Chen, 2021 [103]	• Western Pacific<br><br>• Lower/ Middle Income Country	• Prevalence/Incidence<br><br>• Physical violence<br>• Emotional/Psychological<br>• Coercive control	• Funded	• Quantitative	• Primary data collection	• Face to face	• Survivors<br><br>• Age 50 +
S105	Duncan et al., 2022 [104]	• The Americas<br><br>• High Income Country	• Experience of violence<br><br>• Other	• Funded	• Quantitative	• Primary data collection	• Online	• Survivors<br><br>• Age 15 - 24<br>• Age 25 - 50
S106	Eaton et al., 2023 [105]	• The Americas<br><br>• High Income Country	• Prevalence/Incidence<br><br>• Physical violence<br>• Sexual violence<br>• Emotional/Psychological	• Funded	• Quantitative	• Primary data collection	• Online	• Survivors<br><br>• Mixed Age Groups

S107	Ebert & Steinert, 2021 [106]	• Europe<br><br>• High Income Country	• Prevalence/Incidence<br><br>• Physical violence<br>• Sexual violence<br>• Emotional/Psychological<br>• Coercive control	• Funded	• Quantitative	• Primary data collection	• Online	• Survivors<br><br>• Mixed Age Groups
S108	Ebrahimi Rigi et al., 2022 [107]	• Eastern Mediterranean<br><br>• Lower/ Middle Income Country	• Experience of violence<br><br>• Physical violence<br>• Sexual violence<br>• Emotional/Psychological	• Funding not specified/Unclear	• Qualitative	• Primary data collection	• Face to face	• Survivors<br><br>• Mixed Age Groups
S109	Ekawati et al., 2022 [108]	• South-East Asia<br><br>• Lower/ Middle Income Country	• Prevalence/Incidence<br><br>• Physical violence	• Funding not specified/Unclear	• Quantitative	• Primary data collection	• Online	• Survivors<br><br>• Mixed Age Groups
S110	Elhadi et al., 2020 [109]	• Eastern Mediterranean<br><br>• Lower/ Middle Income Country	• Experience of violence<br><br>• Physical violence<br>• Emotional/Psychological	• Funding not specified/Unclear	• Quantitative	• Primary data collection	• Online	• Survivors<br>• Mixed Age Groups
S111	Elhadi et al., 2021 [110]	• Eastern Mediterranean<br><br>• Lower/ Middle Income Country	• Experience of violence<br><br>• Other	• Funding not specified/Unclear	• Quantitative	• Primary data collection	• Online	• Survivors<br><br>• Age 15 - 24<br>• Age 25 - 50

S112	El-Nimr et al., 2021 [111]	• Eastern Mediterranean<br>• N/A<br><br>• Lower/ Middle Income Country<br>• High Income Country	• Prevalence/Incidence<br><br>• Physical violence<br>• Sexual violence<br>• Emotional/Psychological<br>• Coercive control<br>• Other	• Not funded	• Quantitative	• Primary data collection	• Online	• Survivors<br><br>• Mixed Age Groups
S113	Elsaid, Shehata, et al., 2022 [112]	• Eastern Mediterranean<br><br>• Lower/ Middle Income Country	• Prevalence/Incidence<br><br>• Physical violence<br>• Sexual violence<br>• Emotional/Psychological	• Not funded	• Quantitative	• Primary data collection	• Online	• Survivors<br><br>• Mixed Age Groups
S114	Elsaid, Ibrahim, et al., 2022 [113]	• Eastern Mediterranean<br><br>• Lower/ Middle Income Country	• Experience of violence<br><br>• Physical violence	• Not funded	• Quantitative	• Primary data collection	• Online	• Survivors<br><br>• Mixed Age Groups
S115	Endler et al., 2021 [114]	• Africa<br>• Europe<br>• South-East Asia<br>• The Americas<br>• Western Pacific	• Prevalence/Incidence<br><br>• Other	• Funding not specified/Unclear	• Mixed Methods	• Primary data collection	• Online	• Practitioner/ Policymaker<br><br>• Age group(s) not specified/ Unclear

S116	Erausquin et al., 2022 [115]	• Africa<br>• Eastern Mediterranean<br>• Europe<br>• The Americas<br>• Western Pacific<br><br>• Lower/ Middle Income Country<br>• High Income Country	• Prevalence/Incidence<br><br>• Physical violence<br>• Sexual violence	• Funded	• Quantitative	• Primary data collection	• Online	• Survivors<br><br>• Mixed Age Groups
S117	Esho et al., 2022 [116]	• Africa<br><br>• Lower/ Middle Income Country<br>• High Income Country	• Prevalence/Incidence<br><br>• Forced marriage<br>• Other	• Funded	• Mixed Methods	• Primary data collection	• Face to face	• Survivors<br>• Practitioner/ Policymaker<br><br>• Age 15 - 24<br>• Age 25 - 50
S118	Esposito & Szypulska, 2022 [117]	• Europe<br><br>• Lower/ Middle Income Country<br>• High Income Country	• Interventions/services with survivors to address violence<br><br>• Other	• Funded	• Mixed Methods	• Primary data collection	• Online	• Practitioner/ Policymaker<br><br>• Age group(s) not specified/ Unclear
S119	Evans et al., 2021 [118]	• Europe<br><br>• Lower/ Middle Income Country	• Prevalence/Incidence<br><br>• Other	• Not funded	• Quantitative	• Secondary data analysis	• Online	• Survivors<br><br>• Age group(s) not specified/ Unclear
S120	Evcili & Demirel, 2022 [119]	• Europe<br><br>• Lower/ Middle Income Country	• Experience of violence<br><br>• Physical violence<br>• Sexual violence<br>• Other	• Not funded	• Quantitative	• Primary data collection	• Online	• Survivors<br><br>• Mixed Age Groups

S121	Fedina et al., 2023 [120]	• The Americas<br><br>• High Income Country	• Prevalence/Incidence<br><br>• Physical violence<br>• Sexual violence<br>• Emotional/Psychological<br>• Coercive control<br>• Other	• Funded	• Quantitative	• Primary data collection	• Online	• Survivors<br><br>• Mixed Age Groups
S122	Fereidooni et al., 2023 [121]	• Eastern Mediterranean<br><br>• Lower/ Middle Income Country	• Prevalence/Incidence<br><br>• Physical violence<br>• Sexual violence<br>• Emotional/Psychological	• Funded	• Quantitative	• Primary data collection	• Telephone	• Survivors<br><br>• Mixed Age Groups
S123	Ferragina et al., 2022 [122]	• Europe<br><br>• High Income Country	• Prevalence/Incidence<br><br>• Physical violence	• Not funded	• Quantitative	• Secondary data analysis	• Not specified/unclear	• Survivors<br><br>• Age group(s) not specified/ Unclear
S124	Fetene et al., 2022 [123]	• Africa<br><br>• Lower/ Middle Income Country	• Prevalence/Incidence<br><br>• Physical violence<br>• Sexual violence<br>• Emotional/Psychological	• Funded	• Quantitative	• Primary data collection	• Face to face	• Survivors<br><br>• Age 15 - 24<br>• Age 25 - 50
S125	Fianu et al., 2022[124]	• Europe<br><br>• High Income Country	• Experience of violence<br><br>• Emotional/Psychological<br>• Coercive control	• Funded	• Quantitative	• Primary data collection	• Telephone	• Survivors<br><br>• Mixed Age Groups

S126	Fitrianingsih & Saki, 2022 [125]	• South-East Asia<br><br>• Lower/ Middle Income Country	• Experience of violence<br><br>• Physical violence<br>• Sexual violence<br>• Emotional/Psychological	• Funded	• Quantitative	• Primary data collection	• Face to face	• Survivors<br><br>• Age 15 - 24<br>• Age 25 - 50
S127	Fleming & Franzese, 2021 [126]	• The Americas<br><br>• High Income Country	• Experience of violence<br><br>• Emotional/Psychological	• Funding not specified/Unclear	• Quantitative	• Primary data collection	• Online	• Survivors<br><br>• Age group(s) not specified/ Unclear
S128	Focardi et al., 2022 [127]	• Europe<br><br>• High Income Country	• Prevalence/Incidence<br><br>• Physical violence<br>• Sexual violence<br>• Emotional/Psychological	• Funding not specified/Unclear	• Quantitative	• Secondary data analysis	• Not specified/unclear	• Survivors<br>• Perpetrators<br><br>• Age group(s) not specified/ Unclear
S129	Fogarty et al., 2022 [128]	• Western Pacific<br><br>• High Income Country	• Experience of violence<br>• Interventions/services with Survivors to address violence<br><br>• Other	• Funded	• Qualitative	• Primary data collection	• Online<br>• Telephone	• Survivors<br>• Practitioner/ Policymaker<br><br>• Age 15 - 24<br>• Age 25 - 50
S130	Folayan et al., 2022 [129]	• Africa<br><br>• Lower/ Middle Income Country	• Experience of violence<br><br>• Physical violence<br>• Sexual violence<br>• Emotional/Psychological<br>• Coercive control	• Funded	• Quantitative	• Primary data collection	• Online	• Survivors<br><br>• Mixed Age Groups

S131	Forry et al., 2022 [130]	• Africa<br><br>• Lower/ Middle Income Country	• Prevalence/Incidence<br>• Experience of violence<br><br>• Physical violence<br>• Sexual violence<br>• Emotional/Psychological<br>• Forced marriage<br>• Coercive control<br>• Other	• Funding not specified/Unclear	• Quantitative	• Primary data collection	• Face to face	• Survivors<br><br>• Mixed Age Groups
S132	Gama et al., 2020 [131]	• Europe<br><br>• High Income Country	• Prevalence/Incidence<br><br>• Physical violence<br>• Sexual violence<br>• Emotional/Psychological	• Funded	• Quantitative	• Primary data collection	• Online	• Survivors<br><br>• Mixed Age Groups
S133	García-Fernández et al., 2021 [132]	• Europe<br><br>• High Income Country	• Experience of violence<br><br>• Emotional/Psychological	• Funding not specified/Unclear	• Quantitative	• Primary data collection	• Online	• Survivors<br><br>• Age 50 +
S134	García-Zamora et al., 2022 [133]	• The Americas<br><br>• Lower/ Middle Income Country<br>• High Income Country	• Prevalence/Incidence<br><br>• Physical violence<br>• Emotional/Psychological	• Not funded	• Quantitative	• Primary data collection	• Online	• Survivors<br><br>• Mixed Age Groups
S135	Gassó et al., 2021 [134]	• Europe<br><br>• High Income Country	• Prevalence/Incidence<br><br>• Sexual violence	• Funded	• Quantitative	• Primary data collection	• Online	• Survivors<br><br>• Mixed Age Groups

S136	Gebrewahd et al., 2020 [135]	• Africa<br><br>• Lower/ Middle Income Country	• Experience of violence<br><br>• Physical violence<br>• Sexual violence<br>• Emotional/Psychological	• Not funded	• Quantitative	• Primary data collection	• Face to face	• Survivors<br><br>• Age 15 - 24<br>• Age 25 - 50
S137	Ghimire et al., 2020[136]	• South-East Asia<br><br>• Lower/ Middle Income Country	• Experience of violence<br><br>• Physical violence<br>• Emotional/Psychological<br>• Other	• Funding not specified/Unclear	• Quantitative	• Primary data collection	• Online	• Survivors<br>• Perpetrators<br><br>• Mixed Age Groups
S138	Gilchrist et al., 2023 [137]	• Europe<br>• The Americas<br>• Western Pacific<br><br>• High Income Country	• Experience of violence<br><br>• Physical violence<br>• Sexual violence<br>• Emotional/Psychological<br>• Coercive control	• Funded	• Quantitative	• Primary data collection	• Online	• Survivors<br>• Perpetrators<br><br>• Mixed Age Groups
S139	Gleason et al., 2021 [138]	• The Americas<br><br>• High Income Country	• Prevalence/Incidence<br><br>• Physical violence<br>• Sexual violence	• Funded	• Quantitative	• Primary data collection	• Online	• Survivors<br><br>• Mixed Age Groups
S140	Glowacz et al., 2022 [139]	• Europe<br><br>• High Income Country	• Prevalence/Incidence<br><br>• Physical violence<br>• Sexual violence<br>• Emotional/Psychological	• Not funded	• Quantitative	• Primary data collection	• Online	• Survivors<br>• Perpetrators<br><br>• Mixed Age Groups
S141	Gonçalves Júnior et al., 2022 [140]	• The Americas<br><br>• Lower/ Middle Income Country	• Prevalence/Incidence<br><br>• Other	• Funding not specified/Unclear	• Quantitative	• Secondary data analysis	• Not specified/unclear	• Survivors<br><br>• Age group(s) not specified/ Unclear

S142	Gosangi et al., 2021 [141]	• The Americas<br><br>• High Income Country	• Prevalence/Incidence<br><br>• Physical violence	• Funding not specified/Unclear	• Quantitative	• Secondary data analysis	• Online	• Survivors<br><br>• Age 25 - 50
S143	Gregory & Williamson, 2022 [142]	• Europe<br><br>• High Income Country	• Other<br><br>• Other	• Funded	• Qualitative	• Secondary data analysis	• Online	• Other<br><br>• Mixed Age Groups
S144	Gulesci et al., 2021 [143]	• Europe<br><br>• High Income Country	• Interventions/services with Survivors to address violence<br><br>• Physical violence<br>• Sexual violence<br>• Emotional/Psychological	• Funded	• Quantitative	• Primary data collection	• Face to face<br>• Telephone	• Survivors<br><br>• Age 15 - 24
S145	Haag et al., 2022 [144]	• The Americas<br><br>• High Income Country	• Experience of violence<br><br>• Physical violence<br>• Sexual violence<br>• Emotional/Psychological<br>• Other	• Funded	• Qualitative	• Primary data collection	• Online	• Survivors<br>• Practitioner/ Policymaker<br><br>• Mixed Age Groups
S146	Haddad et al., 2022 [145]	• Eastern Mediterranean<br><br>• Lower/ Middle Income Country	• Experience of violence<br>• Other<br><br>• Emotional/Psychological	• Not funded	• Quantitative	• Primary data collection	• Online	• Survivors<br><br>• Mixed Age Groups

S147	Halperin et al., 2023 [146]	• Europe<br><br>• High Income Country	• Experience of violence<br><br>• Physical violence<br>• Sexual violence<br>• Emotional/Psychological<br>• Coercive control	• Not funded	• Quantitative	• Primary data collection	• Online	• Survivors<br><br>• Age 15 - 24<br>• Age 25 - 50
S148	Hamadani et al., 2020 [147]	• South-East Asia<br><br>• Lower/ Middle Income Country	• Prevalence/Incidence<br><br>• Physical violence<br>• Sexual violence<br>• Emotional/Psychological	• Funded	• Quantitative	• Primary data collection	• Telephone	• Survivors<br><br>• Age group(s) not specified/ Unclear
S149	Hamamra, 2022[148]	• N/A<br><br>• Lower/ Middle Income Country	• Experience of violence<br><br>• Physical violence<br>• Emotional/Psychological<br>• Coercive control	• Funding not specified/Unclear	• Qualitative	• Primary data collection	• Online	• Survivors<br>• Practitioner/ Policymaker<br><br>• Age 50 +
S150	Hamzaoglu et al., 2023 [149]	• Europe<br><br>• Lower/ Middle Income Country	• Prevalence/Incidence<br><br>• Other	• Not funded	• Quantitative	• Primary data collection	• Online	• Survivors<br><br>• Mixed Age Groups
S151	Haq et al., 2020 [150]	• Eastern Mediterranean<br><br>• Lower/ Middle Income Country	• Experience of violence<br><br>• Emotional/Psychological	• Not funded	• Quantitative	• Primary data collection	• Online	• Survivors<br><br>• Mixed Age Groups

S152	Hassan et al., 2023 [151]	• Africa<br><br>• Lower/ Middle Income Country	• Experience of violence<br><br>• Physical violence<br>• Sexual violence<br>• Femicide<br>• Coercive control<br>• Other	• Funded	• Qualitative	• Primary data collection	• Telephone	• Survivors<br><br>• Age group(s) not specified/ Unclear
S153	Hassoun Ayoub et al., 2023 [152]	• The Americas<br><br>• High Income Country	• Experience of violence<br><br>• Physical violence<br>• Sexual violence	• Funded	• Mixed Methods	• Primary data collection	• Online	• Survivors<br><br>• Age 25 - 50
S154	Heck et al., 2023 [153]	• The Americas<br><br>• High Income Country	• Prevalence/Incidence<br><br>• Other	• Funded	• Quantitative	• Primary data collection	• Online	• Survivors<br>• Perpetrators<br><br>• Mixed Age Groups
S155	Helito et al., 2021 [154]	• The Americas<br><br>• Lower/ Middle Income Country	• Experience of violence<br><br>• Other	• Funded	• Quantitative	• Primary data collection	• Online	• Survivors<br><br>• Age 15 - 24
S156	Heward-Belle et al., 2022[155]	• Western Pacific<br><br>• High Income Country	• Prevalence/Incidence<br><br>• Other	• Funded	• Qualitative	• Primary data collection	• Online	• Practitioner/ Policymaker<br><br>• Age group(s) not specified/ Unclear
S157	Hildersley et al., 2022 [156]	• Europe<br><br>• High Income Country	• Experience of violence<br><br>• Other	• Funded	• Quantitative	• Secondary data analysis	• Online	• Survivors<br><br>• Age 25 - 50

S158	Wong et al., 2022 [157]	• Western Pacific<br><br>• Lower/ Middle Income Country	• Prevalence/Incidence<br><br>• Sexual violence	• Funded	• Quantitative	• Secondary data analysis	• Online	• Survivors<br><br>• Age 15 - 24
S159	Hoehn-Velasco et al., 2021 [158]	• The Americas<br><br>• Lower/ Middle Income Country	• Prevalence/Incidence<br><br>• Physical violence<br>• Sexual violence<br>• Emotional/Psychological<br>• Femicide<br>• Coercive control	• Funding not specified/Unclear	• Quantitative	• Secondary data analysis	• Not specified/unclear	• Survivors<br><br>• Age group(s) not specified/ Unclear
S160	Holland et al., 2021 [159]	• The Americas<br><br>• High Income Country	• Prevalence/Incidence<br><br>• Other	• Funding not specified/Unclear	• Quantitative	• Secondary data analysis	• Online	• Survivors<br><br>• Mixed Age Groups
S161	Hossain et al., 2021 [160]	• South-East Asia<br><br>• Lower/ Middle Income Country	• Prevalence/Incidence<br><br>• Other	• Not funded	• Quantitative	• Primary data collection	• Online	• Survivors<br><br>• Mixed Age Groups
S162	Howard et al., 2022 [161]	• The Americas<br><br>• High Income Country	• Experience of violence<br><br>• Physical violence<br>• Emotional/Psychological	• Funded	• Quantitative	• Primary data collection	• Online	• Survivors<br><br>• Age 15 - 24<br>• Age 25 - 50
S163	Huiskes et al., 2022 [162]	• Europe<br><br>• High Income Country	• Experience of violence<br><br>• Sexual violence<br>• Coercive control	• Funded	• Quantitative	• Primary data collection	• Online	• Survivors<br><br>• Mixed Age Groups

S164	Huq et al., 2021 [163]	• South-East Asia<br><br>• Lower/ Middle Income Country	• Interventions/services with Survivors to address violence<br><br>• Physical violence<br>• Emotional/Psychological<br>• Coercive control<br>• Other	• Funded	• Qualitative	• Primary data collection	• Telephone	• Survivors<br><br>• Mixed Age Groups
S165	Hurley & Morganson, 2023 [164]	• The Americas<br><br>• High Income Country	• Experience of violence<br><br>• Sexual violence	• Funding not specified/Unclear	• Quantitative	• Primary data collection	• Online	• Survivors<br><br>• Mixed Age Groups
S166	Huscsava et al., 2022 [165]	• Europe<br><br>• High Income Country	• Prevalence/Incidence<br>• Interventions/services with Survivors to address violence<br><br>• Physical violence<br>• Sexual violence<br>• Emotional/Psychological	• Funded	• Qualitative	• Primary data collection	• Online	• Survivors<br><br>• Age 15 - 24
S167	Indu et al., 2021 [166]	• South-East Asia<br><br>• Lower/ Middle Income Country	• Prevalence/Incidence<br><br>• Physical violence<br>• Sexual violence<br>• Emotional/Psychological	• Not funded	• Quantitative	• Primary data collection	• Face to face	• Survivors
S168	Iob et al., 2020 [167]	• Europe<br><br>• High Income Country	• Experience of violence<br><br>• Physical violence<br>• Emotional/Psychological	• Funded	• Quantitative	• Primary data collection	• Online	• Survivors<br><br>• Mixed Age Groups

S169	Iverson et al., 2022 [168]	• The Americas<br><br>• High Income Country	• Prevalence/Incidence<br><br>• Physical violence<br>• Sexual violence<br>• Emotional/Psychological	• Funded	• Quantitative	• Primary data collection	• Online	• Survivors<br><br>• Age 50 +
S170	Jiang et al., 2022 [169]	• Western Pacific<br><br>• Lower/ Middle Income Country	• Experience of violence<br><br>• Physical violence<br>• Sexual violence<br>• Emotional/Psychological	• Funded	• Quantitative	• Primary data collection	• Online	• Survivors<br><br>• Mixed Age Groups
S171	Johnson, 2021 [170]	• The Americas<br><br>• High Income Country	• Experience of violence<br><br>• Physical violence<br>• Sexual violence<br>• Emotional/Psychological<br>• Coercive control	• Not funded	• Quantitative	• Primary data collection	• Online	• Survivors
S172	Johnson et al., 2022 [171]	• The Americas<br><br>• High Income Country	• Experience of violence<br><br>• Other	• Funded	• Quantitative	• Primary data collection	• Face to face<br>• Online	• Survivors<br><br>• Age 15 - 24<br>• Age 25 - 50
S173	Johnston et al., 2023 [172]	• N/A	• Prevalence/Incidence<br><br>• Sexual violence<br>• Emotional/Psychological<br>• Coercive control<br>• Other	• Funding not specified/Unclear	• Quantitative	• Primary data collection	• Online	• Practitioner/ Policymaker<br><br>• Age group(s) not specified/ Unclear

S174	Joseph & Canlas, 2023 [173]	• South-East Asia<br><br>• Lower/ Middle Income Country	• Prevalence/Incidence<br><br>• Physical violence<br>• Sexual violence<br>• Emotional/Psychological	• Not funded	• Quantitative	• Primary data collection	• Face to face	• Survivors<br><br>• Age 15 - 24<br>• Age 25 - 50
S175	Ike et al., 2022 [174]	• Africa<br><br>• Lower/ Middle Income Country	• Experience of violence<br><br>• Physical violence<br>• Sexual violence<br>• Emotional/Psychological<br>• Coercive control	• Not funded	• Qualitative	• Primary data collection	• Face to face<br>• Telephone	• Survivors<br><br>• Mixed Age Groups
S176	Jung et al., 2020 [175]	• Europe<br><br>• High Income Country	• Prevalence/Incidence<br><br>• Physical violence<br>• Sexual violence<br>• Emotional/Psychological	• Not funded	• Quantitative	• Primary data collection	• Not specified/unclear	• Survivors<br><br>• Mixed Age Groups
S177	Kachi et al., 2021 [176]	• Western Pacific<br><br>• High Income Country	• Experience of violence<br><br>• Physical violence<br>• Emotional/Psychological<br>• Coercive control	• Funded	• Quantitative	• Primary data collection	• Online	• Survivors<br><br>• Age 15 - 24<br>• Age 25 - 50
S178	Kadra-Scalzo et al., 2023 [177]	• Europe<br><br>• High Income Country	• Experience of violence<br><br>• Physical violence<br>• Emotional/Psychological	• Funded	• Quantitative	• Secondary data analysis	• Online	• Survivors<br><br>• Mixed Age Groups

S179	Kamali et al., 2023 [178]	• Eastern Mediterranean<br><br>• Lower/ Middle Income Country	• Prevalence/Incidence<br><br>• Physical violence<br>• Emotional/Psychological<br>• Coercive control	• Funded	• Quantitative	• Primary data collection	• Telephone	• Survivors<br><br>• Mixed Age Groups
S180	Kamath et al., 2022 [179]	• South-East Asia<br><br>• Lower/ Middle Income Country	• Experience of violence<br><br>• Physical violence<br>• Sexual violence<br>• Emotional/Psychological	• Funding not specified/Unclear	• Quantitative	• Primary data collection	• Face to face	• Survivors<br><br>• Mixed Age Groups
S181	Kamoshida et al., 2022 [180]	• Western Pacific<br><br>• High Income Country	• Prevalence/Incidence<br><br>• Emotional/Psychological<br>• Other	• Not funded	• Quantitative	• Primary data collection	• Online	• Survivors<br><br>• Mixed Age Groups
S182	Karakasi et al., 2023 [181]	• Europe<br><br>• High Income Country	• Prevalence/Incidence<br><br>• Femicide	• Not funded	• Quantitative	• Secondary data analysis	• Not specified/unclear	• Survivors<br><br>• Age group(s) not specified/ Unclear
S183	Karp et al., 2021 [182]	• Africa<br><br>• Lower/ Middle Income Country	• Prevalence/Incidence<br>• Experience of violence<br><br>• Other	• Funded	• Mixed Methods	• Primary data collection	• Telephone	• Survivors<br><br>• Age 15 - 24
S184	Kassie et al., 2022 [183]	• Africa<br><br>• Lower/ Middle Income Country	• Prevalence/Incidence<br><br>• Physical violence<br>• Sexual violence<br>• Emotional/Psychological	• Not funded	• Quantitative	• Primary data collection	• Face to face	• Survivors<br><br>• Mixed Age Groups
S185	Kaswa, 2021 [184]	• Africa<br><br>• Lower/ Middle Income Country	• Prevalence/Incidence<br><br>• Sexual violence	• Not funded	• Quantitative	• Secondary data analysis	• Online	• Survivors<br><br>• Age group(s)

								not specified/ Unclear
S186	Katana et al., 2021 [185]	• Africa<br><br>• Lower/ Middle Income Country	• Experience of violence<br><br>• Physical violence<br>• Emotional/Psychological<br>• Other	• Funded	• Quantitative	• Primary data collection	• Online	• Survivors<br><br>• Mixed Age Groups
S187	Kaur, 2022 [186]	• South-East Asia<br><br>• Lower/ Middle Income Country	• Experience of violence<br><br>• Emotional/Psychological	• Not funded	• Qualitative	• Primary data collection	• Telephone	• Survivors<br><br>• Mixed Age Groups
S188	Kliem et al., 2023 [187]	• Europe<br><br>• High Income Country	• Experience of violence<br><br>• Physical violence<br>• Sexual violence<br>• Emotional/Psychological	• Not funded	• Quantitative	• Primary data collection<br>• Secondary data analysis	• Face to face	• Survivors<br><br>• Mixed Age Groups
S189	Koly et al., 2022 [188]	• South-East Asia<br><br>• Lower/ Middle Income Country	• Experience of violence<br><br>• Physical violence<br>• Sexual violence<br>• Emotional/Psychological	• Not funded	• Qualitative	• Secondary data analysis	• Online	• Survivors
S190	Krause et al., 2023 [189]	• The Americas<br><br>• High Income Country	• Experience of violence<br><br>• Physical violence<br>• Sexual violence<br>• Emotional/Psychological	• Not funded	• Quantitative	• Secondary data analysis	• Online	• Survivors<br><br>• Age 15 - 24

S191	Krause et al., 2022 [190]	• The Americas<br><br>• High Income Country	• Experience of violence<br><br>• Physical violence<br>• Emotional/Psychological	• Funding not specified/Unclear	• Quantitative	• Primary data collection	• Online	• Survivors<br><br>• Age 15 - 24
S192	Krishnamurti et al., 2021 [191]	• The Americas<br><br>• High Income Country	• Experience of violence<br><br>• Physical violence<br>• Sexual violence<br>• Emotional/Psychological	• Not funded	• Quantitative	• Primary data collection	• Online	• Survivors<br><br>• Age group(s) not specified/ Unclear
S193	Kuhlmann et al., 2023 [192]	• Europe<br><br>• High Income Country	• Experience of violence<br><br>• Sexual violence	• Funded	• Quantitative	• Primary data collection	• Online	• Survivors<br><br>• Age 25 - 50<br>• Age 50 +
S194	Kulik & Ramon, 2022 [193]	• Europe<br><br>• High Income Country	• Experience of violence<br><br>• Physical violence<br>• Emotional/Psychological	• Funded	• Quantitative	• Primary data collection	• Online	• Survivors<br><br>• Mixed Age Groups
S195	Kulik, 2023 [194]	• Europe<br><br>• High Income Country	• Prevalence/Incidence<br><br>• Physical violence<br>• Emotional/Psychological<br>• Other	• Funding not specified/Unclear	• Quantitative	• Primary data collection	• Online	• Survivors<br><br>• Mixed Age Groups
S196	Lafta et al., 2021 [195]	• Eastern Mediterranean<br><br>• Lower/ Middle Income Country	• Experience of violence<br><br>• Physical violence<br>• Emotional/Psychological	• Not funded	• Quantitative	• Primary data collection	• Face to face	• Survivors<br><br>• Mixed Age Groups

S197	Lamichhane et al., 2021 [196]	• South-East Asia<br><br>• Lower/ Middle Income Country	• Prevalence/Incidence<br>• Experience of violence<br><br>• Physical violence<br>• Sexual violence	• Funded	• Quantitative	• Primary data collection	• Telephone	• Survivors<br><br>• Age 15 - 24
S198	Lampe et al., 2021 [197]	• Europe<br><br>• High Income Country	• Prevalence/Incidence<br><br>• Physical violence<br>• Other	• Funding not specified/Unclear	• Qualitative	• Primary data collection	• Telephone	• Survivors<br><br>• Mixed Age Groups
S199	Langhinrichsen-Rohling et al., 2022 [198]	• The Americas<br><br>• High Income Country	• Prevalence/Incidence<br><br>• Physical violence	• Funded	• Quantitative	• Primary data collection	• Online	• Survivors<br>• Perpetrators<br><br>• Age 25 - 50
S200	Lazzerini et al., 2022 [199]	• Europe<br><br>• Lower/ Middle Income Country<br>• High Income Country	• Experience of violence<br><br>• Physical violence<br>• Emotional/Psychological	• Funded	• Quantitative	• Primary data collection	• Online	• Survivors<br><br>• Age 15 - 24<br>• Age 25 - 50
S201	Lee et al., 2021 [200]	• The Americas<br><br>• Lower/ Middle Income Country	• Prevalence/Incidence<br><br>• Other	• Funded	• Quantitative	• Secondary data analysis	• Not specified/unclear	• Survivors<br><br>• Mixed Age Groups
S202	Leikuma-Rimicane et al., 2022 [201]	• Western Pacific<br><br>• Lower/ Middle Income Country	• Prevalence/Incidence<br><br>• Physical violence<br>• Sexual violence<br>• Femicide	• Funding not specified/Unclear	• Quantitative	• Secondary data analysis	• Not specified/unclear	• Survivors<br><br>• Age group(s) not specified/ Unclear

S203	Leon-Larios et al., 2022 [202]	• Europe<br><br>• High Income Country	• Prevalence/Incidence<br><br>• Other	• Funded	• Quantitative	• Primary data collection	• Telephone	• Survivors<br><br>• Mixed Age Groups
S204	Leske et al., 2021 [203]	• Western Pacific<br><br>• High Income Country	• Prevalence/Incidence<br><br>• Other	• Funded	• Quantitative	• Secondary data analysis	• Not specified/unclear	• Survivors<br><br>• Age group(s) not specified/ Unclear
S205	Lipp & Johnson, 2023 [204]	• The Americas<br><br>• High Income Country	• Prevalence/Incidence<br><br>• Other	• Funded	• Mixed Methods	• Secondary data analysis	• Online<br>• Not specified/unclear	• Practitioner/ Policymaker<br><br>• Age group(s) not specified/ Unclear
S206	Liu et al., 2021 [205]	• Western Pacific<br><br>• Lower/ Middle Income Country	• Prevalence/Incidence<br><br>• Physical violence<br>• Emotional/Psychological	• Funded	• Quantitative	• Primary data collection	• Online	• Survivors<br><br>• Mixed Age Groups
S207	Logie et al., 2021 [206]	• Africa<br><br>• Lower/ Middle Income Country	• Experience of violence<br><br>• Sexual violence<br>• Forced marriage<br>• Other	• Funded	• Qualitative	• Primary data collection	• Not specified/unclear	• Survivors<br><br>• Age 15 - 24
S208	Long et al., 2022 [207]	• Western Pacific<br><br>• Lower/ Middle Income Country	• Prevalence/Incidence<br><br>• Physical violence<br>• Sexual violence<br>• Emotional/Psychological	• Funded	• Quantitative	• Primary data collection	• Online	• Survivors<br><br>• Age 15 - 24

S209	Luebke et al., 2023 [208]	• The Americas<br><br>• High Income Country	• Experience of violence<br><br>• Physical violence<br>• Sexual violence<br>• Emotional/Psychological<br>• Other	• Funded	• Qualitative	• Primary data collection	• Face to face<br>• Online<br>• Telephone	• Survivors<br><br>• Mixed Age Groups
S210	Lyons & Brewer, 2022 [209]	• N/A	• Experience of violence<br><br>• Other	• Funding not specified/Unclear	• Qualitative	• Secondary data analysis	• Online	• Survivors<br><br>• Age group(s) not specified/ Unclear
S211	MacGregor et al., 2023 [210]	• The Americas<br><br>• High Income Country	• Interventions/services with Survivors to address violence<br><br>• Emotional/Psychological	• Funded	• Quantitative	• Primary data collection	• Online	• Survivors<br><br>• Age group(s) not specified/ Unclear
S212	Maftei & Dănilă, 2023 [211]	• Europe<br><br>• Lower/ Middle Income Country	• Prevalence/Incidence<br><br>• Emotional/Psychological<br>• Coercive control	• Funding not specified/Unclear	• Quantitative	• Primary data collection	• Online	• Survivors<br>• Perpetrators<br><br>• Mixed Age Groups
S213	Mahamid et al., 2022 [212]	• N/A<br><br>• Lower/ Middle Income Country	• Prevalence/Incidence<br>• Experience of violence<br><br>• Physical violence<br>• Emotional/Psychological<br>• Other	• Not funded	• Qualitative	• Primary data collection	• Face to face	• Practitioner/ Policymaker<br><br>• Age 15 - 24<br>• Age 25 - 50
S214	Mahapatro et al., 2021 [213]	• South-East Asia<br><br>• Lower/ Middle Income Country	• Interventions/services with Survivors to address violence<br><br>• Other	• Not funded	• Qualitative	• Primary data collection	• Telephone	• Survivors<br><br>• Age group(s) not specified/ Unclear

S215	Mahapatro et al., 2023 [214]	• South-East Asia<br><br>• Lower/ Middle Income Country	• Prevalence/Incidence<br><br>• Physical violence<br>• Sexual violence<br>• Emotional/Psychological	• Funding not specified/Unclear	• Quantitative	• Secondary data analysis	• Not specified/unclear	• Survivors<br><br>• Mixed Age Groups
S216	Maharlouei et al., 2023 [215]	• Eastern Mediterranean<br><br>• Lower/ Middle Income Country	• Experience of violence<br><br>• Physical violence<br>• Sexual violence<br>• Emotional/Psychological	• Funded	• Quantitative	• Primary data collection	• Face to face<br>• Online	• Survivors<br><br>• Age 15 - 24<br>• Age 25 - 50
S217	Mahlangu et al., 2022 [216]	• Africa<br><br>• Lower/ Middle Income Country	• Experience of violence<br><br>• Physical violence<br>• Emotional/Psychological	• Funded	• Qualitative	• Primary data collection	• Online	• Survivors<br><br>• Age 25 - 50<br>• Age 50 +
S218	Mahmood et al., 2022 [217]	• Eastern Mediterranean<br><br>• Lower/ Middle Income Country	• Prevalence/Incidence<br><br>• Physical violence<br>• Sexual violence<br>• Emotional/Psychological	• Not funded	• Quantitative	• Primary data collection	• Online	• Survivors<br><br>• Mixed Age Groups
S219	Mahmud & Riley, 2021 [218]	• Africa<br><br>• Lower/ Middle Income Country	• Prevalence/Incidence *prevalence and perception*<br><br>• Other	• Funded	• Quantitative	• Primary data collection	• Face to face<br>• Telephone	• Not specified/ Unclear<br><br>• Age group(s) not specified/ Unclear
S220	Maji et al., 2022 [219]	• South-East Asia<br><br>• Lower/ Middle Income Country	• Prevalence/Incidence<br><br>• Other	• Funding not specified/Unclear	• Quantitative	• Secondary data analysis	• Online	• Not specified/ Unclear<br><br>• Age group(s) not specified/ Unclear

S221	Malkawi et al., 2021 [220]	• Eastern Mediterranean<br><br>• Lower/ Middle Income Country	• Prevalence/Incidence<br><br>• Other	• Funding not specified/Unclear	• Quantitative	• Primary data collection	• Online	• Survivors<br><br>• Mixed Age Groups
S222	Mantell et al., 2021 [221]	• Africa<br><br>• Lower/ Middle Income Country	• Prevalence/Incidence<br><br>• Other	• Funded	• Quantitative	• Primary data collection	• Telephone	• Survivors<br><br>• Age 15 - 24
S223	Mantler, Shillington, Yates, et al., 2022 [222]	• The Americas<br><br>• High Income Country	• Experience of violence<br>• Interventions/services with Survivors to address violence<br><br>• Other	• Funded	• Qualitative	• Primary data collection	• Online<br>• Telephone	• Survivors<br>• Practitioner/ Policymaker<br><br>• Mixed Age Groups
S224	Mantler, Shillington, Davidson, et al., 2022 [223]	• The Americas<br><br>• High Income Country	• Experience of violence<br><br>• Physical violence<br>• Sexual violence<br>• Emotional/Psychological	• Funded	• Mixed Methods	• Primary data collection	• Online	• Survivors<br><br>• Mixed Age Groups
S225	Manzoor et al., 2022 [224]	• Eastern Mediterranean<br>• Europe<br>• South-East Asia<br><br>• Lower/ Middle Income Country<br>• High Income Country	• Experience of violence<br><br>• Physical violence<br>• Sexual violence<br>• Acid attacks<br>• Forced marriage<br>• Other	• Funded	• Quantitative	• Secondary data analysis	• Online	• Survivors<br><br>• Age group(s) not specified/ Unclear

S226	Marchant et al., 2021 [225]	• The Americas<br><br>• High Income Country	• Prevalence/Incidence<br><br>• Physical violence	• Funded	• Quantitative	• Secondary data analysis	• Face to face	• Survivors<br><br>• Mixed Age Groups
S227	Marsh et al., 2022 [226]	• The Americas<br><br>• High Income Country	• Prevalence/Incidence<br><br>• Physical violence<br>• Emotional/Psychological	• Not funded	• Quantitative	• Secondary data analysis	• Online	• Survivors<br><br>• Age group(s) not specified/ Unclear
S228	Matose et al., 2022 [227]	• Africa<br><br>• Lower/ Middle Income Country	• Experience of violence<br><br>• Physical violence<br>• Sexual violence	• Not funded	• Qualitative	• Primary data collection	• Face to face	• Survivors<br><br>• Mixed Age Groups
S229	McCool-Myers, Kozlowski, et al., 2022 [228]	• The Americas<br><br>• High Income Country	• Experience of violence<br><br>• Coercive control<br>• Other	• Funded	• Quantitative	• Primary data collection	• Online	• Survivors<br><br>• Age 15 - 24<br>• Age 25 - 50
S230	McCool-Myers, Grasso, et al., 2022 [229]	• Europe<br><br>• Lower/ Middle Income Country	• Experience of violence<br><br>• Other	• Funded	• Quantitative	• Primary data collection	• Online	• Survivors<br><br>• Age 15 - 24<br>• Age 25 - 50
S231	McDougal et al., 2022 [230]	• The Americas<br><br>• High Income Country	• Experience of violence<br><br>• Other	• Funded	• Quantitative	• Primary data collection	• Face to face	• Survivors<br><br>• Mixed Age Groups
S232	McKinlay et al., 2023 [231]	• Europe<br><br>• High Income Country	• Experience of violence<br><br>• Physical violence<br>• Emotional/Psychological<br>• Coercive control	• Funded	• Qualitative	• Primary data collection	• Online	• Survivors<br><br>• Mixed Age Groups

S233	McMillan et al., 2021 [232]	• The Americas<br><br>• High Income Country	• Prevalence/Incidence<br><br>• Other	• Not funded	• Quantitative	• Primary data collection	• Online	• Survivors<br><br>• Age 15 - 24<br>• Age 25 - 50
S234	Abu-Rmeileh et al., 2022 [233]	• N/A<br><br>• Lower/ Middle Income Country	• Prevalence/Incidence<br><br>• Physical violence<br>• Emotional/Psychological<br>• Other	• Funded	• Quantitative	• Primary data collection	• Telephone	• Survivors<br><br>• Age 15 - 24<br>• Age 25 - 50
S235	Melaku & Beeman, 2023 [234]	• The Americas<br><br>• High Income Country	• Experience of violence<br><br>• Other	• Funding not specified/Unclear	• Qualitative	• Primary data collection	• Online	• Survivors<br><br>• Mixed Age Groups
S236	Mena-Tudela et al., 2022 [235]	• Europe<br><br>• High Income Country	• Experience of violence<br><br>• Other	• Not funded	• Quantitative	• Primary data collection	• Online	• Survivors<br><br>• Age 15 - 24<br>• Age 25 - 50
S237	Merken et al., 2023 [236]	• The Americas<br><br>• High Income Country	• Prevalence/Incidence<br>• Experience of violence<br><br>• Physical violence<br>• Emotional/Psychological<br>• Other	• Funding not specified/Unclear	• Qualitative	• Primary data collection	• Online	• Not specified/ Unclear<br><br>• Mixed Age Groups
S238	Miller et al., 2022 [237]	• Africa<br><br>• Lower/ Middle Income Country	• Prevalence/Incidence<br><br>• Physical violence<br>• Sexual violence<br>• Emotional/Psychological	• Funded	• Quantitative	• Primary data collection	• Telephone	• Survivors<br><br>• Mixed Age Groups

S239	Mingude & Dejene, 2021 [238]	• Africa<br><br>• Lower/ Middle Income Country	• Prevalence/Incidence<br>• Experience of violence<br><br>• Physical violence<br>• Sexual violence<br>• Other	• Not funded	• Quantitative	• Primary data collection	• Face to face	• Survivors<br><br>• Age 15 - 24
S240	Moawad et al., 2021 [239]	• Eastern Mediterranean<br><br>• Lower/ Middle Income Country	• Prevalence/Incidence<br><br>• Physical violence<br>• Sexual violence<br>• Emotional/Psychological	• Not funded	• Quantitative	• Primary data collection	• Online	• Survivors<br><br>• Mixed Age Groups
S241	Moore et al., 2022 [240]	• Europe<br><br>• High Income Country	• Experience of violence<br><br>• Other	• Funded	• Quantitative	• Secondary data analysis	• Not specified/unclear	• Survivors<br><br>• Mixed Age Groups
S242	Morales Chainé et al., 2021 [241]	• The Americas<br><br>• Lower/ Middle Income Country	• Experience of violence<br><br>• Physical violence<br>• Emotional/Psychological	• Funded	• Quantitative	• Primary data collection	• Online	• Survivors<br><br>• Mixed Age Groups
S243	Morales Chainé et al., 2022 [242]	• The Americas<br><br>• Lower/ Middle Income Country	• Prevalence/Incidence<br>• Experience of violence<br><br>• Physical violence<br>• Sexual violence<br>• Emotional/Psychological	• Funded	• Quantitative	• Primary data collection	• Online	• Survivors<br><br>• Mixed Age Groups
S244	Morgan et al., 2022 [243]	• Western Pacific<br><br>• High Income Country	• Prevalence/Incidence<br><br>• Physical violence<br>• Sexual violence	• Not funded	• Quantitative	• Primary data collection	• Online	• Survivors<br><br>• Mixed Age Groups

S245	Morley et al., 2021 [244]	• Western Pacific<br><br>• High Income Country	• Prevalence/Incidence<br>• Experience of violence<br><br>• Physical violence<br>• Emotional/Psychological<br>• Coercive control<br>• Other	• Funded	• Qualitative	• Primary data collection	• Online	• Survivors<br>• Not specified/ Unclear<br><br>• Age group(s) not specified/ Unclear
S246	Moro et al., 2022 [245]	• Europe<br><br>• High Income Country	• Experience of violence<br><br>• Other	• Funded	• Quantitative	• Primary data collection	• Online	• Survivors<br><br>• Mixed Age Groups
S247	Moss & Mahmoudi, 2021 [246]		• Experience of violence<br><br>• Emotional/Psychological	• Not funded	• Quantitative	• Primary data collection	• Online	• Survivors<br><br>• Age group(s) not specified/ Unclear
S248	Moulin et al., 2023 [247]	• Europe<br><br>• High Income Country	• Experience of violence<br><br>• Physical violence	• Not funded	• Quantitative	• Primary data collection	• Online	• Survivors<br><br>• Mixed Age Groups
S249	Mubaiwa et al., 2022 [248]	• Africa<br><br>• Lower/ Middle Income Country	• Prevalence/Incidence<br><br>• Other	• Funded	• Qualitative	• Primary data collection	• Not specified/unclear	• Practitioner/ Policymaker<br><br>• Age group(s) not specified/ Unclear
S250	Muldoon, Denize, Talarico, Fell, et al., 2021 [249]	• The Americas<br><br>• High Income Country	• Prevalence/Incidence<br><br>• Physical violence<br>• Sexual violence	• Funded	• Quantitative	• Secondary data analysis	• Not specified/unclear	• Survivors<br><br>• Mixed Age Groups
S251	Muldoon, Denize, Talarico, Boisvert, et al., 2021 [250]	• The Americas	• Experience of violence<br><br>• Sexual violence	• Funded	• Quantitative	• Primary data collection	• Online	• Survivors<br><br>• Age 25 - 50

		• High Income Country	• Coercive control<br>• Other					
S252	Muldoon et al., 2022 [251]	• The Americas<br><br>• High Income Country	• Prevalence/Incidence<br><br>• Sexual violence	• Funded	• Quantitative	• Secondary data analysis	• Not specified/unclear	• Survivors<br><br>• Mixed Age Groups
S253	Muñoz del Carpio-Toia et al., 2021 [252]	• The Americas<br><br>• Lower/ Middle Income Country	• Prevalence/Incidence<br><br>• Physical violence<br>• Sexual violence<br>• Emotional/Psychological	• Funding not specified/Unclear	• Quantitative	• Primary data collection	• Online	• Survivors<br><br>• Mixed Age Groups
S254	Musse et al., 2022 [253]	• The Americas<br><br>• Lower/ Middle Income Country	• Prevalence/Incidence<br><br>• Physical violence<br>• Sexual violence<br>• Emotional/Psychological	• Funding not specified/Unclear	• Quantitative	• Primary data collection	• Online	• Survivors<br><br>• Mixed Age Groups
S255	Mutambara et al., 2022 [254]	• Africa<br><br>• Lower/ Middle Income Country	• Experience of violence<br><br>• Physical violence<br>• Emotional/Psychological	• Funding not specified/Unclear	• Qualitative	• Primary data collection	• Telephone	• Survivors<br><br>• Age 15 - 24<br>• Age 25 - 50
S256	Muthulingam et al., 2022 [255]	• South-East Asia<br><br>• Lower/ Middle Income Country	• Prevalence/Incidence<br><br>• Physical violence<br>• Sexual violence<br>• Emotional/Psychological<br>• Coercive control<br>• Other	• Funding not specified/Unclear	• Quantitative	• Secondary data analysis	• Not specified/unclear	• Survivors<br>• Perpetrators<br><br>• Mixed Age Groups

S257	Nagashima-Hayashi et al., 2022 [256]	• Eastern Mediterranean<br>• South-East Asia<br>• Western Pacific<br><br>• Lower/ Middle Income Country	• Prevalence/Incidence<br><br>• Other	• Funded	• Qualitative	• Primary data collection	• Online<br>• Telephone	• Practitioner/ Policymaker<br><br>• Age group(s) not specified/ Unclear
S258	Nagaswami & Yeung, 2023 [257]	• The Americas<br><br>• High Income Country	• Experience of violence<br><br>• Physical violence<br>• Emotional/Psychological<br>• Coercive control	• Funding not specified/Unclear	• Quantitative	• Primary data collection	• Online	• Survivors<br><br>• Mixed Age Groups
S259	Naghizadeh et al., 2021 [258]	• Eastern Mediterranean<br><br>• Lower/ Middle Income Country	• Experience of violence<br><br>• Physical violence<br>• Sexual violence<br>• Emotional/Psychological	• Funded	• Quantitative	• Primary data collection	• Face to face	• Survivors<br><br>• Age 15 - 24<br>• Age 25 - 50
S260	Napier-Raman et al., 2021 [259]	• South-East Asia<br><br>• Lower/ Middle Income Country	• Experience of violence<br><br>• Physical violence<br>• Emotional/Psychological<br>• Coercive control<br>• Other	• Not funded	• Mixed Methods	• Primary data collection	• Face to face<br>• Telephone	• Survivors<br><br>• Age 15 - 24<br>• Age 25 - 50
S261	Nardi-Rodríguez & Paredes-López, 2022 [260]	• Europe<br><br>• Lower/ Middle Income Country	• Experience of violence<br><br>• Other	• Not funded	• Quantitative	• Primary data collection	• Online	• Not specified/ Unclear<br><br>• Mixed Age Groups
S262	Nerobkova et al., 2022 [261]	• Western Pacific<br><br>• High Income Country	• Prevalence/Incidence<br>• Experience of violence<br><br>• Physical violence<br>• Sexual violence	• Not funded	• Quantitative	• Primary data collection	• Online	• Survivors<br><br>• Mixed Age Groups

S263	Nesset et al., 2021 [262]	• Europe<br><br>• High Income Country	• Prevalence/Incidence<br><br>• Other	• Funded	• Quantitative	• Secondary data analysis	• Not specified/unclear	• Survivors<br>• Perpetrators<br><br>• Mixed Age Groups
S264	Neufeld et al., 2022 [263]	• The Americas<br><br>• High Income Country	• Prevalence/Incidence<br><br>• Physical violence	• Funded	• Quantitative	• Secondary data analysis	• Online	• Survivors<br><br>• Mixed Age Groups
S265	Nhongo et al., 2022 [264]	• Western Pacific<br><br>• High Income Country	• Prevalence/Incidence<br><br>• Physical violence<br>• Other	• Funded	• Quantitative	• Secondary data analysis	• Online	• Survivors<br><br>• Mixed Age Groups
S266	Niederkrotenthaler et al., 2022 [265]	• Europe<br><br>• High Income Country	• Prevalence/Incidence<br><br>• Other	• Funded	• Quantitative	• Primary data collection	• Online	• Survivors<br><br>• Mixed Age Groups
S267	Nishat et al., 2023 [266]	• South-East Asia<br><br>• Lower/ Middle Income Country	• Prevalence/Incidence<br><br>• Physical violence<br>• Sexual violence<br>• Emotional/Psychological<br>• Other	• Funded	• Quantitative	• Primary data collection	• Face to face	• Survivors<br><br>• Age 15 - 24
S268	Nittari et al., 2021 [267]	• Europe<br><br>• High Income Country	• Prevalence/Incidence<br><br>• Physical violence	• Not funded	• Quantitative	• Secondary data analysis	• Online	• Survivors<br><br>• Age 50 +
S269	Nyashanu et al., 2022 [268]	• Africa<br><br>• Lower/ Middle Income Country	• Experience of violence<br><br>• Physical violence<br>• Emotional/Psychological<br>• Other	• Funding not specified/Unclear	• Qualitative	• Primary data collection	• Online	• Survivors<br><br>• Mixed Age Groups

S270	Ogunlana et al., 2021 [269]	• Africa<br><br>• Lower/ Middle Income Country	• Experience of violence<br><br>• Sexual violence<br>• Femicide	• Funding not specified/Unclear	• Mixed Methods	• Secondary data analysis	• Online	• Survivors<br>• Perpetrators<br><br>• Mixed Age Groups
S271	O'Hara & Tan, 2022 [270]	• Western Pacific<br><br>• High Income Country	• Experience of violence<br><br>• Physical violence<br>• Sexual violence<br>• Coercive control	• Funding not specified/Unclear	• Quantitative	• Primary data collection	• Online	• Survivors<br><br>• Mixed Age Groups
S272	Ohliger et al., 2020 [271]	• The Americas<br><br>• High Income Country	• Prevalence/Incidence<br><br>• Other	• Not funded	• Quantitative	• Secondary data analysis	• Online	• Survivors<br><br>• Mixed Age Groups
S273	Okunola et al., 2022[272]	• Africa<br><br>• Lower/ Middle Income Country	• Experience of violence<br><br>• Physical violence<br>• Sexual violence	• Not funded	• Quantitative	• Secondary data analysis	• Not specified/unclear	• Survivors<br><br>• Age 15 - 24<br>• Age 25 - 50
S274	Olding et al., 2021 [273]	• Europe<br><br>• High Income Country	• Prevalence/Incidence<br><br>• Other	• Not funded	• Quantitative	• Secondary data analysis	• Not specified/unclear	• Survivors<br><br>• Mixed Age Groups
S275	Oloniniyi et al., 2023 [274]	• Africa<br><br>• Lower/ Middle Income Country	• Prevalence/Incidence<br><br>• Physical violence<br>• Sexual violence<br>• Emotional/Psychological<br>• Other	• Funding not specified/Unclear	• Quantitative	• Primary data collection	• Online	• Survivors<br><br>• Mixed Age Groups
S276	Oswald et al., 2023 [275]	• The Americas<br><br>• High Income Country	• Prevalence/Incidence<br>• Experience of violence<br><br>• Physical violence<br>• Sexual violence<br>• Emotional/Psychological	• Funding not specified/Unclear	• Quantitative	• Primary data collection	• Online	• Survivors<br><br>• Mixed Age Groups

S277	Ottosson et al., 2022 [276]	• Africa<br><br>• Lower/ Middle Income Country	• Interventions/services with Survivors to address violence<br><br>• Other	• Funded	• Quantitative	• Primary data collection	• Face to face<br>• Online<br>• Telephone	• Survivors<br><br>• Age 15 - 24
S278	Owusu-Addo et al., 2023 [277]	• Africa<br><br>• Lower/ Middle Income Country	• Prevalence/Incidence<br><br>• Sexual violence	• Funded	• Mixed Methods	• Primary data collection	• Face to face	• Survivors<br><br>• Age 15 - 24
S279	Özdamar Ünal et al., 2022 [278]	• Europe<br><br>• Lower/ Middle Income Country	• Prevalence/Incidence<br><br>• Physical violence<br>• Emotional/Psychological	• Not funded	• Quantitative	• Primary data collection	• Online	• Survivors<br><br>• Age 25 - 50
S280	Özümerzifon et al., 2022 [279]	• The Americas<br><br>• High Income Country	• Interventions/services with survivors to address violence<br><br>• Other	• Funded	• Mixed Methods	• Primary data collection	• Online	• Survivors<br><br>• Age 25 - 50
S281	Pakenham et al., 2020 [280]	• Europe<br><br>• High Income Country	• Prevalence/Incidence<br><br>• Other	• Funding not specified/Unclear	• Quantitative	• Primary data collection	• Online	• Survivors<br><br>• Mixed Age Groups
S282	Pallansch et al., 2022 [281]	• The Americas<br><br>• High Income Country	• Prevalence/Incidence<br><br>• Sexual violence<br>• Other	• Funding not specified/Unclear	• Quantitative	• Secondary data analysis	• Not specified/unclear	• Survivors<br><br>• Mixed Age Groups
S283	Pande et al., 2022 [282]	• South-East Asia<br><br>• Lower/ Middle Income Country	• Prevalence/Incidence<br><br>• Physical violence	• Not funded	• Quantitative	• Secondary data analysis	• Not specified/unclear	• Age group(s) not specified/ Unclear

S284	Panovska-Griffiths et al., 2022 [283]	• Europe<br><br>• High Income Country	• Prevalence/Incidence<br><br>• Other	• Funded	• Quantitative	• Secondary data analysis	• Not specified/unclear	• Survivors<br><br>• Age group(s) not specified/ Unclear
S285	Paramasivan et al., 2022 [284]	• South-East Asia<br><br>• Lower/ Middle Income Country	• Experience of violence<br><br>• Physical violence<br>• Sexual violence	• Funded	• Quantitative	• Secondary data analysis	• Telephone<br>• Not specified/unclear	• Survivors<br><br>• Age group(s) not specified/ Unclear
S286	Pattojoshi et al., 2021 [285]	• South-East Asia<br><br>• Lower/ Middle Income Country	• Prevalence/Incidence<br>• Experience of violence<br><br>• Physical violence<br>• Sexual violence<br>• Emotional/Psychological	• Not funded	• Quantitative	• Primary data collection	• Online	• Survivors<br><br>• Mixed Age Groups
S287	Peitzmeier et al., 2022 [286]	• The Americas<br><br>• High Income Country	• Prevalence/Incidence<br><br>• Physical violence<br>• Sexual violence<br>• Emotional/Psychological<br>• Other	• Funded	• Quantitative	• Primary data collection	• Online	• Survivors<br><br>• Mixed Age Groups
S288	Peraud et al., 2021 [287]	• Europe<br><br>• High Income Country	• Experience of violence<br><br>• Physical violence<br>• Sexual violence	• Not funded	• Quantitative	• Primary data collection	• Online	• Survivors<br><br>• Mixed Age Groups
S289	Peters et al., 2021 [288]	• The Americas<br><br>• High Income Country	• Interventions/services with Survivors to address violence<br><br>• Other	• Funded	• Quantitative	• Primary data collection	• Online	• Practitioner/ Policymaker<br><br>• Age 25 - 50

S290	Pfitzner, Fitz-Gibbon, et al., 2022 [289]	• Western Pacific<br><br>• High Income Country	• Experience of violence<br><br>• Other	• Funding not specified/Unclear	• Qualitative	• Primary data collection	• Online	• Practitioner/ Policymaker<br><br>• Mixed Age Groups
S291	Pfitzner, Fitz-Gibbon, et al., 2022 [290]	• Western Pacific<br><br>• High Income Country	• Experience of violence<br><br>• Coercive control<br>• Other	• Funding not specified/Unclear	• Mixed Methods	• Primary data collection	• Online	• Practitioner/ Policymaker<br><br>• Age group(s) not specified/ Unclear
S292	Phillimore et al., 2022 [291]	• Eastern Mediterranean<br>• Europe<br>• Western Pacific<br><br>• Lower/ Middle Income Country<br>• High Income Country	• Prevalence/Incidence<br><br>• Sexual violence<br>• Emotional/Psychological<br>• Other	• Funded	• Qualitative	• Primary data collection	• Online	• Survivors<br><br>• Mixed Age Groups
S293	Pinchoff et al., 2021 [292]	• Africa<br><br>• Lower/ Middle Income Country	• Prevalence/Incidence<br>• Experience of violence<br><br>• Other	• Funded	• Quantitative	• Primary data collection	• Online	• Survivors<br><br>• Mixed Age Groups
S294	Pincock et al., 2022 [293]	• Africa<br>• South-East Asia<br><br>• Lower/ Middle Income Country	• Experience of violence<br><br>• Physical violence<br>• Sexual violence<br>• Emotional/Psychological<br>• Other	• Funded	• Qualitative	• Primary data collection	• Telephone	• Survivors<br>• Practitioner/ Policymaker<br><br>• Age 15 - 24<br>• Age group(s) not specified/ Unclear
S295	Pino et al., 2022 [294]	• The Americas	• Prevalence/Incidence<br><br>• Physical violence	• Funded	• Quantitative	• Secondary data analysis	• Online	• Survivors

		• High Income Country						• Mixed Age Groups
S296	Pisl et al., 2023 [295]	• Europe<br>• High Income Country	• Prevalence/Incidence<br>• Physical violence	• Funded	• Quantitative	• Secondary data analysis	• Not specified/unclear	• Survivors<br>• Mixed Age Groups
S297	Plášilová et al., 2021 [296]	• Europe<br>• High Income Country	• Prevalence/Incidence<br>• Physical violence<br>• Sexual violence<br>• Emotional/Psychological<br>• Other	• Funded	• Quantitative	• Primary data collection	• Online	• Survivors<br>• Mixed Age Groups
S298	Porter et al., 2021 [297]	• The Americas<br>• Lower/ Middle Income Country	• Prevalence/Incidence<br>• Physical violence	• Funded	• Quantitative	• Primary data collection	• Telephone	• Survivors<br>• Mixed Age Groups
S299	Poraz Findik et al., 2023 [298]	• Europe<br>• Lower/ Middle Income Country	• Prevalence/Incidence<br>• Other	• Not funded	• Quantitative	• Secondary data analysis	• Online	• Survivors<br>• Age 15 - 24
S300	Preis et al., 2021 [299]	• The Americas<br>• High Income Country	• Experience of violence<br>• Emotional/Psychological<br>• Other	• Funded	• Quantitative	• Primary data collection	• Online	• Survivors<br>• Age 15 - 24<br>• Age 25 - 50
S301	Rahman et al., 2023 [300]	• South-East Asia<br>• Lower/ Middle Income Country	• Prevalence/Incidence<br>• Physical violence<br>• Emotional/Psychological	• Funding not specified/Unclear	• Quantitative	• Primary data collection	• Online	• Survivors<br>• Mixed Age Groups
S302	Raj et al., 2020 [301]	• The Americas<br>• High Income Country	• Experience of violence<br>• Sexual violence<br>• Other	• Funded	• Quantitative	• Primary data collection	• Online	• Survivors<br>• Mixed Age Groups

S303	Ramirez & Harris, 2022 [302]	• The Americas<br><br>• High Income Country	• Interventions/services with Survivors to address violence<br><br>• Sexual violence<br>• Other	• Funding not specified/Unclear	• Qualitative	• Primary data collection	• Online<br>• Telephone	• Practitioner/ Policymaker<br><br>• Age 25 - 50<br>• Age 50 +
S304	Ranzani et al., 2023 [303]	• The Americas<br><br>• Lower/ Middle Income Country	• Experience of violence<br><br>• Physical violence<br>• Sexual violence<br>• Emotional/Psychological<br>• Coercive control	• Funding not specified/Unclear	• Quantitative	• Primary data collection	• Online	• Survivors<br><br>• Age 50 +
S305	Rashidi Fakari et al., 2022 [304]	• Eastern Mediterranean<br><br>• Lower/ Middle Income Country	• Experience of violence<br><br>• Physical violence<br>• Sexual violence<br>• Emotional/Psychological	• Funding not specified/Unclear	• Quantitative	• Primary data collection	• Online	• Survivors<br><br>• Mixed Age Groups
S306	Ratnasekera et al., 2022 [305]	• The Americas<br><br>• High Income Country	• Prevalence/Incidence<br><br>• Physical violence	• Not funded	• Quantitative	• Secondary data analysis	• Online	• Survivors<br><br>• Mixed Age Groups
S307	Rayhan & Akter, 2021 [306]	• South-East Asia<br><br>• Lower/ Middle Income Country	• Prevalence/Incidence<br><br>• Physical violence<br>• Sexual violence<br>• Emotional/Psychological<br>• Other	• Not funded	• Quantitative	• Primary data collection	• Face to face	• Survivors<br><br>• Age 15 - 24<br>• Age 25 - 50
S308	Razavi et al., 2023 [307]	• Africa<br>• South-East Asia<br>• The Americas<br><br>• Lower/ Middle Income Country	• Experience of violence<br><br>• Sexual violence<br>• Other	• Funded	• Qualitative	• Primary data collection	• Online	• Survivors<br><br>• Age group(s) not specified/ Unclear

S309	Rees et al., 2022 [308]	• Western Pacific<br><br>• High Income Country	• Prevalence/Incidence<br><br>• Physical violence<br>• Sexual violence<br>• Emotional/Psychological<br>• Other	• Funded	• Quantitative	• Primary data collection	• Face to face	• Survivors<br><br>• Age 15 - 24<br>• Age 25 - 50
S310	Reiber et al., 2022 [309]	• Europe<br><br>• High Income Country	• Prevalence/Incidence<br><br>• Other	• Funded	• Quantitative	• Primary data collection	• Online	• Survivors<br><br>• Mixed Age Groups
S311	Riggle et al., 2021 [310]	• The Americas<br><br>• High Income Country	• Experience of violence<br><br>• Physical violence	• Funded	• Qualitative	• Primary data collection	• Online<br>• Telephone	• Survivors<br><br>• Mixed Age Groups
S312	Rivera Rivera et al., 2023 [311]	• The Americas<br><br>• Lower/ Middle Income Country	• Prevalence/Incidence<br><br>• Physical violence<br>• Coercive control	• Funded	• Quantitative	• Primary data collection	• Online	• Survivors<br><br>• Mixed Age Groups
S313	Rockowitz et al., 2021 [312]	• Africa<br><br>• Lower/ Middle Income Country	• Experience of violence<br><br>• Sexual violence	• Funded	• Quantitative	• Primary data collection	• Face to face	• Survivors<br><br>• Age 15 - 24<br>• Mixed Age Groups
S314	Romahani & Rahman, 2022 [313]	• Western Pacific<br><br>• Lower/ Middle Income Country	• Prevalence/Incidence<br><br>• Physical violence<br>• Sexual violence<br>• Emotional/Psychological	• Funding not specified/Unclear	• Quantitative	• Primary data collection	• Not specified/unclear	• Survivors<br><br>• Mixed Age Groups
S315	Romito, Pellegrini, et al., 2022 [314]	• Europe<br><br>• High Income Country	• Prevalence/Incidence<br><br>• Physical violence<br>• Sexual violence<br>• Emotional/Psychological<br>• Coercive control	• Not funded	• Quantitative	• Primary data collection	• Face to face	• Survivors<br><br>• Mixed Age Groups

S316	Romito, Marchand-Martin, et al., 2022 [315]	• Europe<br><br>• High Income Country	• Prevalence/Incidence<br><br>• Physical violence<br>• Sexual violence<br>• Emotional/Psychological<br>• Coercive control	• Funding not specified/Unclear	• Quantitative	• Primary data collection	• Telephone	• Survivors<br><br>• Mixed Age Groups
S317	Roy et al., 2022 [316]	• Africa<br><br>• Lower/ Middle Income Country	• Prevalence/Incidence<br>• Interventions/services with Survivors to address violence<br><br>• Sexual violence	• Funded	• Mixed Methods	• Primary data collection	• Online	• Practitioner/ Policymaker<br><br>• Age group(s) not specified/ Unclear
S318	Royal, 2022 [317]	• Europe<br><br>• High Income Country	• Prevalence/Incidence<br>• Experience of violence<br><br>• Coercive control	• Funded	• Mixed Methods	• Primary data collection	• Online<br>• Telephone	• Survivors<br><br>• Mixed Age Groups
S319	Runkle et al., 2023 [318]	• The Americas<br><br>• High Income Country	• Prevalence/Incidence<br><br>• Other	• Funding not specified/Unclear	• Quantitative	• Secondary data analysis	• Online	• Survivors<br><br>• Mixed Age Groups
S320	Russell et al., 2022 [319]	• The Americas<br><br>• High Income Country	• Prevalence/Incidence<br><br>• Physical violence	• Funded	• Quantitative	• Secondary data analysis	• Not specified/unclear	• Survivors<br><br>• Age 15 - 24
S321	Sabri et al., 2020 [320]	• The Americas<br><br>• High Income Country	• Prevalence/Incidence<br><br>• Coercive control<br>• Other	• Funded	• Qualitative	• Primary data collection	• Online<br>• Telephone	• Survivors<br><br>• Mixed Age Groups
S322	Sacre et al., 2022 [321]	• Eastern Mediterranean<br><br>• Lower/ Middle Income Country	• Experience of violence<br><br>• Emotional/Psychological<br>• Other	• Not funded	• Quantitative	• Primary data collection	• Online	• Survivors<br><br>• Mixed Age Groups

S323	Salameh et al., 2020 [322]	• Eastern Mediterranean<br><br>• Lower/ Middle Income Country	• Experience of violence<br><br>• Physical violence<br>• Sexual violence<br>• Emotional/Psychological	• Not funded	• Quantitative	• Primary data collection	• Online	• Survivors<br><br>• Mixed Age Groups
S324	Salman et al., 2021 [323]	• Eastern Mediterranean<br><br>• High Income Country	• Prevalence/Incidence<br><br>• Physical violence<br>• Emotional/Psychological	• Funded	• Quantitative	• Primary data collection	• Online	• Survivors<br><br>• Mixed Age Groups
S325	Saloniki et al., 2022 [324]	• Europe<br><br>• High Income Country	• Prevalence/Incidence<br><br>• Physical violence<br>• Emotional/Psychological	• Funded	• Mixed Methods	• Primary data collection	• Online	• Survivors<br><br>• Mixed Age Groups
S326	Salt et al., 2021 [325]	• The Americas<br><br>• Lower/ Middle Income Country	• Prevalence/Incidence<br><br>• Physical violence<br>• Sexual violence	• Funded	• Quantitative	• Secondary data analysis	• Not specified/unclear	• Survivors<br><br>• Age 15 - 24
S327	Sánchez et al., 2023 [326]	• The Americas<br><br>• High Income Country	• Prevalence/Incidence<br><br>• Physical violence<br>• Emotional/Psychological<br>• Other	• Funded	• Quantitative	• Primary data collection	• Face to face	• Survivors<br><br>• Age 15 - 24<br>• Age 25 - 50
S328	Sanz-Barbero et al., 2021 [327]	• Europe<br><br>• High Income Country	• Prevalence/Incidence<br><br>• Sexual violence	• Funded	• Quantitative	• Primary data collection	• Online	• Survivors<br><br>• Age 15 - 24<br>• Age 25 - 50
S329	Sanz-Barbero et al., 2023 [328]	• Europe<br><br>• High Income Country	• Prevalence/Incidence<br><br>• Physical violence<br>• Sexual violence<br>• Other	• Funded	• Quantitative	• Secondary data analysis	• Not specified/unclear	• Survivors<br><br>• Mixed Age Groups
S330	Sapire et al., 2022 [329]	• The Americas	• Prevalence/Incidence<br>• Interventions/services with	• Funded	• Mixed Methods	• Primary data collection	• Online<br>• Telephone	• Practitioner/ Policymaker

		• High Income Country	Survivors to address violence<br>• Other			• Secondary data analysis		• Age group(s) not specified/ Unclear
S331	Sarı Doğan & Öztürk, 2022 [330]	• Europe<br>• Lower/ Middle Income Country	• Prevalence/Incidence<br>• Physical violence	• Funding not specified/Unclear	• Quantitative	• Secondary data analysis	• Not specified/unclear	• Survivors<br>• Mixed Age Groups
S332	Schaffer et al., 2023 [331]	• The Americas<br>• High Income Country	• Prevalence/Incidence<br>• Physical violence<br>• Other	• Not funded	• Quantitative	• Secondary data analysis	• Online	• Survivors<br>• Mixed Age Groups
S333	Schokkenbroek et al., 2021 [332]	• Europe<br>• High Income Country	• Prevalence/Incidence<br>• Physical violence	• Funded	• Quantitative	• Primary data collection	• Online	• Survivors<br>• Mixed Age Groups
S334	Sediri et al., 2020 [333]	• Eastern Mediterranean<br>• Lower/ Middle Income Country	• Prevalence/Incidence<br>• Other	• Funding not specified/Unclear	• Quantitative	• Primary data collection	• Online	• Survivors<br>• Mixed Age Groups
S335	Sefid Fard Jahromi et al., 2022 [334]	• Eastern Mediterranean<br>• Lower/ Middle Income Country	• Experience of violence<br>• Other	• Funding not specified/Unclear	• Quantitative	• Primary data collection<br>• Secondary data analysis	• Face to face<br>• Telephone	• Survivors<br>• Mixed Age Groups
S336	Sefidgarbaei & Mansouri, 2022 [335]	• Eastern Mediterranean<br>• Lower/ Middle Income Country	• Prevalence/Incidence<br>• Experience of violence<br>• Sexual violence	• Not funded	• Qualitative	• Primary data collection	• Telephone	• Survivors<br>• Mixed Age Groups

S337	Serrano Oswald, 2023 [336]	• The Americas<br><br>• Lower/ Middle Income Country	• Experience of violence<br><br>• Sexual violence<br>• Emotional/Psychological<br>• Femicide<br>• Other	• Funded	• Mixed Methods	• Primary data collection	• Online	• Survivors<br>• Practitioner/ Policymaker<br><br>• Age 25 - 50
S338	Serrano-Ibáñez et al., 2022 [337]	• Europe<br><br>• High Income Country	• Prevalence/Incidence<br><br>• Emotional/Psychological	• Funded	• Quantitative	• Primary data collection	• Online	• Survivors<br><br>• Mixed Age Groups
S339	Shahid et al., 2022 [338]	• Eastern Mediterranean<br><br>• Lower/ Middle Income Country	• Prevalence/Incidence<br><br>• Physical violence	• Not funded	• Quantitative	• Secondary data analysis	• Online	• Survivors<br><br>• Age 15 - 24<br>• Age 25 - 50
S340	Shahjalal et al., 2021 [339]	• South-East Asia<br><br>• Lower/ Middle Income Country	• Prevalence/Incidence<br><br>• Physical violence<br>• Sexual violence<br>• Emotional/Psychological	• Funding not specified/Unclear	• Quantitative	• Primary data collection	• Face to face	• Survivors<br><br>• Mixed Age Groups
S341	S. Sharma et al., 2021 [340]	• The Americas<br><br>• High Income Country	• Prevalence/Incidence<br><br>• Physical violence<br>• Sexual violence<br>• Emotional/Psychological<br>• Other	• Not funded	• Quantitative	• Secondary data analysis	• Online	• Survivors<br><br>• Age 15 - 24
S342	P. Sharma & Khokhar, 2022 [341]	• South-East Asia<br><br>• Lower/ Middle Income Country	• Prevalence/Incidence<br><br>• Physical violence<br>• Sexual violence<br>• Emotional/Psychological<br>• Coercive control	• Funding not specified/Unclear	• Quantitative	• Primary data collection	• Online	• Survivors<br><br>• Mixed Age Groups
S343	Shechory Bitton, 2023 [342]	• Europe<br><br>• High Income Country	• Experience of violence<br><br>• Physical violence<br>• Emotional/Psychological<br>• Other	• Funding not specified/Unclear	• Quantitative	• Primary data collection	• Online	• Survivors<br><br>• Mixed Age Groups

S344	Shewangzaw Engda et al., 2022 [343]	• Africa<br><br>• Lower/ Middle Income Country	• Prevalence/Incidence<br><br>• Physical violence<br>• Sexual violence<br>• Emotional/Psychological	• Funded	• Quantitative	• Primary data collection	• Face to face	• Survivors<br><br>• Age 15 - 24<br>• Age 25 - 50
S345	Shillington et al., 2022 [344]	• The Americas<br><br>• High Income Country	• Prevalence/Incidence<br>• Experience of violence<br><br>• Physical violence<br>• Sexual violence<br>• Emotional/Psychological<br>• Coercive control	• Funded	• Mixed Methods	• Primary data collection	• Online<br>• Telephone	• Survivors<br><br>• Age 50 +
S346	Shin & Choi, 2021 [345]	• Western Pacific<br><br>• High Income Country	• Prevalence/Incidence<br>• Experience of violence<br><br>• Emotional/Psychological	• Funded	• Quantitative	• Primary data collection	• Online	• Survivors<br>• Perpetrators<br><br>• Age 15 - 24
S347	Shitu et al., 2021 [346]	• Africa<br><br>• Lower/ Middle Income Country	• Prevalence/Incidence<br><br>• Physical violence<br>• Sexual violence<br>• Emotional/Psychological<br>• Other	• Not funded	• Quantitative	• Primary data collection	• Face to face	• Survivors<br><br>• Age 15 - 24<br>• Age 25 - 50
S348	Shoaei et al., 2022 [347]	• Eastern Mediterranean<br><br>• Lower/ Middle Income Country	• Experience of violence<br><br>• Physical violence<br>• Sexual violence<br>• Emotional/Psychological<br>• Coercive control	• Not funded	• Quantitative	• Primary data collection	• Online	• Survivors<br><br>• Mixed Age Groups
S349	Sigursteinsdottir & Karlsdottir, 2022 [348]	• Europe<br><br>• High Income Country	• Experience of violence<br><br>• Sexual violence<br>• Emotional/Psychological	• Not funded	• Quantitative	• Primary data collection	• Online	• Survivors<br><br>• Age group(s) not specified/ Unclear

S350	Sileo et al., 2023 [349]	• Africa<br><br>• Lower/ Middle Income Country	• Experience of violence<br><br>• Physical violence<br>• Other	• Funded	• Qualitative	• Primary data collection	• Face to face	• Survivors<br><br>• Age 15 - 24<br>• Age 25 - 50
S351	Singh & Duncan, 2022 [350]	• The America<br><br>• High Income Country	• Prevalence/Incidence<br><br>• Physical violence<br>• Sexual violence<br>• Emotional/Psychological	• Not funded	• Quantitative	• Secondary data analysis	• Face to face	• Survivors<br><br>• Mixed Age Groups
S352	Smith et al., 2022 [351]	• Europe<br><br>• Lower/ Middle Income Country	• Prevalence/Incidence<br><br>• Other	• Funded	• Quantitative	• Secondary data analysis	• Not specified/unclear	• Survivors<br><br>• Age group(s) not specified/ Unclear
S353	Soeiro et al., 2022 [352]	• The Americas<br><br>• Lower/ Middle Income Country	• Prevalence/Incidence<br><br>• Physical violence<br>• Sexual violence	• Funded	• Quantitative	• Primary data collection	• Face to face	• Survivors<br><br>• Age 15 - 24
S354	Sorenson et al., 2021 [353]	• The Americas<br><br>• High Income Country	• Prevalence/Incidence<br><br>• Physical violence<br>• Sexual violence<br>• Other	• Not funded	• Quantitative	• Secondary data analysis	• Not specified/unclear	• Not specified/ Unclear<br><br>• Age group(s) not specified/ Unclear
S355	Sosa Lovera et al., 2022 [354]	• The Americas<br><br>• Lower/ Middle Income Country	• Experience of violence<br><br>• Other	• Funding not specified/Unclear	• Quantitative	• Primary data collection	• Telephone	• Survivors<br>• Practitioner/ Policymaker<br><br>• Mixed Age Groups
S356	Souma et al., 2022 [355]	• Western Pacific<br><br>• High Income Country	• Prevalence/Incidence<br><br>• Other	• Funded	• Quantitative	• Primary data collection	• Online	• Survivors<br><br>• Age 15 - 24<br>• Age 25 - 50

S357	Speed et al., 2020 [356]	• Europe<br><br>• High Income Country	• Interventions/services with Survivors to address violence<br><br>• Other	• Not funded	• Quantitative	• Primary data collection	• Online	• Practitioner/ Policymaker<br><br>• Age group(s) not specified/ Unclear
S358	Stewart et al., 2021 [357]	• The Americas<br><br>• High Income Country	• Prevalence/Incidence<br><br>• Physical violence<br>• Sexual violence<br>• Emotional/Psychological	• Funded	• Quantitative	• Primary data collection	• Online	• Survivors<br><br>• Age 15 - 24
S359	Tadesse et al., 2022 [358]	• Africa<br><br>• Lower/ Middle Income Country	• Prevalence/Incidence<br><br>• Physical violence<br>• Sexual violence<br>• Emotional/Psychological<br>• Other	• Not funded	• Quantitative	• Primary data collection	• Face to face	• Survivors<br><br>• Age 50 +
S360	Teixeira et al., 2022 [359]	• Europe<br><br>• High Income Country	• Prevalence/Incidence<br><br>• Physical violence<br>• Sexual violence<br>• Emotional/Psychological	• Funded	• Quantitative	• Primary data collection	• Telephone	• Survivors<br><br>• Mixed Age Groups
S361	Tesfaw et al., 2021 [360]	• Africa<br><br>• Lower/ Middle Income Country	• Prevalence/Incidence<br><br>• Physical violence<br>• Sexual violence	• Funded	• Quantitative	• Primary data collection	• Face to face	• Survivors<br><br>• Mixed Age Groups
S362	Teshome et al., 2021 [361]	• Africa<br><br>• Lower/ Middle Income Country	• Prevalence/Incidence<br><br>• Physical violence<br>• Sexual violence<br>• Emotional/Psychological<br>• Other	• Funded	• Quantitative	• Primary data collection	• Face to face	• Survivors<br><br>• Mixed Age Groups

S363	Testoni et al., 2022 [362]	• Europe<br><br>• High Income Country	• Interventions/services with Survivors to address violence<br><br>• Other	• Funding not specified/Unclear	• Qualitative	• Primary data collection	• Not specified/unclear	• Practitioner/ Policymaker<br><br>• Mixed Age Groups
S364	Tharshini et al., 2021 [363]	• Western Pacific<br><br>• Lower/ Middle Income Country	• Experience of violence<br><br>• Sexual violence	• Funding not specified/Unclear	• Quantitative	• Primary data collection	• Online	• Survivors<br><br>• Mixed Age Groups
S365	Thiara & Roy, 2022 [364]	• Europe<br><br>• High Income Country	• Experience of violence<br><br>• Sexual violence<br>• Other	• Funding not specified/Unclear	• Mixed Methods	• Primary data collection	• Online	• Practitioner/ Policymaker<br><br>• Age group(s) not specified/ Unclear
S366	Tierolf et al., 2021 [365]	• Europe<br><br>• High Income Country	• Prevalence/Incidence<br>• Experience of violence<br><br>• Other	• Funded	• Mixed Methods	• Primary data collection	• Online<br>• Telephone	• Survivors<br>• Practitioner/ Policymaker<br><br>• Mixed Age Groups
S367	Tiesman et al., 2022 [366]	• The Americas<br><br>• High Income Country	• Experience of violence<br><br>• Physical violence<br>• Emotional/Psychological	• Funded	• Quantitative	• Secondary data analysis	• Online	• Perpetrators<br><br>• Age group(s) not specified/ Unclear
S368	Toccalino et al., 2022 [367]	• The Americas<br><br>• High Income Country	• Prevalence/Incidence<br><br>• Physical violence<br>• Sexual violence<br>• Emotional/Psychological	• Funding not specified/Unclear	• Qualitative	• Primary data collection	• Online	• Survivors<br>• Practitioner/ Policymaker<br><br>• Age group(s) not specified/ Unclear

S369	Todorovic et al., 2022 [368]	• The Americas<br><br>• High Income Country	• Prevalence/Incidence<br><br>• Physical violence<br>• Emotional/Psychological	• Funded	• Quantitative	• Primary data collection	• Online	• Survivors<br>• Perpetrators<br><br>• Mixed Age Groups
S370	Tosson & Saudi, 2021 [369]	• Eastern Mediterranean<br><br>• Lower/ Middle Income Country	• Prevalence/Incidence<br><br>• Physical violence<br>• Sexual violence<br>• Emotional/Psychological	• Not funded	• Quantitative	• Primary data collection	• Online	• Survivors<br><br>• Mixed Age Groups
S371	Tracy et al., 2022 [370]	• The Americas<br><br>• High Income Country	• Prevalence/Incidence<br><br>• Physical violence<br>• Sexual violence	• Not funded	• Quantitative	• Primary data collection	• Not specified/unclear	• Survivors<br><br>• Age 25 - 50
S372	Treglia et al., 2021 [371]	• Europe<br><br>• High Income Country	• Experience of violence<br><br>• Physical violence<br>• Femicide	• Not funded	• Qualitative	• Secondary data analysis	• Online	• Survivors<br>• Perpetrators<br><br>• Age 50 +
S373	Tripathi et al., 2023 [372]	• South-East Asia<br><br>• Lower/ Middle Income Country	• Experience of violence<br><br>• Physical violence<br>• Sexual violence<br>• Emotional/Psychological<br>• Acid attacks<br>• Coercive control	• Funding not specified/Unclear	• Quantitative	• Secondary data analysis	• Not specified/unclear	• Survivors<br><br>• Age group(s) not specified/ Unclear
S374	Tripathi et al., 2022 [373]	• South-East Asia<br><br>• Lower/ Middle Income Country	• Prevalence/Incidence<br>• Experience of violence<br><br>• Physical violence<br>• Sexual violence<br>• Emotional/Psychological<br>• Coercive control<br>• Other	• Funding not specified/Unclear	• Mixed Methods	• Primary data collection<br>• Secondary data analysis	• Not specified/unclear	• Survivors<br><br>• Age group(s) not specified/ Unclear

S375	Valencia Londoño et al., 2021 [374]	• The Americas<br><br>• Lower/ Middle Income Country	• Prevalence/Incidence<br><br>• Physical violence<br>• Sexual violence<br>• Femicide<br>• Other	• Funded	• Mixed Methods	• Secondary data analysis	• Online	• Survivors<br><br>• Age group(s) not specified/ Unclear
S376	van der Velden et al., 2022 [375]	• Europe<br><br>• High Income Country	• Prevalence/Incidence<br><br>• Physical violence<br>• Sexual violence	• Funded	• Quantitative	• Primary data collection	• Online	• Survivors<br><br>• Mixed Age Groups
S377	Vives-Cases, Parra-Casado, et al., 2021 [376]	• Europe<br><br>• High Income Country	• Prevalence/Incidence<br><br>• Other	• Funded	• Quantitative	• Secondary data analysis	• Telephone	• Survivors<br><br>• Age group(s) not specified/ Unclear
S378	Vives-Cases, La Parra-Casado, et al., 2021 [377]	• Europe<br><br>• High Income Country	• Interventions/services with Survivors to address violence<br><br>• Other	• Funded	• Qualitative	• Primary data collection	• Online<br>• Telephone	• Practitioner/ Policymaker<br><br>• Age group(s) not specified/ Unclear
S380	Vu et al., 2021 [378]	• The Americas<br><br>• High Income Country	• Prevalence/Incidence<br><br>• Other	• Funded	• Quantitative	• Primary data collection	• Online	• Survivors<br><br>• Mixed Age Groups
S381	Wada et al., 2022 [379]	• Africa<br><br>• Lower/ Middle Income Country	• Prevalence/Incidence<br><br>• Physical violence<br>• Sexual violence<br>• Emotional/Psychological<br>• Other	• Funding not specified/Unclear	• Quantitative	• Primary data collection	• Face to face	• Survivors<br><br>• Mixed Age Groups

S382	Walklate et al., 2022 [380]	• Europe<br><br>• High Income Country	• Interventions/services with Survivors to address violence<br><br>• Other	• Funded	• Qualitative	• Primary data collection	• Online	• Practitioner/ Policymaker<br><br>• Age group(s) not specified/ Unclear
S383	Wallace et al., 2022 [381]	• The Americas<br><br>• High Income Country	• Prevalence/Incidence<br><br>• Physical violence<br>• Sexual violence<br>• Emotional/Psychological<br>• Coercive control	• Funding not specified/Unclear	• Quantitative	• Primary data collection	• Online	• Survivors<br>• Perpetrators<br><br>• Mixed Age Groups
S384	W. Wang et al., 2020 [382]	• Western Pacific<br><br>• Lower/ Middle Income Country	• Experience of violence<br><br>• Emotional/Psychological	• Funded	• Quantitative	• Primary data collection	• Online	• Survivors<br><br>• Mixed Age Groups
S385	Y. Wang et al., 2022 [383]	• The Americas<br><br>• Lower/ Middle Income Country	• Prevalence/Incidence<br><br>• Physical violence<br>• Emotional/Psychological	• Funded	• Quantitative	• Primary data collection	• Not specified/unclear	• Survivors<br><br>• Mixed Age Groups
S386	Warren et al., 2022 [384]	• Western Pacific<br><br>• High Income Country	• Prevalence/Incidence<br><br>• Physical violence<br>• Sexual violence<br>• Emotional/Psychological<br>• Coercive control<br>• Other	• Funded	• Mixed Methods	• Primary data collection	• Online	• Practitioner/ Policymaker<br><br>• Age group(s) not specified/ Unclear

S387	Wathen et al., 2022 [385]	• The Americas<br><br>• High Income Country	• Interventions/services with Survivors to address violence<br><br>• Emotional/Psychological<br>• Coercive control<br>• Other	• Funded	• Qualitative	• Primary data collection	• Online<br>• Telephone	• Survivors<br>• Practitioner/ Policymaker<br><br>• Mixed Age Groups
S388	Wilson et al., 2021 [386]	• Europe<br><br>• High Income Country	• Experience of violence<br>• Interventions/services with Survivors to address violence<br><br>• Other	• Funded	• Mixed Methods	• Secondary data analysis	• Online	• Practitioner/ Policymaker<br><br>• Age group(s) not specified/ Unclear
S389	Wirawan et al., 2022 [387]	• South-East Asia<br><br>• Lower/ Middle Income Country	• Prevalence/Incidence<br>• Experience of violence<br><br>• Sexual violence<br>• Other	• Not funded	• Quantitative	• Primary data collection	• Online	• Survivors<br><br>• Mixed Age Groups
S390	Wong et al., 2022 [388]	• South-East Asia<br><br>• Lower/ Middle Income Country	• Experience of violence<br><br>• Physical violence<br>• Sexual violence<br>• Emotional/Psychological	• Not funded	• Quantitative	• Primary data collection	• Online	• Survivors<br><br>• Mixed Age Groups
S391	Wood et al., 2022 [389]	• The Americas<br><br>• Lower/ Middle Income Country	• Interventions/services with Survivors to address violence<br><br>• Sexual violence<br>• Other	• Not funded	• Quantitative	• Primary data collection	• Online	• Practitioner/ Policymaker<br><br>• Mixed Age Groups
S392	Wood et al., 2022 [390]	• Africa<br><br>• Lower/ Middle Income Country	• Prevalence/Incidence<br><br>• Physical violence<br>• Sexual violence<br>• Emotional/Psychological	• Funded	• Mixed Methods	• Primary data collection	• Online	• Survivors<br><br>• Age 15 - 24<br>• Age 25 - 50

S393	Workman et al., 2021 [391]	• Western Pacific<br><br>• High Income Country	• Prevalence/Incidence<br>• Experience of violence<br><br>• Other	• Funding not specified/Unclear	• Qualitative	• Secondary data analysis	• Not specified/unclear	• Not specified/ Unclear<br><br>• Age group(s) not specified/ Unclear
S394	Wu et al., 2022 [392]	• South-East Asia<br><br>• Lower/ Middle Income Country	• Prevalence/Incidence<br><br>• Physical violence<br>• Sexual violence<br>• Emotional/Psychological<br>• Other	• Funded	• Quantitative	• Primary data collection	• Online	• Survivors<br><br>• Age 15 - 24<br>• Age 25 - 50
S395	Xavier Hall et al., 2022 [393]	• The Americas<br><br>• High Income Country	• Experience of violence<br>• Sexual violence<br>• Other	• Not funded	• Quantitative	• Primary data collection	• Online	• Survivors<br><br>• Age 15 - 24
S396	Xu et al., 2022 [394]	• South-East Asia<br><br>• Lower/ Middle Income Country	• Experience of violence<br><br>• Physical violence<br>• Sexual violence<br>• Emotional/Psychological<br>• Other	• Not funded	• Quantitative	• Primary data collection	• Online	• Survivors<br><br>• Age 15 - 24<br>• Age 25 - 50
S397	Xue et al., 2020 [395]	Online (No specific location	• Prevalence/Incidence<br><br>• Physical violence<br>• Sexual violence<br>• Femicide<br>• Coercive control<br>• Other	• Funding not specified/Unclear	• Quantitative	• Secondary data analysis	• Online	• Not specified/ Unclear<br><br>• Age group(s) not specified/ Unclear
S398	Yamaoka et al., 2021 [396]	• Western Pacific	• Experience of violence<br><br>• Physical violence	• Funded	• Quantitative	• Primary data collection	• Online	• Perpetrators<br><br>• Age 15 - 24

		• High Income Country	• Emotional/Psychological<br>• Other					
S399	Yan et al., 2022 [397]	• Western Pacific<br><br>• Lower/ Middle Income Country	• Prevalence/Incidence<br><br>• Physical violence<br>• Emotional/Psychological<br>• Coercive control	• Funded	• Quantitative	• Primary data collection	• Telephone	• Survivors<br><br>• Age 50 +
S400	Yari et al., 2021 [398]	• Eastern Mediterranean<br><br>• Lower/ Middle Income Country	• Prevalence/Incidence<br><br>• Physical violence<br>• Sexual violence<br>• Emotional/Psychological<br>• Forced marriage	• Funded	• Quantitative	• Primary data collection	• Online	• Survivors<br><br>• Mixed Age Groups
S401	Yasmin et al., 2022 [399]	• Eastern Mediterranean<br><br>• Lower/ Middle Income Country	• Experience of violence<br><br>• Physical violence<br>• Sexual violence<br>• Emotional/Psychological<br>• Other	• Funding not specified/Unclear	• Quantitative	• Primary data collection	• Online	• Survivors<br><br>• Mixed Age Groups
S402	Yılmaz Karaman et al., 2022 [400]	• Europe<br><br>• Lower/ Middle Income Country	• Prevalence/Incidence<br><br>• Physical violence<br>• Sexual violence	• Funding not specified/Unclear	• Quantitative	• Secondary data analysis	• Online	• Survivors<br><br>• Age 15 - 24<br>• Age 25 - 50
S403	Yoosefi Lebni et al., 2021 [401]	• Eastern Mediterranean<br><br>• Lower/ Middle Income Country	• Experience of violence<br><br>• Other	• Funded	• Qualitative	• Primary data collection	• Face to face<br>• Online	• Survivors<br><br>• Age 15 - 24<br>• Age 25 - 50
S404	Yoosefi Lebni et al., 2022 [402]	• Eastern Mediterranean<br><br>• Lower/ Middle Income Country	• Experience of violence<br><br>• Physical violence<br>• Emotional/Psychological	• Funding not specified/Unclear	• Qualitative	• Primary data collection	• Face to face<br>• Telephone	• Survivors<br><br>• Mixed Age Groups

S405	Yoshioka et al., 2021 [403]	• Western Pacific<br>• High Income Country	• Experience of violence<br>• Other	• Funded	• Quantitative	• Primary data collection	• Online	• Survivors<br>• Mixed Age Groups
S406	Yousef et al., 2021 [404]	• Eastern Mediterranean<br>• Lower/ Middle Income Country	• Prevalence/Incidence<br>• Other	• Not funded	• Quantitative	• Primary data collection	• Online	• Survivors<br>• Age 15 - 24<br>• Age 25 - 50
S407	Zagloul et al., 2022[405]	• Eastern Mediterranean<br>• Lower/ Middle Income Country	• Experience of violence<br>• Sexual violence<br>• Emotional/Psychological	• Not funded	• Quantitative	• Primary data collection	• Online	• Survivors<br>• Mixed Age Groups
S408	Zaigham et al., 2022 [406]	• Europe<br>• High Income Country	• Experience of violence<br>• Physical violence<br>• Emotional/Psychological	• Funded	• Quantitative	• Primary data collection	• Online	• Survivors<br>• Age 15 - 24<br>• Age 25 - 50
S409	Zsilavecz et al., 2020 [407]	• Africa<br>• Lower/ Middle Income Country	• Prevalence/Incidence<br>• Physical violence	• Not funded	• Quantitative	• Secondary data analysis	• Online	• Survivors<br>• Age group(s) not specified/ Unclear
S410	Zulaika et al., 2022 [408]	• Africa<br>• Lower/ Middle Income Country	• Prevalence/Incidence<br>• Sexual violence	• Funded	• Quantitative	• Secondary data analysis	• Face to face	• Survivors<br>• Age 15 - 24
S411	Al-Rantisi & Faraj, 2022 [409]	• N/A<br>• Lower/ Middle Income Country	• Experience of violence<br>• Interventions/services with Survivors to address violence	• Not funded	• Qualitative	• Primary data collection	• Face to face	• Survivors<br>• Mixed Age Groups

			• Physical violence<br>• Sexual violence<br>• Emotional/Psychological<br>• Coercive control					
S412	Boxall & Morgan, 2021 [410]	• Western Pacific<br><br>• High Income Country	• Prevalence/Incidence<br><br>• Physical violence<br>• Sexual violence<br>• Coercive control	• Funding not specified/Unclear	• Quantitative	• Secondary data analysis	• Online	• Survivors<br><br>• Mixed Age Groups
S413	Cannon et al., 2022 [411]	• The Americas<br><br>• High Income Country	• Experience of violence<br><br>• Physical violence<br>• Sexual violence<br>• Emotional/Psychological<br>• Other	• Funding not specified/Unclear	• Quantitative	• Primary data collection	• Telephone	• Survivors<br><br>• Mixed Age Groups
S414	Dey & Tripathi, 2022 [412]	• South-East Asia<br><br>• Lower/ Middle Income Country	• Experience of violence<br><br>• Physical violence<br>• Other	• Funding not specified/Unclear	• Mixed Methods	• Primary data collection<br>• Secondary data analysis	• Face to face	• Survivors<br><br>• Age 50 +
S415	Jetelina et al., 2021 [413]	• The Americas<br><br>• High Income Country	• Prevalence/Incidence<br><br>• Physical violence<br>• Sexual violence<br>• Other	• Not funded	• Quantitative	• Primary data collection	• Online	• Survivors<br><br>• Mixed Age Groups
S416	Cantarero et al., 2022 [414]	• Europe<br><br>• High Income Country	• Prevalence/Incidence<br><br>• Other	• Not funded	• Quantitative	• Secondary data analysis	• Not specified/unclear	• Not specified/ Unclear<br><br>• Age group(s) not specified/ Unclear
S417	Morgan & Boxall, 2020 [415]	• Western Pacific<br><br>• High Income Country	• Experience of violence<br><br>• Physical violence<br>• Sexual violence	• Funding not specified/Unclear	• Quantitative	• Primary data collection	• Online	• Survivors<br><br>• Mixed Age Groups

S418	Morales-Arjona et al., 2022 [416]	• Europe<br>• High Income Country	• Prevalence/Incidence<br>• Emotional/Psychological	• Not funded	• Quantitative	• Primary data collection	• Face to face	• Survivors<br>• Perpetrators<br>• Mixed Age Groups
S419	Castillo & Moscoso, 2022 [417]	• The Americas<br>• Lower/ Middle Income Country	• Prevalence/Incidence<br>• Femicide	• Not funded	• Quantitative	• Secondary data analysis	• Not specified/unclear	• Survivors
S420	Bardales Mendoza et al., 2022 [418]	• The Americas<br>• Lower/ Middle Income Country	• Prevalence/Incidence<br>• Femicide	• Not funded	• Quantitative	• Secondary data analysis	• Not specified/unclear	• Survivors<br>• Age group(s) not specified/ Unclear
S421	Nabukeera, 2021 [419]	• Africa<br>• Lower/ Middle Income Country	• Experience of violence<br>• Other	• Funding not specified/Unclear	• Qualitative	• Secondary data analysis	• Not specified/unclear	• Not specified/ Unclear<br>• Age group(s) not specified/ Unclear

Table 2. Summary of Included Studies

Appendix E: Analysis by Type of Violence Addressed by Studies

Type of Violence	Studies (References)
**Physical**	N=250<br><br>S001, S002, S003, S006, S007, S008, S009, S010, S012, S013, S014, S015, S016, S017, S018, S020, S021, S024, S027, S029, S033, S035, S036, S037, S038, S039, S042, S044, S045, S046, S048, S053, S054, S055, S058, S059, S060, S061, S064, S069, S071, S072, S074, S075, S078, S079, S080, S081, S082, S083, S085, S086, S088, S089, S090, S094, S097, S099, S102, S103, S104, S106, S107, S108, S109, S110, S112, S113, S114, S116, S120, S121, S122, S123, S124, S126, S128, S130, S131, S132, S134, S136, S137, S139, S140, S142, S144, S145, S147, S148, S149, S152, S153, S159, S162, S164, S166, S167, S168, S169, S170, S171, S174, S175, S176, S177, S178, S179, S180, S184, S186, S188, S189, S190, S191, S192, S194, S195, S196, S197, S198, S199, S200, S202, S206, S208, S209, S213, S215, S216, S217, S218, S224, S225, S226, S227, S228, S232, S234, S237, S238, S239, S240, S242, S243, S244, S245, S248, S250, S253, S254, S255, S256, S258, S259, S260, S262, S264, S265, S267, S268, S269, S271, S273, S275, S276, S279, S283, S285, S286, S287, S288, S294, S295, S296, S297, S298, S301, S304, S305, S306, S307, S309, S311, S312, S314, S315, S316, S320, S323, S324, S325, S326, S327, S329, S331, S332, S333, S339, S340, S341, S342, S343, S344, S345, S347, S348, S350, S351, S353, S354, S358, S359, S360, S361, S362, S367, S368, S369, S370, S371, S372, S373, S374, S375, S376, S381, S383, S385, S386, S390, S392, S394, S396, S397, S398, S399, S400, S401, S402, S404, S408, S409, S411, S412, S413, S414, S415, S417
**Emotional/ Psychological**	N=212<br><br>S006, S007, S008, S009, S010, S012, S014, S016, S017, S018, S020, S021, S024, S025, S026, S027, S029, S031, S033, S035, S036, S037, S038, S039, S044, S045, S046, S048, S053, S054, S055, S058, S059, S060, S061, S064, S065, S069, S072, S073, S074, S078, S079, S080, S081, S082, S083, S084, S085, S087, S089, S090, S092, S094, S097, S102, S103, S104, S106, S107, S108, S110, S112, S113, S121, S122, S124, S125, S126, S127, S128, S130, S131, S132, S133, S134, S136, S137, S140, S144, S145, S146, S147, S148, S149, S151, S159, S162, S164, S166, S167, S168, S169, S170, S171, S173, S174, S175, S176, S177, S178, S179, S180, S181, S184, S186, S187, S188, S189, S190, S191, S192, S194, S195, S196, S199, S200, S206, S208, S209, S211, S212, S213, S215, S216, S217, S218, S224, S227, S232, S234, S237, S238, S240, S242, S243, S245, S247, S253, S254, S255, S256, S258, S259, S260, S267, S269, S275, S276, S279, S286, S287, S292, S294, S297, S300, S301, S304, S305, S307, S309, S314, S315, S316, S322, S323, S324, S325, S327, S337, S338, S340, S341, S342, S343, S344, S345, S346, S347, S348, S349, S351, S358, S359, S360, S362, S367, S368, S369, S370, S373, S374, S381, S383, S384, S385, S386, S387, S390, S392, S394, S396, S398, S399, S400, S401, S404, S407, S408, S411, S413, S418
**Sexual**	N=198<br><br>S001, S003, S004, S005, S006, S007, S008, S010, S011, S012, S014, S015, S016, S017, S020, S027, S032, S033, S035, S036, S038, S039, S041, S045, S046, S047, S050, S054, S055, S058, S060, S061, S067, S071, S072, S075, S079, S081, S082, S083, S085, S086, S087, S088, S089, S090, S096, S097, S106, S107, S108, S112, S113, S116, S120, S121, S122, S124, S126, S128, S130, S131, S132, S135, S136, S139, S140, S144, S145, S147, S148, S152, S153, S158, S159, S163, S165, S166, S167, S169, S170, S171, S173, S174, S175, S176, S180, S184, S185, S188, S189, S190, S192, S193, S197, S199, S202, S207, S208, S209, S215, S216, S218, S224, S225, S228, S238, S239, S240, S243, S244, S250, S251, S252, S253, S254, S256, S259, S262, S267, S270, S271, S273, S275, S276, S278, S282, S285, S286, S287, S288, S292, S294, S297, S302, S303, S304, S305, S307, S308, S309, S313, S314, S315, S316, S317, S323, S326, S328, S329, S336, S337, S340, S341, S342, S344, S345, S347, S348, S349, S351, S353, S354, S358, S359, S360, S361, S362, S364, S365, S368, S370, S371, S373, S374, S375, S376, S381, S383, S386, S389, S390, S391, S392, S394, S395, S396, S397, S400, S401, S402, S407, S410, S411, S412, S413, S415, S417

**Coercive control**	N= 76<br><br>S008, S010, S012, S014, S016, S021, S024, S025, S031, S032, S033, S036, S039, S044, S045, S046, S054, S055, S058, S060, S061, S064, S065, S075, S079, S083, S090, S092, S103, S104, S107, S112, S121, S125, S130, S131, S147, S149, S152, S159, S163, S164, S171, S173, S175, S177, S179, S199, S212, S229, S232, S245, S251, S256, S258, S260, S271, S291, S304, S312, S315, S316, S318, S321, S342, S345, S348, S373, S374, S383, S386, S387, S397, S399, S411, S412
**Intimate Partner Violence/ IPV**	N=73<br><br>S049, S057, S076, S088, S093, S096, S105, S112, S118, S120, S121, S129, S137, S145, S152, S156, S160, S172, S181, S183, S195, S201, S207, S209, S210, S223, S229, S230, S233, S237, S251, S256, S261, S263, S267, S272, S274, S275, S280, S282, S287, S289, S291, S297, S300, S302, S307, S308, S309, S310, S327, S329, S337, S343, S347, S350, S352, S356, S359, S362, S363, S375, S377, S378, S380, S391, S393, S394, S395, S396, S397, S413, S415
**Domestic Violence/ Abuse**	N=72<br><br>S015, S019, S022, S023, S025, S027, S028, S038, S040, S043, S056, S063, S068, S070, S077, S091, S095, S098, S099, S111, S118, S119, S131, S141, S143, S150, S157, S161, S172, S173, S198, S204, S205, S213, S214, S219, S220, S221, S237, S241, S245, S260, S265, S266, S269, S274, S281, S284, S290, S293, S303, S308, S321, S322, S332, S334, S341, S354, S355, S365, S366, S374, S382, S386, S387, S388, S398, S403, S405, S406, S414, S421
**Gender-based violence/ GBV**	N=23<br><br>S072, S115, S131, S141, S152, S173, S203, S213, S239, S257, S277, S292, S294, S308, S319, S330, S330, S337, S357, S375, S389, S416, S421
**Femicide**	N=17<br><br>S013, S034, S051, S060, S062, S101, S152, S159, S182, S202, S270, S337, S372, S375, S397, S419, S420
**Violence/ abuse**	N=15<br><br>S030, S063, S100, S103, S154, S164, S181, S186, S207, S222, S231, S246, S249, S299, S335
**Forced marriage**	N= 8<br><br>S027, S058, S060, S117, S131, S177, S225, S400
**Acid attacks**	N=3<br><br>S055, S225, S373
**Other**	Family violence* n=2 (S155, S401); Obstetric violence n=2 (S234, S236); Racial violence n=1 (S235); Concerns about violence n=1 (S052)<br>Savarna violence n=1 (S403)

Note: Corresponding study reference codes are provided in Table 2. Some studies address multiple types of violence. *While family violence and domestic violence by legal definition can be the same thing - i.e. violence between family members, we separated these studies based on the terminology/definition used by the study.

Table 3. Types of violence

Appendix F: Analysis by study research design

What Methods?	Studies (References)
Research Design: Quantitative studies	N=320
Quantitative cross-sectional studies	N=255<br><br>S002, S007, S008, S009, S010, S011, S012, S013, S014, S016, S017, S018, S020, S022, S026, S028, S029, S030, S032, S033, S036, S038, S039, S041, S046, S047, S048, S049, S050, S051, S052, S053, S054, S058, S059, S060, S061, S062, S063, S064, S065, S067, S068, S070, S072, S073, S074, S078, S079, S080, S081, S082, S083, S085, S086, S089, S093, S094, S095, S097, S099, S100, S102, S103, S104, S105, S106, S107, S109, S110, S111, S112, S113, S114, S116, S119, S120, S121, S124, S125, S126, S127, S128, S130, S131, S132, S133, S134, S135, S136, S137, S138, S139, S140, S141, S142, S146, S147, S150, S151, S154, S155, S158, S159, S160, S161, S162, S163, S165, S167, S168, S170, S171, S173, S174, S176, S177, S178, S179, S180, S181, S184, S185, S186, S190, S191, S192, S193, S194, S195, S196, S197, S199, S200, S202, S203, S206, S211, S212, S216, S218, S221, S229, S230, S231, S233, S234, S236, S238, S239, S240, S241, S242, S243, S244, S246, S247, S251, S253, S254, S256, S258, S259, S261, S262, S263, S266, S267, S268, S271, S273, S275, S276, S279, S281, S283, S286, S287, S289, S295, S296, S297, S298, S299, S301, S302, S304, S305, S307, S310, S312, S313, S314, S315, S316, S320, S322, S323, S324, S327, S328, S329, S331, S332, S333, S334, S335, S338, S340, S341, S342, S343, S344, S346, S347, S348, S349, S351, S353, S357, S358, S359, S360, S361, S362, S364, S370, S371, S376, S380, S381, S383, S384, S385, S389, S390, S391, S394, S396, S398, S399, S400, S401, S402, S405, S406, S407, S408, S412, S413, S415, S417, S418, S419, S420
Longitudinal quantitative	N=32<br><br>S035, S040, S055, S057, S096, S101, S123, S148, S169, S182, S201, S204, S208, S215, S226, S248, S252, S264, S265, S272, S277, S288, S293, S300, S306, S309, S326, S339, S356, S369, S395, S409
Quantitative media analysis	N=10<br><br>S034, S045, S056, S220, S225, S227, S367, S373, S39 , S416
Multi-stage quantitative	N=7<br><br>S006, S122, S188, S219, S250, S274, S377
Experimental studies	N=6<br><br>S069, S144, S172, S222, S355, S410
Quantitative helpline call analysis	N=6<br><br>S015, S091, S282, S285, S319, S354
Quasi-experimental studies	N=4<br><br>S042, S157, S284, S352
Research Design: Qualitative studies	N= 69
Qualitative cross-sectional studies	N=55<br><br>S003, S004, S005, S019, S027, S031, S037, S044, S071, S075, S087, S092, S108, S129, S143, S145, S149, S152, S156, S164, S166, S175, S187, S198, S207, S209, S213, S214, S217, S223, S228, S232, S235, S237, S245, S249, S255, S257, S269, S290, S292, S294, S303, S308, S311, S321, S336, S350, S363, S368, S378, S382, S387, S403, S404
Qualitative media analysis	N=9<br><br>S021, S023, S024, S025, S043, S189, S210, S393, S421

Qualitative case study	N=5<br><br>S001, S084, S098, S372, S411
Longitudinal qualitative	N=0
Multi-stage qualitative	N=0
Qualitative helpline call analysis	N=0
Research Design: Mixed Methods	N=30
Primary Method: Quantitative	N=23<br><br>S076, S077, S088, S090, S115, S117, S118, S153, S205, S224, S260, S270, S278, S280, S318, S325, S337, S366, S375, S386, S388, S392, S414
Primary Method: Qualitative	N=7<br><br>S183, S291, S317, S330, S345, S365, S374
**Data Type**	
Primary data collection	N=318<br><br>S001, S003, S004, S005, S006, S007, S008, S009, S010, S011, S012, S014, S016, S017, S018, S019, S022, S026, S027, S028, S029, S030, S031, S032, S033, S035, S036, S037, S038, S039, S041, S044, S046, S047, S048, S052, S053, S054, S057, S059, S061, S063, S064, S065, S067, S069, S070, S071, S073, S074, S075, S077, S078, S079, S080, S081, S082, S084, S085, S087, S088, S089, S090, S092, S093, S094, S095, S096, S097, S098, S100, S102, S103, S104, S105, S106, S107, S108, S109, S110, S111, S112, S113, S114, S115, S116, S117, S118, S120, S121, S122, S124, S125, S126, S127, S129, S130, S131, S132, S133, S134, S135, S136, S137, S139, S140, S144, S145, S146, S147, S148, S149, S150, S151, S152, S153, S154, S155, S156, S161, S162, S163, S164, S165, S166, S167, S168, S169, S170, S171, S172, S173, S174, S175, S176, S177, S179, S180, S181, S183, S184, S186, S187, S188, S191, S193, S194, S195, S196, S197, S198, S199, S200, S203, S206, S207, S208, S209, S211, S212, S213, S214, S216, S217, S218, S219, S221, S222, S223, S224, S228, S229, S230, S231, S232, S233, S234, S235, S236, S237, S238, S239, S240, S242, S243, S244, S245, S246, S247, S248, S249, S251, S253, S254, S255, S257, S258, S259, S260, S261, S262, S266, S267, S269, S271, S275, S276, S277, S278, S279, S280, S281, S286, S287, S288, S289, S290, S291, S292, S293, S294, S297, S298, S300, S301, S302, S303, S304, S305, S307, S308, S309, S310, S311, S312, S313, S314, S315, S316, S317, S318, S321, S322, S323, S324, S325, S327, S328, S330, S333, S334, S335, S336, S337, S338, S340, S342, S343, S344, S345, S346, S347, S348, S349, S350, S353, S355, S356, S357, S358, S359, S360, S361, S362, S363, S364, S365, S366, S368, S369, S370, S371, S374, S376, S378, S380, S381, S382, S383, S384, S385, S386, S387, S389, S390, S391, S392, S394, S395, S396, S398, S399, S400, S401, S403, S404, S405, S406, S407, S408, S411, S413, S414, S415, S417, S418
Secondary data analysis	N=107<br><br>S002, S013, S015, S020, S021, S023, S024, S025, S034, S040, S042, S043, S045, S049, S050, S051, S055, S056, S058, S060, S062, S068, S072, S076, S083, S086, S091, S095, S099, S101, S119, S123, S128, S141, S142, S143, S157, S158, S159, S160, S178, S182, S185, S188, S189, S190, S201, S202, S204, S205, S210, S215, S220, S225, S226, S227, S241, S250, S252, S256, S263, S264, S265, S268, S270, S272, S273, S274, S282, S283, S284, S285, S295, S296, S299, S306, S319, S320, S326, S329, S330, S331, S332, S335, S339, S341, S351, S352, S354, S367, S372, S373, S374, S375, S377, S388, S393, S397, S402, S409, S410, S412, S414, S416, S419, S420, S421
	Secondary analysis of administrative data e.g., hospital records)

	N=78 (a subset of the 107 studies above)<br><br>S002, S013, S015, S020, S034, S040, S042, S049, S050, S051, S055, S058, S060, S062, S068, S072, S083, S086, S091, S095, S099, S101, S119, S123, S128, S141, S142, S157, S158, S159, S160, S178, S182, S185, S190, S201, S202, S204, S205, S215, S226, S241, S250, S256, S263, S264, S265, S270, S272, S273, S282, S283, S284, S285, S295, S296, S299, S306, S319, S320, S326, S329, S331, S332, S335, S339, S341, S351, S352, S354, S372, S373, S375, S377, S402, S409, S419, S420
**Data Collection Interface**	
Online	N=264<br><br>S002, S003, S005, S008, S009, S010, S011, S012, S013, S014, S016, S019, S020, S021, S022, S024, S025, S027, S028, S030, S031, S032, S033, S038, S039, S041, S043, S044, S048, S052, S053, S056, S058, S059, S061, S063, S064, S065, S067, S068, S069, S073, S074, S077, S078, S079, S082, S083, S085, S086, S087, S090, S093, S094, S096, S097, S100, S102, S103, S105, S106, S107, S109, S110, S111, S112, S113, S114, S115, S116, S118, S119, S120, S121, S127, S129, S130, S132, S133, S134, S135, S137, S139, S140, S142, S143, S145, S146, S147, S149, S150, S151, S153, S154, S155, S156, S157, S158, S160, S161, S162, S163, S165, S166, S168, S169, S170, S171, S172, S173, S177, S178, S181, S185, S186, S189, S190, S191, S193, S194, S195, S199, S200, S205, S206, S208, S209, S210, S211, S212, S216, S217, S218, S220, S221, S223, S224, S225, S227, S229, S230, S232, S233, S235, S236, S237, S240, S242, S243, S244, S245, S246, S247, S248, S251, S253, S254, S257, S258, S261, S262, S264, S265, S266, S268, S269, S270, S271, S272, S275, S276, S277, S279, S280, S281, S286, S287, S288, S289, S290, S291, S292, S293, S295, S297, S299, S300, S301, S302, S303, S304, S305, S306, S308, S310, S311, S312, S317, S318, S319, S321, S322, S323, S324, S325, S328, S330, S332, S333, S334, S337, S338, S339, S341, S342, S343, S345, S346, S348, S349, S356, S357, S358, S364, S365, S366, S367, S368, S369, S370, S372, S375, S376, S378, S380, S382, S383, S384, S386, S387, S388, S389, S390, S391, S392, S394, S395, S396, S397, S398, S400, S401, S402, S403, S405, S406, S407, S408, S409, S412, S415, S417
Face to face	N=70<br><br>S001, S004, S005, S006, S017, S029, S035, S036, S037, S046, S050, S057, S070, S074, S075, S080, S084, S095, S098, S104, S108, S117, S124, S126, S131, S136, S144, S167, S172, S174, S175, S180, S184, S188, S196, S209, S213, S216, S219, S226, S228, S231, S239, S259, S260, S267, S277, S278, S307, S309, S313, S315, S327, S335, S340, S344, S347, S350, S351, S353, S359, S361, S362, S381, S403, S404, S410, S411, S414, S418
Telephone	N=64<br><br>S001, S003, S005, S007, S015, S018, S026, S044, S047, S052, S054, S058, S071, S081, S088, S090, S092, S098, S122, S125, S129, S144, S148, S152, S164, S175, S179, S183, S187, S197, S198, S203, S209, S214, S219, S222, S223, S234, S238, S255, S257, S260, S277, S285, S294, S298, S303, S311, S316, S318, S321, S330, S335, S336, S345, S355, S360, S366, S377, S378, S387, S399, S404, S413
Not specified/unclear	N=59<br><br>S023, S034, S040, S042, S045, S049, S051, S055, S060, S062, S072, S076, S081, S089, S091, S099, S101, S123, S128, S141, S159, S176, S182, S201, S202, S204, S205, S207, S215, S241, S249, S250, S252, S256, S263, S273, S274, S282, S283, S284, S285, S296, S314, S320, S326, S329, S331, S352, S354, S363, S371, S373, S374, S385, S393, S416, S419, S420, S421

Note: Corresponding study reference codes are provided in Table 2.

Table 4. Study Research Designs

Within the quantitative studies grouping, the most common design was cross-sectional studies (n=255 of 320 studies; 79.6%), followed by longitudinal studies (n=32; 10.0%). A small

number of studies used experimental (n=6; 1.9%), and quasi-experimental (n=4; 1.3%) designs. Other types of designs used were multi-stage (n=7; 2.2%) designs, as well as helpline call analyses (n=6; 1.9%) and media analysis (n=10; 3.1%). Within qualitative studies, the most common design was again cross-sectional studies (n=55 of 69 studies; 79.7%), followed by media analysis (n=9; 13.0%) and case studies (n=5; 7.2%). Although the majority of studies were quantitative, the results also indicate gaps in various study designs within this category, including longitudinal, experimental and quasi-experimental studies. These gaps also align with a lack of focus on interventions/services.

Appendix G: Breakdown of studies that measure self-reported incidents

**Studies that measure burden of violence (including studies of self-reported incidents)**	**N**	**% of studies of self-reported or incidents**
Studies that attempt to measure 'self-reported incidents' using quantitative methods	203	100%
Quantitative studies that **used surveys** to measure self-reported incidents	136	67.0%
Quantitative studies that **used the WHO multi-country study survey**[1] to measure self-reported incidents	17	12.4%
Quantitative studies that use WHO survey to measure self-reported incidents **and reported ethical methods**[2] **(data collection, safety, signposting)**	12	8.8%
Quantitative studies that use WHO survey to measure self-reported incidents + ethical methods (data collection, safety, signposting) **with a representative sample**	4	2.9%

[1] WHO. WHO multi-country study on women's health and domestic violence against women: summary report of initial results on prevalence, health outcomes and women's responses. Geneva, 2005.

[2] WHO. Putting women first: ethical and safety recommendations for research on domestic violence against women. Geneva, 2016.

Appendix H: Analysis by thematic topic, income context and research design

Thematic focus	Income Context	Research Design	N
Burden of violence	LMIC	Quantitative	N=151<br><br>S006, S007, S008, S009, S010, S012, S014, S015, S016, S017, S022, S026, S028, S029, S032, S033, S034, S035, S036, S039, S042, S046, S047, S048, S049, S054, S058, S059, S070, S072, S073, S074, S080, S085, S086, S089, S097, S101, S104, S109, S110, S111, S113, S114, S119, S120, S122, S124, S126, S130, S131, S136, S137, S141, S146, S148, S150, S151, S155, S158, S159, S161, S167, S170, S174, S179, S180, S184, S185, S186, S196, S197, S201, S202, S206, S208, S212, S215, S216, S218, S219, S220, S221, S222, S230, S234, S238, S239, S240, S242, S243, S253, S254, S256, S259, S261, S267, S273, S275, S279, S283, S285, S286, S293, S298, S299, S301, S304, S305, S307, S312, S313, S314, S322, S323, S326, S331, S334, S335, S339, S340, S342, S344, S347, S348, S352, S353, S355, S359, S361, S362, S364, S370, S373, S381, S384, S385, S389, S390, S394, S396, S399, S400, S401, S402, S406, S407, S409, S410, S419, S420
		Qualitative	N=36<br><br>S001, S003, S004, S005, S019, S021, S024, S025, S037, S043, S044, S071, S075, S098, S108, S149, S152, S175, S187, S189, S207, S213, S217, S228, S249, S255, S257, S269, S294, S308, S336, S350, S403, S404, S411, S421
		Mixed methods	N=13<br><br>S077, S153, S205, S224, S291, S318, S325, S330, S345, S365, S366, S386, S388
	HIC	Quantitative	N=150<br><br>S002, S011, S013, S018, S020, S030, S038, S040, S041, S045, S050, S051, S052, S053, S055, S056, S057, S060, S061, S062, S064, S067, S068, S079, S081, S082, S083, S091, S093, S094, S095, S096, S099, S100, S103, S105, S106, S107, S121, S123, S125, S127, S128, S132, S133, S135, S139, S140, S142, S147, S154, S157, S160, S162, S163, S165, S168, S169, S171, S172, S176, S177, S178, S181, S182, S188, S190, S191, S192, S193, S194, S195, S199, S203, S204, S226, S227, S229, S231, S233, S236, S241, S244, S246, S248, S250, S251, S252, S258, S262, S263, S264, S265, S266, S268, S271, S272, S274, S276, S281, S282, S284, S287, S288, S295, S296, S297, S300, S302, S306, S309, S310, S315, S316, S319, S320, S324, S327, S328, S329, S332, S333, S338, S341, S343, S346, S349, S351, S354, S356, S358, S360, S367, S369, S371, S376, S377, S380, S383, S395, S398, S405, S408, S412, S413, S415, S416, S417, S418
		Qualitative	N=22<br><br>S027, S031, S084, S087, S092, S129, S145, S156, S166, S198, S209, S223, S232, S235, S237, S245, S290, S311, S321, S368, S372, S393

		Mixed methods	N=13<br><br>S076, S088, S090, S183, S260, S270, S278, S317, S337, S374, S375, S392, S414
	LMIC & HIC	Quantitative	N=7<br><br>S078, S102, S112, S116, S134, S200, S225
		Qualitative	N=1<br><br>S292
		Mixed methods	N=1<br>S117
Interventions/ Services to address violence	LMIC	Quantitative	N=3<br>S069, S277, S391
		Qualitative	N=3<br>S164, S214, S411
		Mixed methods	N=1<br>S317
	HIC	Quantitative	N=6<br><br>S063, S065, S144, S211, S289, S357
		Qualitative	N=8<br><br>S129, S166, S223, S303, S363, S378, S382, S387
		Mixed methods	N=4<br>S077, S280, S330, S388
	LMIC & HIC	Quantitative	N=0
		Qualitative	N=0
		Mixed methods	N=1<br><br>S118

Note: Corresponding study reference codes are provided in Table 2

Table 5. Thematic focus, income context and research design of included studies

Appendix I: Analysis by study participants/target groups

What groups?	Studies (References)
Participants	
**Survivors**	N=373<br><br>S001, S002, S003, S004, S005, S006, S007, S008, S009, S010, S011, S012, S013, S014, S015, S016, S017, S018, S020, S021, S022, S024, S026, S028, S029, S030, S031, S032, S033, S034, S035, S036, S037, S038, S039, S040, S041, S044, S045, S046, S047, S048, S049, S050, S051, S052, S053, S054, S055, S056, S057, S058, S059, S060, S061, S062, S065, S067, S068, S069, S070, S071, S072, S073, S074, S075, S076, S078, S079, S080, S081, S082, S083, S085, S086, S087, S088, S089, S090, S091, S092, S093, S094, S095, S096, S097, S098, S099, S100, S101, S102, S103, S104, S105, S106, S107, S108, S109, S110, S111, S112, S113, S114, S116, S117, S119, S120, S121, S122, S123, S124, S125, S126, S127, S128, S129, S130, S131, S132, S133, S134, S135, S136, S137, S139, S140, S141, S142, S144, S145, S146, S147, S148, S149, S150, S151, S152, S153, S154, S155, S157, S158, S159, S160, S161, S162, S163, S164, S165, S166, S167, S168, S169, S170, S171, S172, S174, S175, S176, S177, S178, S179, S180, S181, S182, S183, S184, S185, S186, S187, S188, S189, S190, S191, S193, S194, S195, S196, S197, S198, S199, S200, S201, S202, S203, S204, S206, S207, S208, S209, S210, S211, S212, S214, S215, S216, S217, S218, S221, S222, S223, S224, S225, S226, S227, S228, S229, S230, S231, S232, S233, S234, S235, S236, S238, S239, S240, S241, S242, S243, S244, S245, S246, S247, S248, S250, S251, S252, S253, S254, S255, S256, S258, S259, S260, S262, S263, S264, S265, S266, S267, S268, S269, S270, S271, S272, S273, S274, S275, S276, S277, S278, S279, S280, S281, S282, S284, S286, S287, S288, S292, S293, S294, S295, S296, S297, S298, S299, S300, S301, S302, S304, S305, S306, S307, S308, S309, S310, S311, S312, S313, S314, S315, S316, S318, S319, S320, S321, S322, S323, S324, S325, S326, S327, S328, S329, S331, S332, S333, S334, S335, S336, S337, S338, S339, S340, S341, S342, S343, S344, S345, S346, S347, S348, S349, S350, S351, S352, S353, S355, S356, S358, S359, S360, S361, S362, S364, S366, S368, S369, S370, S371, S372, S373, S374, S375, S376, S377, S380, S381, S383, S384, S385, S387, S389, S390, S392, S394, S395, S396, S399, S400, S401, S402, S403, S404, S405, S406, S407, S408, S409, S410, S411, S412, S413, S414, 415, S417, S418, S419, S420
**Practitioner/ Policymaker**	N= 41<br><br>S004, S019, S027, S063, S065, S077, S084, S090, S115, S117, S118, S129, S145, S149, S156, S173, S205, S213, S223, S249, S257, S289, S290, S291, S294, S303, S317, S330, S337, S355, S357, S363, S365, S366, S368, S378, S382, S386, S387, S388, S391
**Perpetrators**	N=24<br><br>S039, S041, S042, S045, S064, S079, S086, S128, S137, S140, S154, S199, S212, S256, S263, S270, S346, S367, S369, S372, S383, S398, S418
Target Group	
**Survivors of violence/ abuse**	N=36<br><br>S086, S091, S092, S096, S106, S141, S142, S164, S174, S178, S185, S198, S210, S211, S223, S224, S241, S267, S270, S273, S280, S305, S313, S315, S318, S320, S329, S345, S368, S387, S402, S411, S413, S419, S420
**Pregnant/ Postpartum women**	N=30<br><br>S030, S035, S036, S046, S057, S081, S100, S102, S124, S146, S157, S162, S171, S172, S177, S193, S200, S201, S216, S233, S234, S236, S251, S259, S300, S309, S327, S362, S392, S394
**Service providers/ support workers**	N=28

	S019, S027, S063, S065, S077, S115, S156, S173, S205, S213, S223, S237, S245, S257, S289, S290, S291, S294, S303, S317, S325, S330,S355, S357, S363, S378, S386, S388
**Adolescents/ young people**	N=23<br><br>S038, S047, S064, S071, S088, S090, S109, S144, S155, S166, S183, S197, S207, S208, S260, S278, S294, S341, S346, S353, S358, S410, S418
**Healthcare worker/professional**	N=21<br><br>S029, S069, S073, S074, S085, S094, S108, S110, S114, S134, S170, S173, S193, S196, S206, S246, S253, S254, S279, S340, S384
**Married/partner**	N=17<br><br>S017, S080, S126, S151, S167, S194, S199, S214, S310, S314, S342, S343, S359, S374, S403, S412, S417
**Nationality/ethnicity specific**	N=12<br><br>S112, S149, S209, S220, S229, S235, S271, S276, S281, S302, S328, S407
**Parents/ mothers**	N=10<br><br>S033, S129, S148, S195, S221, S366, S369, S398, S406, S408
**Women of reproductive/ childbearing age**	N=4<br><br>S136, S147, S347, S350
**Students**	N=7<br><br>S011, S014, S154, S190, S191, S239, S247
**Refugee/ migrant/ asylum seeker group(s)**	N=9<br><br>S130, S207, S228, S231, S255, S292, S321, S353, S404
**Specifically focused on ethnic minorities**	N= 8<br><br>S013, S094, S096, S105, S117, S153, S258, S311, S380
**Job specific (varied roles)**	N= 7<br><br>S053, S054, S075, S169, S262, S336, S349
**Sex workers**	N=6<br><br>S001, S032, S130, S152, S222, S385
**Teacher/educator**	N=4<br><br>S120, S129, S235, S294
**Patients/ At risk**	N= 4<br><br>S037, S168, S180, S371
**Older people**	N=4<br><br>S104, S304, S399, S414
**Domestic workers**	N=2<br><br>S024, S098
**Police**	N=1<br><br>S382
**Other - Neighbours with an intention to report IPV**	N=1<br><br>S261

**No specific target group**	N=183<br><br>S002, S003, S004, S005, S006, S007, S008, S009, S010, S012, S016, S018, S020, S021, S022, S023, S025, S028, S031, S034, S039, S040, S041, S042, S043, S044, S045, S048, S049, S050, S051, S052, S055, S056, S058, S059, S060, S061, S062, S067, S068, S070, S072, S076, S078, S079, S082, S083, S087, S089, S093, S095, S097, S099, S101, S103, S107, S111, S113, S116, S119, S121, S122, S123, S125, S127, S128, S131, S132, S133, S135, S137, S139, S140, S143, S145, S150, S158, S159, S160, S161, S163, S175, S176, S179, S181, S182, S184, S186, S187, S188, S189, S199, S202, S203, S204, S212, S215, S218, S217, S219, S225, S226, S227, S230, S232, S238, S240, S242, S243, S244, S248, S250, S252, S256, S263, S264, S265, S266, S268, S269, S272, S274, S275, S277, S282, S283, S284, S285, S286, S288, S293, S295, S296, S297, S298, S299, S301, S306, S307, S308, S312, S316, S319, S322, S323, S324, S326, S331, S332, S333, S334, S335, S338, S339, S344, S348, S352, S354, S355, S356, S360, S361, S364, S367, S370, S372, S373, S375, S376, S377, S381, S383, S389, S390, S393, S396, S397, S400, S401, S405, S409, S415, S416, S421

Note: Corresponding study reference codes are provided in Table 2

Table 6. Groups/participant characteristics included in studies (Participants/ Target Groups)

Appendix J: Analysis by participants' age, gender, sex, sexual orientation

Age Group	**Studies (Reference)**
**Mixed**	N=283<br><br>S002, S005, S006, S007, S008, S009, S010, S014, S016, S017, S018, S019, S020, S022, S026, S028, S029, S030, S031, S032, S033, S035, S036, S037, S039, S040, S041, S044, S045, S046, S047, S048, S049, S050, S051, S052, S053, S054, S055, S058, S059, S060, S061, S062, S064, S065, S067, S068, S069, S072, S073, S074, S075, S076, S078, S079, S080, S081, S082, S083, S087, S088, S089, S092, S093, S095, S096, S097, S099, S100, S101, S102, S103, S105, S106, S107, S108, S109, S110, S111, S112, S113, S114, S116, S117, S120, S121, S122, S124, S125, S126, S129, S130, S131, S132, S134, S135, S136, S137, S139, S140, S143, S145, S146, S147, S150, S151, S154, S160, S161, S162, S163, S164, S165, S168, S170, S172, S174, S175, S176, S177, S178, S179, S180, S181, S184, S186, S187, S188, S193, S194, S195, S196, S198, S199, S200, S201, S203, S206, S209, S212, S213, S215, S216, S217, S218, S221, S223, S224, S226, S228, S229, S230, S231, S232, S233, S234, S235, S236, S237, S238, S240, S241, S242, S243, S244, S246, S248, S250, S252, S253, S254, S255, S256, S258, S259, S260, S261, S262, S263, S264, S265, S266, S269, S270, S271, S272, S273, S274, S275, S276, S281, S282, S286, S287, S288, S290, S292, S293, S295, S296, S297, S298, S300, S301, S302, S303, S305, S306, S307, S309, S310, S311, S312, S313, S314, S315, S316, S318, S319, S321, S322, S323, S324, S325, S327, S328, S329, S331, S332, S333, S334, S335, S336, S338, S339, S340, S342, S343, S344, S347, S348, S350, S351, S355, S356, S360, S361, S362, S363, S364, S366, S369, S370, S376, S380, S381, S383, S384, S385, S387, S389, S390, S391, S392, S394, S396, S400, S401, S402, S403, S404, S405, S406, S407, S408, S411, S412, S413, S415, S417, S418, S419
**Younger Women (< 25 yrs)**	N=35<br><br>S001, S004, S011, S012, S013, S038, S071, S086, S090, S144, S155, S158, S166, S183, S190, S191, S197, S207, S208, S222, S239, S267, S277, S278, S294, S299, S320, S326, S341, S346, S353, S358, S395, S398, S410
**Women aged 25-50 yrs**	N=12<br><br>S057, S085, S142, S153, S157, S199, S251, S279, S280, S289, S337, S371
**Older Women (> 50 yrs)**	N=11<br><br>S104, S133, S149, S169, S268, S304, S345, S359, S372, S399, S414
Gender/Sex	***N=395 studies reported sex/gender of participants***
**Women/Females**	N=383<br><br>S004, S005, S007, S008, S009, S010, S011, S012, S013, S014, S015, S016, S017, S018, S020, S021, S022, S023, S025, S028, S030, S031, S033, S035, S036, S037, S038, S041, S042, S043, S044, S046, S047, S053, S057, S061, S062, S063, S068, S074, S075, S076, S077, S078, S081, S082, S084, S087, S088, S089, S090, S091, S092, S093, S098, S100, S101, S102, S103, S107, S110, S112, S113, S120, S122, S124, S126, S134, S136, S139, S140, S141, S143, S145, S146, S147, S148, S149, S151, S152, S153, S154, S156, S157, S159, S162, S164, S167, S169, S171, S172, S174, S175, S176, S177, S180, S183, S187, S189, S193, S194, S195, S197, S200, S201, S203, S204, S207, S209, S211, S213, S214, S215, S216, S217, S218, S220, S223, S225, S233, S234, S236, S238, S240, S241, S242, S243, S244, S246, S248, S254, S255, S258, S259, S261, S262, S268, S269, S273, S276, S277, S279, S286, S288, S291, S293, S295, S297, S300, S301, S302, S303, S305, S307, S308, S309, S311, S312, S314, S315, S316, S321, S327, S329, S333, S334, S336, S337, S338, S340, S343, S344, S347, S348, S350, S353, S356, S359, S360, S362, S363, S368, S370, S372, S373, S374, S375, S376, S377, S378, S380, S389, S390, S392, S394, S397, S399, S400, S402, S403, S404, S405, S003, S026, S032, S039, S045, S052, S056, S059, S060, S065, S069, S070, S072, S073, S079, S083, S085, S094, S096, S097, S104, S108,

	S111, S114, S117, S131, S133, S135, S137, S142, S150, S161, S163, S170, S173, S176, S178, S179, S181, S188, S198, S199, S206, S212, S226, S227, S229, S231, S235, S244, S250, S257, S260, S265, S266, S270, S274, S279, S281, S282, S289, S292, S295, S304, S310, S313, S318, S319, S320, S322, S323, S324, S325, S326, S331, S335, S339, S341, S342, S345, S346, S349, S350, S351, S355, S364, S366, S367, S383, S384, S387, S388, S391, S401, S407, S001, S002, S019, S024, S034, S044, S048, S049, S050, S051, S054, S055, S058, S064, S067, S080, S086, S095, S099, S109, S116, S123, S127, S128, S129, S132, S145, S155, S160, S165, S166, S171, S184, S186, S190, S191, S193, S196, S208, S210, S222, S228, S230, S232, S238, S239, S245, S247, S252, S253, S263, S264, S271, S272, S275, S277, S280, S283, S296, S298, S299, S306, S332, S352, S358, S361, S369, S371, S381, S385, S396, S398, S409, S029, S125, S168, S328, S412, S413, S414, S415, S417, S418, S419, S421
**Men/Males**	N=182<br><br>S011, S013, S023, S038, S041, S042, S061, S063, S076, S077, S088, S103, S110, S134, S139, S140, S143, S176, S194, S195, S204, S207, S213, S217, S242, S243, S246, S254, S257, S261, S262, S277, S279, S293, S295, S302, S303, S333, S337, S340, S343, S350, S356, S368, S372, S375, S376, S378, S389, S405, S003, S026, S039, S045, S052, S056, S059, S065, S069, S070, S072, S073, S079, S083, S085, S094, S097, S104, S108, S111, S114, S117, S131, S133, S135, S137, S150, S161, S163, S170, S173, S176, S178, S179, S181, S188, S198, S199, S206, S212, S226, S227, S231, S250, S260, S265, S266, S270, S279, S282, S289, S292, S295, S304, S310, S319, S322, S323, S324, S325, S326, S331, S335, S339, S341, S342, S346, S350, S351, S355, S364, S366, S367, S383, S384, S388, S391, S401, S019, S048, S049, S050, S055, S058, S064, S067, S086, S095, S099, S105, S109, S116, S123, S127, S128, S132, S160, S186, S190, S191, S193, S196, S208, S245, S247, S253, S263, S271, S272, S275, S277, S283, S296, S298, S299, S306, S332, S352, S358, S361, S369, S395, S396, S398, S409, S029, S125, S168, S328, S413, S415, S418
**Other**	N=31<br><br>S023, S038, S056, S065, S094, S116, S121, S137, S139, S237, S246, S261, S267, S278, S294, S310, S318, S325, S345, S351, S387, S388, S391, S395, S406, S410, S105, S116, S144, S245, S413, S418<br><br>*This category included a diverse range of response options across studies including but not limited to '*other', 'prefer not to say', 'non-cis gender'*
**Non binary/ gender fluid**	N=14<br><br>S011, S083, S121, S199, S224, S229, S235, S250, S287, S318, S319, S345, S389, S418
**Transgender women**	N=13<br><br>S032, S083, S106, S121, S130, S199, S224, S250, S287, S319, S345, S395, S418
**Transgender men**	N=6<br><br>S083, S106, S199, S250, S319, S418
**Intersex**	N=0
Sexual orientation	***N= 35 studies reported sexual orientation of participants***
**Heterosexual**	N=34<br><br>S011, S067, S079, S082, S083, S092, S103, S105, S106, S116, S121, S127, S135, S139, S163, S165, S169, S190, S191, S199, S211, S224, S229, S258, S271, S275, S287, S302, S311, S328, S345, S389, S413, S418
**Bisexual**	N= 27<br><br>S011, S067, S079, S082, S083, S103, S105, S116, S127, S135, S139, S165, S169, S190, S191, S199, S211, S224, S229, S258, S271, S302, S311, S328, S345, S389, S418
**Other**	N=27

	S011, S079, S082, S083, S103, S105, S116, S121, S135, S139, S165, S169, S190, S191, S199, S211, S224, S229, S258, S271, S275, S287, S302, S311, S345, S389, S418<br>This category included a diverse range of response options across studies including but not limited to *'other', 'asexual', 'pansexual', 'queer', 'prefer not to say'*
**Lesbian**	N=24<br>S011, S067, S079, S082, S083, S092, S103, S116, S127, S169, S190, S191, S199, S211, S224, S229, S258, S271, S302, S311, S328, S389, S413, S418
**Gay**	N= 19<br>S011, S067, S079, S082, S083, S103, S105, S116, S135, S139, S190, S191, S199, S271, S302, S328, S345, S389, S418
**LGBTQI+**	N=5<br>S106, S121, S163, S319, S418

Note: Corresponding study reference codes are provided in Table 2

Table 7. Groups/participant characteristics included in studies (Age, Gender, Sex, Sexual Orientation)

Most studies reported the sex* or gender of participants (n= 395; 94.3%) and most of these studies involved women (n=383 of 395 studies; 96.9%). Studies also included men (n=182 of 419 studies; 43.4%), transgender women (n=13; 3.1%), transgender men (n=6; 1.4%), non-binary or gender fluid individuals (n=14; 3.3%), and 'other' gender identities (n=31; 7.4%). Those studies that didn't report participant sex or gender included media analyses, and studies with service providers/practitioners.

We also examined *how* studies reported sex and gender – i.e., what terminology was used. We found that of the 313 studies that reported participant 'gender', 213 (68.1%) reported participant gender as 'women' while 98 (31.3%) reported participant gender as 'female'. Six (1.9%) of these studies reported both 'women' and 'female' as response options in relation to participant gender. Of the 83 studies that reported participant sex, sex was reported as 'female' by 76 (91.6%) studies and reported as 'women' by four studies (4.8%).

Appendix K: Analysis by study setting

Setting	Studies (Reference)
Healthcare	N=82<br><br>S022, S029, S035, S036, S037, S046, S049, S055, S057, S065, S068, S069, S070, S072, S073, S074, S083, S084, S086, S094, S095, S099, S108, S115, S123, S128, S131, S134, S142, S146, S157, S158, S160, S166, S178, S180, S185, S196, S201, S213, S216, S226, S250, S252, S253, S256, S259, S264, S265, S268, S272, S274, S277, S282, S283, S295, S296, S299, S304, S306, S309, S319, S320, S326, S327, S331, S332, S335, S339, S340, S341, S352, S353, S362, S368, S371, S388, S392, S394, S402, S409, S414
Community-Mixed urban and rural	N=25<br><br>S003, S004, S008, S028, S032, S035, S040, S079, S082, S097, S104, S116, S117, S121, S124, S125, S130, S147, S174, S218, S231, S307, S368, S411, S412
Justice/Policing	N=23<br><br>S040, S051, S062, S101, S119, S159, S182, S202, S204, S241, S263, S282, S285, S329, S351, S354, S372, S373, S375, S377, S382, S419, S420
Community-Urban	N= 22<br><br>S002, S006, S007, S010, S026, S039, S071, S080, S089, S092, S098, S113, S164, S183, S209, S238, S278, S280, S293, S298, S301, S361
Online	N=13<br><br>S023, S043, S063, S078, S090, S135, S140, S189, S212, S225, S227, S367, S397
Community-Rural	N=12<br><br>S017, S071, S075, S080, S089, S113, S126, S183, S201, S238, S278, S350
Domestic	N= 9<br><br>S031, S112, S167, S188, S219, S267, S336, S342, S343
Education	N=9<br><br>S050, S071, S154, S184, S235, S247, S278, S410, S418
Workplace (general)	N=0
Church/religious	N=0
No setting specified*	N=241<br><br>S001, S005, S009, S011, S012, S013, S014, S015, S016, S018, S019, S020, S021, S024, S025, S027, S030, S033, S034, S038, S041, S042, S044, S045, S047, S048, S052, S053, S054, S056, S058, S059, S060, S061, S064, S067, S076, S077, S081, S085, S087, S088, S091, S093, S096, S100, S102, S103, S105, S106, S107, S109, S110, S111, S114, S118, S120, S122, S127, S129, S132, S133, S136, S137, S139, S141, S143, S144, S145, S148, S149, S150, S151, S152, S153, S155, S156, S161, S162, S163, S165, S168, S169, S170, S171, S172, S173, S175, S176, S177, S179, S181, S186, S187, S190, S191, S193, S194, S195, S197, S198, S199, S200, S203, S205, S206, S207, S208, S210, S211, S214, S215, S217, S220, S221, S222, S223, S224, S228, S229, S230, S232, S233, S234, S236, S237, S239, S240, S242, S243, S244, S245, S246, S248, S249, S251, S254, S255, S257, S258, S260, S261, S262, S266, S269, S270, S271, S273, S275, S276, S279, S281, S282, S284, S286, S287, S288, S289, S290, S291, S292, S294, S297, S300, S302, S303, S305, S308, S310, S311, S312, S313, S314, S315, S316, S317, S318, S321, S322, S323, S324, S325, S328, S330, S333, S334, S335, S337, S338, S344, S345, S346, S347, S348, S349, S355, S356, S357, S358, S359, S360, S363, S364, S365, S366, S369, S370, S374, S375, S376, S378, S380, S381, S383, S384, S385, S386, S387, S389, S390, S391,

	S393, S395, S396, S398, S399, S400, S401, S403, S404, S405, S406, S407, S408, S413, S415, S416, S417, S421

Note: Corresponding study reference codes are provided in Table 1. *These studies did not state a specific setting in which the study was conducted and so these studies were likely to cross-cut different settings. Some studies were conducted across multiple settings and are reported under each category as relevant.

Table 8. Study setting

Appendix L: Analysis by sustainable development goals (SDG) region of studies

Where? (SDG Regions)	
**Europe and Northern America**	N=177<br><br>S002, S011, S013, S018, S019, S025, S027, S030, S031, S038, S040, S041, S045, S050, S051, S052, S053, S055, S056, S057, S060, S061, S063, S064, S067, S068, S078, S081, S082, S083, S084, S087, S091, S092, S093, S094, S095, S096, S100, S102, S103, S105, S106, S107, S115, S116, S118, S121, S123, S125, S127, S128, S132, S133, S135, S139, S140, S142, S143, S144, S145, S153, S154, S157, S160, S162, S163, S165, S166, S168, S169, S171, S172, S176, S178, S182, S188, S190, S191, S193, S195, S198, S199, S200, S203, S205, S209, S211, S212, S223, S224, S225, S226, S227, S229, S231, S232, S233, S235, S236, S237, S241, S246, S248, S250, S251, S252, S258, S261, S263, S264, S266, S268, S272, S274, S280, S281, S282, S284, S287, S288, S289, S292, S295, S296, S297, S300, S302, S303, S306, S310, S311, S315, S316, S318, S319, S320, S321, S325, S326, S328, S329, S330, S332, S333, S338, S341, S345, S349, S351, S354, S357, S358, S360, S363, S365, S366, S367, S368, S369, S371, S372, S376, S377, S378, S380, S382, S387, S388, S395, S408, S413, S415, S416, S418
**Sub-Saharan Africa**	N=62<br><br>S001, S003, S004, S005, S006, S007, S026, S035, S037, S046, S047, S058, S071, S072, S088, S089, S090, S097, S115, S116, S117, S124, S130, S131, S136, S152, S175, S183, S184, S185, S186, S207, S217, S219, S222, S228, S238, S239, S249, S255, S269, S270, S273, S275, S277, S278, S293, S294, S308, S313, S317, S344, S347, S350, S359, S361, S362, S381, S392, S409, S410, S421
**Central and Southern Asia**	N=57<br><br>S014, S021, S022, S039, S043, S044, S049, S070, S073, S074, S075, S080, S098, S108, S122, S137, S148, S151, S161, S167, S174, S179, S180, S187, S189, S197, S214, S215, S216, S220, S225, S256, S257, S259, S260, S267, S283, S285, S286, S292, S294, S301, S305, S307, S308, S335, S339, S340, S342, S348, S373, S374, S394, S396, S400, S401, S414
**Northern Africa and Western Asia**	N=49<br><br>S008, S009, S010, S012, S016, S017, S020, S028, S029, S036, S059, S079, S110, S111, S112, S113, S114, S116, S119, S120, S146, S147, S149, S150, S194, S196, S200, S213, S218, S221, S230, S234, S240, S279, S292, S299, S322, S323, S324, S331, S334, S336, S343, S352, S370, S402, S406, S407, S411
**Eastern and South-Eastern Asia**	N=36<br><br>S024, S033, S034, S048, S054, S069, S104, S109, S115, S116, S126, S158, S164, S170, S177, S181, S202, S206, S208, S225, S257, S262, S271, S308, S314, S346, S356, S364, S384, S389, S390, S398, S399, S403, S404, S405
**Latin America and the Caribbean**	N=34<br><br>S015, S032, S042, S062, S085, S086, S101, S115, S116, S134, S141, S155, S159, S199, S201, S242, S243, S253, S254, S276, S298, S304, S308, S312, S327, S337, S353, S355, S375, S383, S385, S391, S419, S420
**Oceania**	N=20<br><br>S065, S077, S099, S115, S116, S129, S156, S412, S138, S204, S417, S244, S245, S265, S291, S290, S292, S309, S386, S393

Note: Corresponding study reference codes are provided in Table 2

Table 9. Sustainable Development Goal (SDG) Region of Studies

Appendix M: Analysis by WHO region of studies

Where? (WHO Regions)	Studies (References)
The Americas	N=115<br><br>S002, S011, S013, S015, S031, S032, S040, S042, S052, S053, S060, S061, S062, S082, S085, S086, S093, S094, S096, S101, S103, S105, S106, S115, S116, S121, S127, S134, S139, S141, S142, S145, S153, S154, S155, S159, S160, S162, S165, S169, S171, S172, S190, S191, S193, S199, S201, S205, S209, S211, S223, S224, S226, S227, S229, S231, S233, S235, S237, S242, S243, S250, S251, S252, S253, S254, S258, S264, S272, S276, S280, S282, S287, S289, S295, S298, S300, S302, S303, S304, S306, S308, S311, S312, S319, S320, S321, S326, S327, S330, S332, S337, S341, S345, S351, S353, S354, S355, S358, S367, S368, S369, S371, S375, S380, S383, S385, S387, S391, S395, S413, S415, S419, S420
Europe	N= 113<br><br>S012, S016, S018, S019, S025, S027, S030, S036, S038, S041, S045, S050, S051, S055, S056, S057, S059, S063, S064, S067, S068, S078, S079, S081, S083, S084, S087, S091, S092, S095, S100, S102, S107, S115, S116, S118, S119, S120, S123, S125, S128, S132, S133, S135, S140, S143, S144, S147, S150, S157, S163, S166, S168, S176, S178, S182, S188, S193, S194, S195, S198, S199, S200, S203, S212, S225, S230, S232, S236, S241, S246, S248, S261, S263, S266, S268, S274, S279, S281, S284, S288, S292, S296, S297, S299, S310, S315, S316, S318, S325, S328, S329, S331, S333, S338, S343, S349, S352, S357, S360, S363, S365, S366, S372, S376, S377, S378, S382, S388, S402, S408, S416, S418
Africa	N=62<br><br>S001, S003, S004, S005, S006, S007, S026, S035, S037, S046, S047, S058, S071, S072, S088, S089, S090, S097, S115, S116, S117, S124, S130, S131, S136, S152, S175, S183, S184, S185, S186, S207, S217, S219, S222, S228, S238, S239, S249, S255, S269, S270, S273, S275, S277, S278, S293, S294, S308, S313, S317, S344, S347, S350, S359, S361, S362, S381, S392, S409, S410, S421
Eastern Mediterranean	N=51<br><br>S008, S009, S010, S014, S017, S020, S021, S022, S028, S029, S033, S034, S039, S044, S098, S108, S110, S111, S112, S113, S114, S116, S122, S146, S151, S179, S196, S216, S218, S221, S225, S240, S257, S259, S292, S305, S322, S323, S324, S334, S335, S336, S339, S348, S370, S400, S401, S403, S404, S406, S407
South-East Asia	N=44<br><br>S043, S049, S070, S073, S074, S075, S080, S109, S115, S126, S137, S148, S161, S164, S167, S174, S180, S187, S189, S197, S214, S215, S220, S225, S256, S257, S260, S267, S283, S285, S286, S294, S301, S307, S308, S340, S342, S373, S374, S389, S390, S394, S396, S414
Western Pacific	N=43<br><br>S024, S048, S054, S065, S069, S077, S099, S104, S115, S116, S129, S156, S158, S170, S177, S181, S199, S202, S204, S206, S208, S244, S245, S257, S262, S265, S271, S290, S291, S292, S309, S314, S346, S356, S364, S384, S386, S393, S398, S399, S405, S412, S417

Note: Corresponding study reference codes are provided in Table 2

Table 10. WHO region of studies

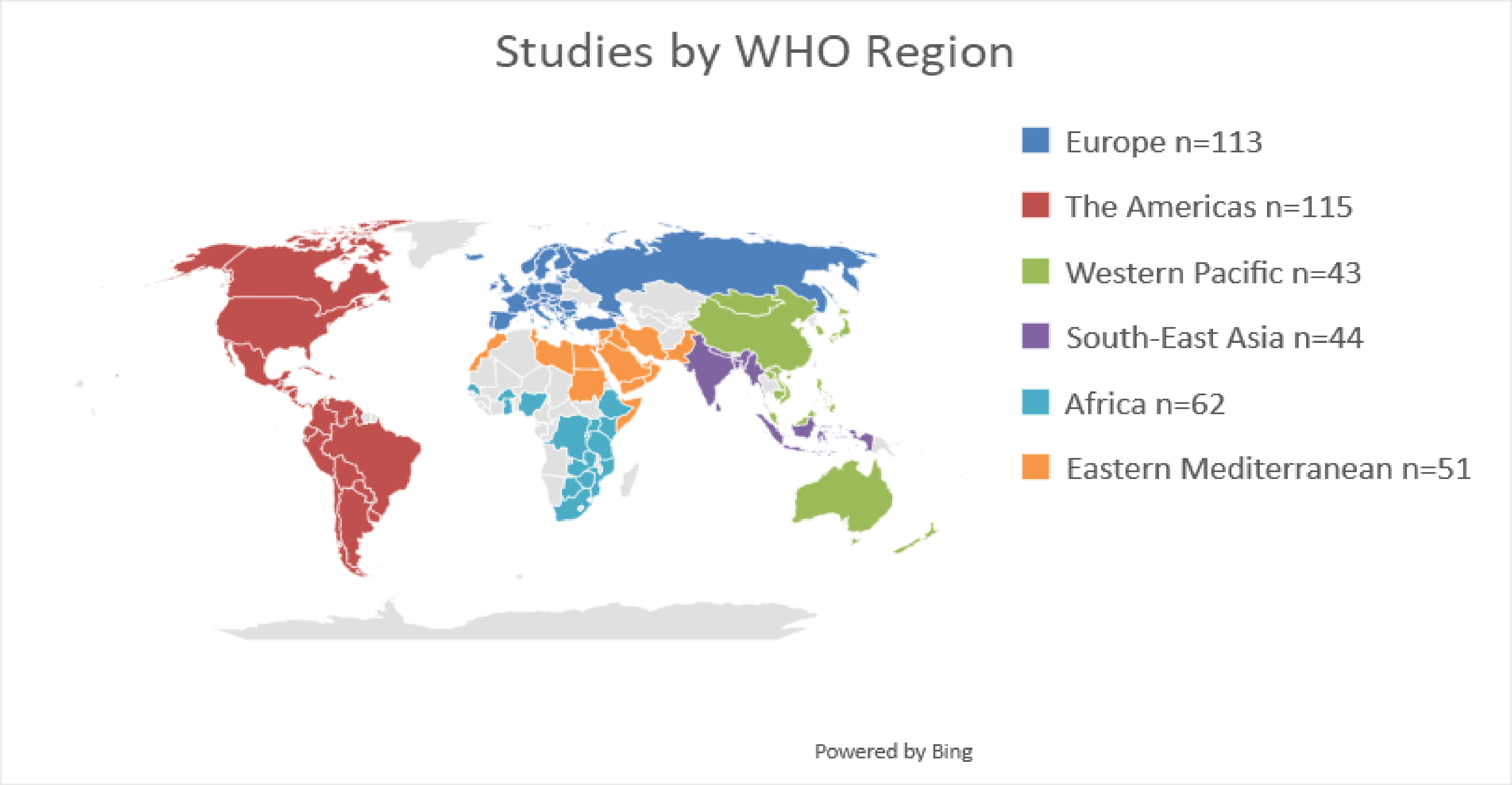

Figure X. Included studies by WHO World Region

All six WHO regions were represented in the current review. most studies were conducted in The Americas region (n=115; 27·4%), which includes North America, Latin America and the Caribbean) closely followed by a similar number of studies conducted in Europe (n=113; 26·8%) with fewer studies conducted in the Africa (n= 62; 14·7%), Eastern Mediterranean (n=51; 12·1%), South-East Asia (n=44; 10·5%), and the Western Pacific (n=43; 10·2%) regions.

References (Included Studies)

1 Aantjes C, Muchanga V, Munguambe K. Exposed and unprotected: Sex worker vulnerabilities during the COVID-19 health emergency in Mozambique. *Glob Public Health* 2022; **17**: 3568–82.

2 Abdallah HO, Zhao C, Kaufman E, *et al.* Increased Firearm Injury During the COVID-19 Pandemic: A Hidden Urban Burden. *J Am Coll Surg* 2021; **232**: 159-168.e3.

3 Aborisade RA. Accounts of Unlawful Use of Force and Misconduct of the Nigerian Police in the Enforcement of COVID-19 Measures. *J Police Crim Psychol* 2021; **36**: 450–62.

4 Aborisade RA. COVID-19 and Gender-Based Violence: Investigating the "Shadow Pandemic" of Sexual Violence During Crisis Lockdown in Nigeria. *Int J Offender Ther Comp Criminol* 2022; : 0306624X2211027.

5 Aborisade RA. Pandemic Policing and Police Sexual Misconduct: Voices of Women Sexually Abused by COVID-19 Enforcement Officers. *Women Crim Justice* 2022. DOI:10.1080/08974454.2022.2116965.

6 Abrahams Z, Lund C. Food insecurity and common mental disorders in perinatal women living in low socio-economic settings in Cape Town, South Africa during the COVID-19 pandemic: a cohort study. *Glob Ment Health (Camb)* 2022; **9**: 49–60.

7 Abrahams Z, Boisits S, Schneider M, Prince M, Lund C. The relationship between common mental disorders (CMDs), food insecurity and domestic violence in pregnant women during the COVID-19 lockdown in Cape Town, South Africa. *Soc Psychiatry Psychiatr Epidemiol* 2022; **57**: 37–46.

8 Abu-Elenin M, Elshora A, Sadaka MS, Abdeldaim D. Domestic violence against married women during the COVID-19 pandemic in Egypt. *BMC Womens Health* 2022; **22**: 94.

9 Abuhammad S. Violence against Jordanian Women during COVID-19 Outbreak. *Int J Clin Pract* 2021; **75**. DOI:10.1111/ijcp.13824.

10 Abujilban S, Mrayan L, Hamaideh S, Obeisat S, Damra J. Intimate Partner Violence Against Pregnant Jordanian Women at the Time of COVID-19 Pandemic's Quarantine. *J Interpers Violence* 2022; **37**: NP2442–64.

11 Adams-Clark AA, Freyd JJ. Undergraduates' Noncompliance with COVID-19 Regulations Is Associated with Lifetime Sexual Harassment Perpetration and Sexist Beliefs. *J Aggress Maltreat Trauma* 2022; **31**: 851–72.

12 Adibelli D, Sümen A, Teskereci G. Domestic violence against women during the Covid-19 pandemic: Turkey sample. *Health Care Women Int* 2021; **42**: 335–50.

13 Afif I, Gobaud A, Morrison C, *et al.* The changing epidemiology of interpersonal firearm violence during the COVID-19 pandemic in Philadelphia, PA. *Preventative Medicine: An International Journal Devoted to Practice & Theory* 2022; **158**: 1–8.

14 Aghamohseni H, Soltanieh H, Sh, Dehghanizadeh Z, Esfahani R, S. Depression, internet addiction and domestic violence on Iranian married women during the COVID-19 home quarantine. *Iranian Journal of War and Public Health* 2021; **13**: 125–9.

15 Aguero J. COVID-19 and the rise of intimate partner violence. *World Dev* 2021; **137**: 105217.

16 Akalin A, Ayhan F. Intimate Partner Violence against Women in Turkey during the COVID-19 Pandemic. *Issues Ment Health Nurs* 2022; **43**: 68–75.

17 Akel M, Berro J, Rahme C, Haddad C, Obeid S, Hallit S. Violence Against Women During COVID-19 Pandemic. *J Interpers Violence* 2022; **37**: NP12284–309.

18 Alcantara-Lopez M, Castro M, Martinez-Perez A, Fernandez V, Negron-Medina K, Lopez-Soler C. Children Exposed to Intimate Partner Violence During Confinement: Characteristics by Age and Sex. *Front Psychol* 2022; **13**: 889697.

19 Alexandru M, Kozma A, Baciu A. Aspects of the socio-medical situation of victims of domestic violence from romania in the context of the covid-19 pandemic. *Romanian Journal of Legal Medicine* 2021; **29**: 186–91.

20 Alharbi F, Alkheraiji M, Aljumah A, Al-Eissa M, Salman Q, Alaqeel M. Domestic Violence Against Married Women During the COVID-19 Quarantine in Saudi Arabia. *Cureus* 2021; **13**: e15231.

21 Ali R, Khalid A. COVID-19 and Domestic Violence in Pakistan: An Analysis of the Media Perspective. *J Int Womens Stud* 2021; **22**: 98–114.

22 Alimoradi Z, Bahrami N, Khodaparast S, Griffiths MD, Pakpour AH. Mediating role of psychological distress and domestic violence in the association of fear of COVID-19 with marital satisfaction and sexual quality of life among women of reproductive age: An Iranian cross-sectional study. *BMJ Open* 2023; **13**: e068916.

23 Al-Rawi A, Grepin K, Li X, Morgan R, Wenham C, Smith J. Investigating Public Discourses Around Gender and COVID-19: a Social Media Analysis of Twitter Data. *J Healthc Inform Res* 2021; **5**: 249–69.

24 Anam MZ, Warsito T, Al-Fadhat F, Pribadi U. COVID-19 and decent work: Online media coverage on Indonesian female migrant domestic workers in Malaysia and Taiwan. *Sociologia y Tecnociencia* 2021; **11**: 160–93.

25 Andreeva A, Drozhashchikh N, Nelaeva G. Women's Rights and the Feminists' "Dirty Plans": Media Discourses During the COVID-19 Pandemic in Russia. *Affilia - Journal of Women and Social Work* 2021; **36**: 319–35.

26 Angwenyi V, Kabue M, Chongwo E, *et al.* Mental health during covid-19 pandemic among caregivers of young children in kenya's urban informal settlements. A cross-sectional telephone survey. *Int J Environ Res Public Health* 2021; **18**. DOI:10.3390/ijerph181910092.

27 Anitha S, Gill AK. Domestic violence during the pandemic: 'By and for' frontline practitioners' mediation of practice and policies to support racially minoritised women 1. *Organization* 2022; **29**: 460–77.

28 Aolymat I. A cross-sectional study of the impact of COVID-19 on domestic violence, menstruation, genital tract health, and contraception use among women in Jordan. *American Journal of Tropical Medicine and Hygiene* 2021; **104**: 519–25.

29 Arafa A, Shehata A, Youssef M, Senosy S. Violence against healthcare workers during the COVID-19 pandemic: a cross-sectional study from Egypt. *Arch Environ Occup Health* 2022; **77**: 621–7.

30 Araujo-Chaveron L, Doncarli A, Vivanti AJ, *et al.* Perception of the SARS-CoV-2 pandemic by pregnant women during the first lockdown in France: worry, perceived vulnerability,

adoption and maintenance of prevention measures according to the Covimater study. *Prev Med Rep* 2022; **27**: 101807.

31 Archer-Kuhn B, Hughes J, Saini M, Still M, Beltrano N, Tam D. Who's Going to Keep Us Safe? Surviving Domestic Violence and Shared Parenting During Covid-19. *J Child Fam Stud* 2023; **32**: 57–66.

32 Aristegui I, Avila C, J, *et al.* Female sex workers and police violence during the Covid-19 health crisis in 2020-2021: results from the EPIC multi-country community-based research program in Argentina. *Harm Reduct J* 2022; **19**: 139.

33 Asadi N, Salmani F, Salmani M. The relationship between aggressive behaviors of preschool children and the violence against Iranian women in the COVID-19 pandemic. *BMC Womens Health* 2022; **22**: 406.

34 Asik G, Ozen EN. It takes a curfew: The effect of Covid-19 on female homicides. *Econ Lett* 2021; **200**: 109761.

35 Asratie MH. Domestic violence during COVID-19 pandemic among pregnant women registered for antenatal care and selected adverse pregnancy outcomes in Amhara region Ethiopia: Prospective cohort study design. *Clin Epidemiol Glob Health* 2022; **17**: 101146.

36 Atilla R, Yavuz A, Kocaöz S. Exposure of Pregnant Women to Intimate Partner Violence during the Pandemic in Turkey and Influencing Factors. *J Community Health Nurs* 2023; **40**: 1–13.

37 Atuhaire C, Rukundo GZ, Brennaman L, Cumber SN, Nambozi G. Lived experiences of Ugandan women who had recovered from a clinical diagnosis of postpartum depression: a phenomenological study. *BMC Pregnancy Childbirth* 2021; **21**. DOI:10.1186/s12884-021-04287-2.

38 Augusti EM, Sætren SS, Hafstad GS. Violence and abuse experiences and associated risk factors during the COVID-19 outbreak in a population-based sample of Norwegian adolescents. *Child Abuse Negl* 2021; **118**. DOI:10.1016/j.chiabu.2021.105156.

39 Bagheri Lankarani K, Hemyari C, Honarvar B, *et al.* Domestic violence and associated factors during COVID-19 epidemic: an online population-based study in Iran. *BMC Public Health* 2022; **22**: 774.

40 Baidoo L, Zakrison TL, Feldmeth G, Lindau ST, Tung EL. Domestic Violence Police Reporting and Resources During the 2020 COVID-19 Stay-at-Home Order in Chicago, Illinois. *JAMA Netw Open* 2021; **4**: e2122260.

41 Ballester-Arnal R, Nebot-Garcia J, Ruiz-Palomino E, Gimenez-Garcia C, Gil-Llario MD. 'INSIDE' project on sexual health in Spain: Sexual life during the lockdown caused by COVID-19. *Sexuality Research & Social Policy: A Journal of the NSRC* 2021; **18**: 1023–41.

42 Balmori de la Miyar JR, Hoehn-Velasco L, Silverio-Murillo A. Conflict resolution under the COVID-19 pandemic. *International Journal of Conflict Management* 2022; **33**: 291–310.

43 Banerjee P, Khandelwal C, Sanyal M. Deep care: The COVID-19 pandemic and the work of marginal feminist organizing in India. *Gend Work Organ* 2022. DOI:https://dx.doi.org/10.1111/gwao.12857.

44 Bankovskaya S, Maddahi J, Khachaki TL. From Isolation to Violence: Changes of the Domestic Environment in the Iranian Family under COVID-19*. *Russian Sociological Review* 2021; **20**: 86–110.

45 Barchielli B, Baldi M, Paoli E, *et al.* When “stay at home” can be dangerous: Data on domestic violence in Italy during COVID-19 lockdown. *Int J Environ Res Public Health* 2021; **18**. DOI:10.3390/ijerph18178948.

46 Belay AS, Gidafie AK, Gudeta TA. Perinatal intimate partner violence during COVID-19 and its associated factors among postpartum mothers attending newborn immunization in southern Ethiopia, 2021: A cross-sectional study. *SAGE Open Med* 2022; **10**: 20503121221116670.

47 Bevilacqua K, Williams A, Wood S, *et al.* Sexual harassment before and during the COVID-19 pandemic among adolescent girls and young women (AGYW) in Nairobi, Kenya: a cross-sectional study. *BMJ Open* 2022; **12**: e066777.

48 Bhandari D, Ozaki A, Suzuki T, *et al.* Physical and verbal abuse amid COVID-19: A nationwide cross-sectional survey in Japan. *BMJ Open* 2022; **12**. DOI:10.1136/bmjopen-2021-054915.

49 Bhattaram S, Shinde V, Lamba I, Gladwin R, Sharma KVS. Impact of COVID-19 lockdown on self-harm and violence among patients presenting to the emergency department. *Am J Emerg Med* 2022; **51**: 262–6.

50 Bidstrup JE, Busch JR, Munkholm J, Banner J. Impact of the Covid-19 lockdown on sexual assault cases in Eastern Denmark - a retrospective clinical forensic study. *Forensic Sci Med Pathol* 2022; **18**: 125–32.

51 Biehler-Gomez L, Maggioni L, Tambuzzi S, Kustermann A, Cattaneo C. Twenty years of femicide in Milan: A retrospective medicolegal analysis. *Sci Justice* 2022; **62**: 214–20.

52 Blair D-L, Shields M, Tonmyr L. Concerns about Household Violence during the COVID-19 Pandemic. *Int J Environ Res Public Health* 2022; **19**. DOI:https://dx.doi.org/10.3390/ijerph192214633.

53 Boyd K, Winslow V, Borson S, Lindau ST, Makelarski J. Caregiving in a Pandemic: Health-Related Socioeconomic Vulnerabilities Among Women Caregivers Early in the COVID-19 Pandemic. *Ann Fam Med* 2022; **20**: 406–13.

54 Brody C, Harrison N, Yi S. Income loss and gender-based violence during the COVID-19 pandemic among female entertainment workers in Cambodia: a cross-sectional phone survey. *BMC Public Health* 2023; **23**: 281.

55 Brown A, Collingwood P, Newton JL. Cohort study to explore the association between the COVID-19 pandemic lockdown and admissions for violence in North East and North Cumbria. *BMJ Open* 2021; **11**. DOI:10.1136/bmjopen-2021-052923.

56 Buchner V, Hamm S, Medenica B, Molendijk M. Linguistic Analysis of Online Domestic Violence Testimonies in the Context of COVID-19. *Sage Open* 2023; **13**: 21582440221146136.

57 Bueso-Izquierdo N, Daugherty J, Puente A, Caparros-Gonzalez R. Intimate partner violence and pregnancy during the COVID-19 pandemic. *J Gend Stud* 2022; **31**: 573–83.

58 Bukuluki P, Kisaakye P, Bulenzi-Gulere G, *et al.* Vulnerability to violence against women or girls during COVID-19 in Uganda. *BMC Public Health* 2023; **23**. DOI:10.1186/s12889-022-14951-7.

59 Çağlar EE, Hamzaoğlu N, Kurtça TT, Özcan YS. Investigation of Anxiety Levels and Domestic Violence During COVID-19 Outbreak in Context of Various Variables. *Neuropsychiatric Investigation* 2021; **59**: 89–95.

60 Cameron EC, Hemingway SL, Ray JM, Cunningham FJ, Jacquin KM. COVID-19 and Women: Key Components of SDG-5 and the Estimated Prevalence of Modern Slavery. *Int Perspect Psychol* 2021; **10**: 138–46.

61 Cannon CEB, Ferreira R, Buttell F, O'Connor A. Surviving intimate partner violence and disaster. *J Aggress Confl Peace Res* 2022. DOI:10.1108/JACPR-03-2022-0702.

62 Cantor E, Salas R, Torres R. Femicide and Attempted Femicide before and during the COVID-19 Pandemic in Chile. *Int J Environ Res Public Health* 2022; **19**. DOI:https://dx.doi.org/10.3390/ijerph19138012.

63 Caridade SMM, Saavedra R, Ribeiro R, *et al.* Remote support to victims of violence against women and domestic violence during the COVID-19 pandemic. *The Journal of Adult Protection* 2021; **23**: 302–16.

64 Cano-Lozano MC, Navas-Martínez MJ, Contreras L. Child-to-Parent Violence during Confinement Due to COVID-19: Relationship with Other Forms of Family Violence and Psychosocial Stressors in Spanish Youth. *Sustainability* 2021; **13**: 11431.

65 Carrington K, Morley C, Warren S, *et al.* The impact of COVID-19 pandemic on Australian domestic and family violence services and their clients. *Australian Journal of Social Issues* 2021; **56**: 539–58.

66 Casanovas LV-L, Serra L, Canals CS, *et al.* Prevalence of sexual harassment among young Spaniards before, during, and after the COVID-19 lockdown period in Spain. *BMC Public Health* 2022; **22**: 1888.

67 Chandan JS, Subramanian A, Chandan JK, *et al.* The risk of COVID-19 in survivors of domestic violence and abuse. *BMC Med* 2021; **19**: 246.

68 Chang Y-C, Hsu M-C, Ouyang W-C. Effects of Integrated Workplace Violence Management Intervention on Occupational Coping Self-Efficacy, Goal Commitment, Attitudes, and Confidence in Emergency Department Nurses: A Cluster-Randomized Controlled Trial. *Int J Environ Res Public Health* 2022; **19**: 2835.

69 Chhabra P, Mehta R, Bansal H, Kumar V, Mittal R. The impact of COVID-19 lockdown on mental health: A study from rural population of Haryana, India. *J Family Med Prim Care* 2022; **11**: 2896.

70 Chimbindi N, Ngema U, Ngwenya N, *et al.* The sexual and reproductive health needs of school-going young people in the context of COVID-19 in rural KwaZulu-Natal, South Africa. *African Journal of AIDS Research* 2022; **21**: 162–70.

71 Chime OH, Nduagubam OC, Orji CJ. Prevalence and patterns of gender-based violence in Enugu, Nigeria: a cross-sectional study. *Pan African Medical Journal* 2022; **41**: 198.

72 Chowdhury SR, Sunna TC, Das DC, *et al.* Mental health symptoms among the nurses of Bangladesh during the COVID-19 pandemic. *Middle East Current Psychiatry* 2021; **28**: 23.

73 Chowdhury SR, Kabir H, Mazumder S, Akter N, Chowdhury MR, Hossain A. Workplace violence, bullying, burnout, job satisfaction and their correlation with depression among Bangladeshi nurses: A cross-sectional survey during the COVID-19 pandemic. *PLoS One* 2022; **17**: e0274965.

74 Clisby SM, Choudhury T. Women Construction Workers in Bangladesh: Health, Wellbeing, and Domestic Abuse during the COVID-19 Pandemic. *Soc Sci* 2022; **11**: 83.

75 Cornell A, Diamond-Smith N, Mitchell A, Puri M. The COVID-19 Pandemic in the Nawalparasi District of Nepal: A Mixed Methods Assessment of Increased Alcohol Use and Intimate Partner Violence. *Res Sq* 2022. DOI:https://dx.doi.org/10.21203/rs.3.rs-1786122/v1.

76 Cortis N, Smyth C, valentine K, Breckenridge J, Cullen P. Adapting Service Delivery during COVID-19: Experiences of Domestic Violence Practitioners. *The British Journal of Social Work* 2021; **51**: 1779–98.

77 Costa R, Rodrigues C, Dias H, *et al.* Quality of maternal and newborn care around the time of childbirth for migrant versus nonmigrant women during the COVID-19 pandemic: Results of the IMAgiNE EURO study in 11 countries of the WHO European region. *International Journal of Gynecology & Obstetrics* 2022; **159**: 39–53.

78 Cunha O, Caridade S, de Castro Rodrigues A, Cruz AR, Peixoto MM. Perpetration of Intimate Partner Violence and COVID-19-Related Anxiety During the Second Lockdown in Portugal: The Mediating Role of Anxiety, Depression, and Stress. *J Fam Violence* 2023; : 1–12.

79 Das T, Roy TB, Roy R. Reintegration with family and intimate partner violence (IPV) against women among the returnee migrant worker's family during COVID-19 induced lockdown: A Block-level analysis using multinomial logistic regression model. *Child Youth Serv Rev* 2021; **130**: 106226.

80 Daugherty JC, Bueso-Izquierdo N, Lara-Cinisomo S, Lozano-Ruiz A, Caparros-Gonzalez RA. Partner relationship quality, social support and maternal stress during pregnancy and the first COVID-19 lockdown. *Journal of Psychosomatic Obstetrics & Gynecology* 2022; **43**: 563–73.

81 Davidson CA, Jackson KT, Kennedy K, Stoyanovich E, Mantler T. Vaccine Hesitancy Among Canadian Mothers: Differences in Attitudes Towards a Pediatric COVID-19 Vaccine Among Women Who Experience Intimate Partner Violence. *Matern Child Health J* 2023; **27**: 566–74.

82 Day S, Kellett C, Jones S. Domestic abuse and violence reporting by service users of an online sexually transmitted infection testing service, during the Coronavirus-19 pandemic. *Int J STD AIDS* 2023; **34**: 328–31.

83 de Gennaro D, Loia F, Piscopo G. Working role of women in a pandemic world: a poetic inquiry. *Management Research Review* 2022; **45**: 1373–92.

84 De la Cerda-Vargas MF, Stienen MN, Campero A, *et al.* Burnout, Discrimination, Abuse, and Mistreatment in Latin America Neurosurgical Training During the Coronavirus Disease 2019 Pandemic. *World Neurosurg* 2022; **158**: e393–415.

85 de Oliveira SMT, Galdeano EA, da Trindade EMGG, *et al.* Epidemiological Study of Violence against Children and Its Increase during the COVID-19 Pandemic. *Int J Environ Res Public Health* 2021; **18**: 10061.

86 Basílio de Simões R, Amaral I, Santos S, Alcantara J. Online Violence Against Women: Reports From the COVID-19 Pandemic Experience. *Comunicação e Sociedade* 2022; **42**: 179–203.

87 Decker MR, Wood SN, Thiongo M, *et al.* Gendered health, economic, social and safety impact of COVID-19 on adolescents and young adults in Nairobi, Kenya. *PLoS One* 2021; **16**: e0259583.

88 Decker MR, Wood SN, Thomas HL, *et al.* Violence against women from partners and other household members during COVID-19 in Burkina Faso and Kenya. *BMC Public Health* 2022; **22**: 1857.

89 Decker MR, Bevilacqua K, Wood SN, *et al.* Gender-based violence during COVID-19 among adolescent girls and young women in Nairobi, Kenya: a mixed-methods prospective study over 18 months. *BMJ Glob Health* 2022; **7**: e007807.

90 Del Casale A, Modesti MN, Lai C, *et al.* Calls to the anti-violence number in Italy during COVID-19 pandemic: correlation and trend analyses of violence reports during 2020. *Soc Psychiatry Psychiatr Epidemiol* 2022; **57**: 2503–10.

91 Desai R, Bandyopadhyay S, Zafar S, Bradbury-Jones C. The Experiences of Post-Separation Survivors of Domestic Violence During the Covid-19 Pandemic: Findings From a Qualitative Study in the United Kingdom. *Violence Against Women* 2022; : 107780122211429.

92 Devoto A, Himelein-Wachowiak M, Liu T, Curtis B. Women's Substance Use and Mental Health During the COVID-19 Pandemic. *Women's Health Issues* 2022; **32**: 235–40.

93 Dholakia J, Lee YW, Lu KH, *et al.* Identity-related experiences of Asian American trainees in gynecologic oncology. *Gynecol Oncol Rep* 2022; **44**: 101097.

94 Di Franco M, Martines GF, Carpinteri G, Trovato G, Catalano D. Domestic violence detection amid the COVID-19 pandemic: the value of the WHO questionnaire in emergency medicine. *QJM: An International Journal of Medicine* 2021; **114**: 637–41.

95 Diaz A, Nucci-Sack A, Colon R, *et al.* Impact of COVID-19 Mitigation Measures on Inner-City Female Youth in New York City. *Journal of Adolescent Health* 2022; **70**: 220–7.

96 Ditekemena JD, Luhata C, Mavoko HM, *et al.* Intimate Partners Violence against Women during a COVID-19 Lockdown Period: Results of an Online Survey in 7 Provinces of the Democratic Republic of Congo. *Int J Environ Res Public Health* 2021; **18**: 5108.

97 Dogar AA, Shah I, Mahmood T, Elahi N, Alam A, Jadoon UG. Impact of Covid-19 on informal employment: A case study of women domestic workers in Khyber Pakhtunkhwa, Pakistan. *PLoS One* 2022; **17**: e0278710.

98 Domínguez D JF, Truong J, Burnett J, *et al.* Effects of the Response to the COVID-19 Pandemic on Assault-Related Head Injury in Melbourne: A Retrospective Study. *Int J Environ Res Public Health* 2022; **20**: 63.

99 Doncarli A, Araujo-Chaveron L, Crenn-Hebert C, *et al.* Impact of the SARS-CoV-2 pandemic and first lockdown on pregnancy monitoring in France: the COVIMATER cross-sectional study. *BMC Pregnancy Childbirth* 2021; **21**: 799.

100 dos Santos ETM, de Oliveira CM, Furtado BMASM, da Costa HV V., do Bonfim C V. Female Homicide and the COVID-19 Pandemic in a State of the Northeast Region of Brazil. *Homicide Stud* 2022; **26**: 403–18.

101 Drandić D, Drglin Z, Mihevc Ponikvar B, *et al.* Women's perspectives on the quality of hospital maternal and newborn care around the time of childbirth during the COVID -19

pandemic: Results from the IMAgiNE EURO study in Slovenia, Croatia, Serbia, and Bosnia-Herzegovina. *International Journal of Gynecology & Obstetrics* 2022; **159**: 54–69.

102 Drotning KJ, Doan L, Sayer LC, Fish JN, Rinderknecht RG. Not All Homes Are Safe: Family Violence Following the Onset of the Covid-19 Pandemic. *J Fam Violence* 2023; **38**: 189–201.

103 Du P, Chen Y. Prevalence of elder abuse and victim-related risk factors during the COVID-19 pandemic in China. *BMC Public Health* 2021; **21**: 1096.

104 Duncan DT, Park SH, Chen Y-T, *et al.* Sleep characteristics among black cisgender sexual minority men and black transgender women during the COVID-19 pandemic: The role of multi-level COVID-19-related stressors. *Sleep Health* 2022; **8**: 440–50.

105 Eaton AA, Ramjee D, Saunders JF. The Relationship between Sextortion during COVID-19 and Pre-pandemic Intimate Partner Violence: A Large Study of Victimization among Diverse U.S Men and Women. *Vict Offender* 2023; **18**: 338–55.

106 Ebert C, Steinert JI. Prevalence and risk factors of violence against women and children during COVID-19, Germany. *Bull World Health Organ* 2021; **99**: 429–38.

107 Ebrahimi Rigi Z, Mangolian Shahrbabaki P, Ahmadi F, Ravari A. Self-Sacrifice in a Distressful and Threatening Environment: The Consequences of the COVID-19 Crisis in Intensifying Workplace Violence. *Front Psychiatry* 2022; **13**. DOI:10.3389/fpsyt.2022.848059.

108 Ekawati R, Rahma AN, Alifia K, Cahyani NRA, Susantini P. Physical abuse in adolescents during the COVID-19 pandemic. *J Public Health Afr* 2022; **13**. DOI:10.4081/jphia.2022.2413.

109 Elhadi M, Msherghi A, Elgzairi M, *et al.* Burnout Syndrome Among Hospital Healthcare Workers During the COVID-19 Pandemic and Civil War: A Cross-Sectional Study. *Front Psychiatry* 2020; **11**. DOI:10.3389/fpsyt.2020.579563.

110 Elhadi M, Alsoufi A, Msherghi A, *et al.* Psychological Health, Sleep Quality, Behavior, and Internet Use Among People During the COVID-19 Pandemic: A Cross-Sectional Study. *Front Psychiatry* 2021; **12**. DOI:10.3389/fpsyt.2021.632496.

111 El-Nimr NA, Mamdouh HM, Ramadan A, El Saeh HM, Shata ZN. Intimate partner violence among Arab women before and during the COVID-19 lockdown. *Journal of the Egyptian Public Health Association* 2021; **96**: 15.

112 Elsaid NMAB, Shehata SA, Sayed HH, Mohammed HS, Abdel-Fatah ZF. Domestic violence against women during coronavirus (COVID-19) pandemic lockdown in Egypt: a cross-sectional study. *Journal of the Egyptian Public Health Association* 2022; **97**: 23.

113 Elsaid NMAB, Ibrahim O, Abdel-Fatah ZF, *et al.* Violence against healthcare workers during coronavirus (COVID-19) pandemic in Egypt: a cross-sectional study. *Egypt J Forensic Sci* 2022; **12**: 45.

114 Endler M, Al-Haidari T, Benedetto C, *et al.* How the coronavirus disease 2019 pandemic is impacting sexual and reproductive health and rights and response: Results from a global survey of providers, researchers, and policy-makers. *Acta Obstet Gynecol Scand* 2021; **100**: 571–8.

115 Erausquin JT, Tan RKJ, Uhlich M, *et al.* The International Sexual Health And REproductive Health during COVID-19 (I-SHARE) Study: A Multicountry Analysis of Adults from 30

Countries Prior to and During the Initial Coronavirus Disease 2019 Wave. *Clinical Infectious Diseases* 2022; **75**: e991–9.

116 Esho T, Matanda DJ, Abuya T, *et al.* The perceived effects of COVID-19 pandemic on female genital mutilation/cutting and child or forced marriages in Kenya, Uganda, Ethiopia and Senegal. *BMC Public Health* 2022; **22**: 601.

117 Esposito E, Szypulska A. Addressing intimate-partner violence during COVID-19 in the EU: challenges, responses and areas of improvement. *Journal of Gender-Based Violence* 2022; **6**: 492–500.

118 Evans DP, Hawk SR, Ripkey CE. Domestic Violence in Atlanta, Georgia Before and During COVID-19. *Violence Gend* 2021; **8**: 140–7.

119 Evcili F, Demirel G. From the perspective of Turkish women: intimate partner violence and perceived stress level in the Covid-19 pandemic. *Women Health* 2022; **62**: 108–16.

120 Fedina L, Peitzmeier SM, Ward MR, Ashwell L, Tolman R, Herrenkohl TI. Associations between intimate partner violence and increased economic insecurity among women and transgender adults during the COVID-19 pandemic. *Psychol Violence* 2023; **13**: 53–63.

121 Fereidooni R, Mootz J, Sabaei R, *et al.* The COVID-19 Pandemic, Socioeconomic Effects, and Intimate Partner Violence Against Women: A Population-Based Cohort Study in 2020, Iran. *Am J Public Health* 2023; **113**: 228–37.

122 Ferragina F, Barca I, Sorrentino A, *et al.* Effect of COVID-19 Italian Lockdown on Maxillofacial Trauma Related to Domestic Violence: A Retrospective Cohort Study. *Life* 2022; **12**: 1463.

123 Fetene G, Alie MS, Girma D, Negesse Y. Prevalence and its predictors of intimate partner violence against pregnant women amid COVID-19 pandemic in Southwest Ethiopia, 2021: A cross-sectional study. *SAGE Open Med* 2022; **10**: 205031212210793.

124 Fianu A, Aissaoui H, Naty N, *et al.* Health Impacts of the COVID-19 Lockdown Measure in a Low Socio-Economic Setting: A Cross-Sectional Study on Reunion Island. *Int J Environ Res Public Health* 2022; **19**: 13932.

125 Fitrianingsih ADR, Saki VY. Assessing Types and Causes of Domestic Violence Against Women during Covid-19 Pandemic in Bandung Urban Slum Areas, Indonesia. *Malaysian Journal of Medicine and Health Sciences* 2022; **18**: 58–66.

126 Fleming CJE, Franzese AT. Should I stay or should I go? Evaluating intimate relationship outcomes during the 2020 pandemic shutdown. *Couple and Family Psychology: Research and Practice* 2021; **10**: 158–67.

127 Focardi M, Grassi S, Raddi S, *et al.* Trend in 167 cases of minors witnessing violence: The role played by COVID-19 pandemic. *Front Pediatr* 2022; **10**. DOI:10.3389/fped.2022.949922.

128 Fogarty A, Savopoulos P, Seymour M, *et al.* Providing therapeutic services to women and children who have experienced intimate partner violence during the COVID-19 pandemic: Challenges and learnings. *Child Abuse Negl* 2022; **130**: 105365.

129 Folayan MO, Arije O, Enemo A, *et al.* Associations between COVID-19 vaccine hesitancy and the experience of violence among women and girls living with and at risk of HIV in Nigeria. *African Journal of AIDS Research* 2022; **21**: 306–16.

130 Forry J Ben, Kirabira J, Akimana B, *et al.* Gender-based violence and its determinants during the COVID-19 lockdown in a low-income country: a cross-sectional survey. *Journal of Gender-Based Violence* 2022; **6**: 1–19.

131 Gama A, Pedro AR, de Carvalho MJL, *et al.* Domestic Violence during the COVID-19 Pandemic in Portugal. *Portuguese Journal of Public Health* 2020; **38**: 32–40.

132 García-Fernández L, Romero-Ferreiro V, Padilla S, David López-Roldán P, Monzó-García M, Rodriguez-Jimenez R. Gender differences in emotional response to the COVID-19 outbreak in Spain. *Brain Behav* 2021; **11**. DOI:10.1002/brb3.1934.

133 García-Zamora S, Pulido L, Miranda-Arboleda AF, *et al.* Aggression, Micro-aggression, and Abuse Against Health Care Providers During the COVID-19 Pandemic. A Latin American Survey. *Curr Probl Cardiol* 2022; **47**. DOI:10.1016/j.cpcardiol.2022.101296.

134 Gassó AM, Mueller-Johnson K, Agustina JR, Gómez-Durán EL. Exploring Sexting and Online Sexual Victimization during the COVID-19 Pandemic Lockdown. *Int J Environ Res Public Health* 2021; **18**: 6662.

135 Gebrewahd GT, Gebremeskel GG, Tadesse DB. Intimate partner violence against reproductive age women during COVID-19 pandemic in northern Ethiopia 2020: a community-based cross-sectional study. *Reprod Health* 2020; **17**: 152.

136 Ghimire C, Acharya S, Shrestha C, KC P, Singh S, Sharma P. Interpersonal Violence during the COVID-19 Lockdown Period in Nepal: A Descriptive Cross-sectional Study. *Journal of Nepal Medical Association* 2020; **58**: 751–7.

137 Gilchrist G, Potts LC, Connolly DJ, *et al.* Experience and perpetration of intimate partner violence and abuse by gender of respondent and their current partner before and during COVID-19 restrictions in 2020: a cross-sectional study in 13 countries. *BMC Public Health* 2023; **23**: 316.

138 Gleason N, Banik S, Braverman J, Coleman E. The Impact of the COVID-19 Pandemic on Sexual Behaviors: Findings From a National Survey in the United States. *J Sex Med* 2021; **18**: 1851–62.

139 Glowacz F, Dziewa A, Schmits E. Intimate Partner Violence and Mental Health during Lockdown of the COVID-19 Pandemic. *Int J Environ Res Public Health* 2022; **19**: 2535.

140 Gonçalves Júnior J, Maia MAG, Moreira JL de S, Dias HS, Félix EB, Machado SSF. Domestic violence against Latin American women and female mental health during the new coronavirus (SARS-CoV-2) outbreak. *Int J Inj Contr Saf Promot* 2022; **29**: 193–6.

141 Gosangi B, Park H, Thomas R, *et al.* Exacerbation of Physical Intimate Partner Violence during COVID-19 Pandemic. *Radiology* 2021; **298**: E38–45.

142 Gregory A, Williamson E. ‘I Think it Just Made Everything Very Much More Intense’: A Qualitative Secondary Analysis Exploring The Role Of Friends and Family Providing Support to Survivors of Domestic Abuse During The COVID-19 Pandemic. *J Fam Violence* 2022; **37**: 991–1004.

143 Gulesci S, Puente–Beccar M, Ubfal D. Can youth empowerment programs reduce violence against girls during the COVID-19 pandemic? *J Dev Econ* 2021; **153**: 102716.

144 Haag H (Lin), Toccalino D, Estrella MJ, Moore A, Colantonio A. The Shadow Pandemic: A Qualitative Exploration of the Impacts of COVID-19 on Service Providers and Women

Survivors of Intimate Partner Violence and Brain Injury. *Journal of Head Trauma Rehabilitation* 2022; **37**: 43–52.

145 Haddad C, Malhab SB, Sacre H, *et al.* Factors related to pregnancy status and unwanted pregnancy among lebanese women during the COVID-19 lockdown: a cross-sectional study. *Archives of Public Health* 2022; **80**: 68.

146 Halperin O, Ali-Saleh O, Ore L, Jadaon JE. Depression, Stress and the Mediating Role of Intimate Partner Violence (IPV) Among Israeli Women of Childbearing Age in the Shadow of the COVID-19 Pandemic. *J Interpers Violence* 2023; **38**: 3586–611.

147 Hamadani JD, Hasan MI, Baldi AJ, *et al.* Immediate impact of stay-at-home orders to control COVID-19 transmission on socioeconomic conditions, food insecurity, mental health, and intimate partner violence in Bangladeshi women and their families: an interrupted time series. *Lancet Glob Health* 2020; **8**: e1380–9.

148 Hamamra B. Domestic Violence in Palestine during the Outbreak of COVID-19: A Qualitative Content Analysis. *Women Crim Justice* 2022; : 1–12.

149 Hamzaoglu N, Rozant-Reisyan R, Kalfoglou S. The Evaluation of Depression Levels and Domestic Violence during the COVID-19 Pandemic. *Soc Work Public Health* 2023; **38**: 47–57.

150 Haq W, Raza SH, Mahmood T. The pandemic paradox: domestic violence and happiness of women. *PeerJ* 2020; **8**: e10472.

151 Hassan R, Sanders T, Gichuna S, Campbell R, Mutonyi M, Mwangi P. Informal settlements, Covid-19 and sex workers in Kenya. *Urban Studies* 2023; **60**: 1483–96.

152 Hassoun Ayoub L, Partridge T, Gómez JM. Two sides of the same coin: A mixed methods study of Black mothers' experiences with violence, stressors, parenting, and coping during the COVID-19 pandemic. *Journal of Social Issues* 2023; **79**: 667–93.

153 Heck CJ, Theodore DA, Sovic B, *et al.* Correlates of psychological distress among undergraduate women engaged in remote learning through a New York City college during the COVID-19 pandemic. *Journal of American College Health* 2023; : 1–10.

154 Helito AC, Lindoso L, Sieczkowska SM, *et al.* Poor Sleep quality and health-related quality of life impact in adolescents with and without chronic immunosuppressive conditions during COVID-19 quarantine. *Clinics* 2021; **76**: e3501.

155 Heward-Belle S, Lovell RC, Jones J, Tucker H, Melander N. Practice in a Time of Uncertainty: Practitioner Reflections on Working With Families Experiencing Intimate Partner Violence During the COVID-19 Global Pandemic. *Affilia* 2022; **37**: 605–23.

156 Hildersley R, Easter A, Bakolis I, Carson L, Howard LM. Changes in the identification and management of mental health and domestic abuse among pregnant women during the COVID-19 lockdown: regression discontinuity study. *BJPsych Open* 2022; **8**: e96.

157 Wong JYH, Luk LYF, Yip TF, Lee TTL, Wai AKC, Ho JWK. Incidence of Emergency Department Visits for Sexual Abuse Among Youth in Hong Kong Before and During the COVID-19 Pandemic. *JAMA Netw Open* 2022; **5**: e2236278.

158 Hoehn-Velasco L, Silverio-Murillo A, de la Miyar JRB. The great crime recovery: Crimes against women during, and after, the COVID-19 lockdown in Mexico. *Econ Hum Biol* 2021; **41**: 100991.

159 Holland KM, Jones C, Vivolo-Kantor AM, *et al.* Trends in US Emergency Department Visits for Mental Health, Overdose, and Violence Outcomes Before and During the COVID-19 Pandemic. *JAMA Psychiatry* 2021; **78**: 372.

160 Hossain Md, Asadullah Md, Rahaman A, *et al.* Prediction on Domestic Violence in Bangladesh during the COVID-19 Outbreak Using Machine Learning Methods. *Applied System Innovation* 2021; **4**: 77.

161 Howard KJ, Leong C, Chambless S, *et al.* Major Depression in Postpartum Women during the COVID-19 Pandemic: Can Social Support Buffer Psychosocial Risks and Substance Use? *Int J Environ Res Public Health* 2022; **19**: 15748.

162 Huiskes P, Dinis MAP, Caridade S. Technology-Facilitated Sexual Violence Victimization during the COVID-19 Pandemic: Behaviors and Attitudes. *J Aggress Maltreat Trauma* 2022; **31**: 1148–67.

163 Huq M, Das T, Devakumar D, Daruwalla N, Osrin D. Intersectional tension: a qualitative study of the effects of the COVID-19 response on survivors of violence against women in urban India. *BMJ Open* 2021; **11**: e050381.

164 Hurley KJ, Morganson VJ. An empirical examination of sexual harassment and Stockholm syndrome in relation to essential and non-essential workers during the COVID-19 pandemic. *Journal of Sexual Aggression* 2023; **29**: 243–55.

165 Huscsava MM, Scharinger C, Plener PL, Kothgassner OD. 'The world somehow stopped moving': impact of the COVID-19 pandemic on adolescent psychiatric outpatients and the implementation of teletherapy. *Child Adolesc Ment Health* 2022; **27**: 232–7.

166 Indu PV, Vijayan B, Tharayil HM, Ayirolimeethal A, Vidyadharan V. Domestic violence and psychological problems in married women during COVID-19 pandemic and lockdown: A community-based survey. *Asian J Psychiatr* 2021; **64**: 102812.

167 Iob E, Frank P, Steptoe A, Fancourt D. Levels of Severity of Depressive Symptoms Among At-Risk Groups in the UK During the COVID-19 Pandemic. *JAMA Netw Open* 2020; **3**: e2026064.

168 Iverson KM, Dardis CM, Cowlishaw S, *et al.* Effects of Intimate Partner Violence During COVID-19 and Pandemic-Related Stress on the Mental and Physical Health of Women Veterans. *J Gen Intern Med* 2022; **37**: 724–33.

169 Jiang M, Shao X, Rao S, *et al.* Emotional State of Chinese Healthcare Workers During COVID-19 Pandemic. *Front Psychol* 2022; **13**: 854815.

170 Johnson L. Exploring factors associated with pregnant women's experiences of material hardship during COVID-19: a cross-sectional Qualtrics survey in the United States. *BMC Pregnancy Childbirth* 2021; **21**: 755.

171 Johnson E, Jenssen S, Wernette GT, Tweel T, Johnson D, Zlotnick C. Web-based intervention to reduce intimate partner violence during perinatal period: A modified protocol in response to the COVID-19 pandemic. *Psychiatry Res* 2022; **317**: 114895.

172 Johnston M, Davies SE, True J, Riveros-Morales Y. "Patriarchal reset" in the asia pacific during COVID-19: the impacts on women's security and rights. *The Pacific Review* 2023; **36**: 603–30.

173 Joseph J, Canlas RP. The impact of posttraumatic stress symptoms on quality of life among battered wives in India amidst the COVID-19 pandemic. *Heliyon* 2023; **9**: e12894.

174 Ike TJ, Jidong DE, Ayobi EE. Women's perceptions of domestic, intimate partner violence and the government's interventions in Nigeria: A qualitative study. *Criminology & Criminal Justice* 2022; : 174889582211289.

175 Jung S, Kneer J, Krüger THC. Mental Health, Sense of Coherence, and Interpersonal Violence during the COVID-19 Pandemic Lockdown in Germany. *J Clin Med* 2020; **9**: 3708.

176 Kachi Y, Fujiwara T, Eguchi H, *et al.* Association between maternity harassment and depression during pregnancy amid the COVID-19 state of emergency. *J Occup Health* 2021; **63**. DOI:10.1002/1348-9585.12196.

177 Kadra-Scalzo G, Kornblum D, Stewart R, Howard LM. Adverse outcomes associated with recorded victimization in mental health electronic records during the first UK COVID-19 lockdown. *Soc Psychiatry Psychiatr Epidemiol* 2023; **58**: 431–40.

178 Kamali K, Maleki A, Yazdi SAB, *et al.* The prevalence of violence and its association with mental health among the Iranian population in one year after the outbreak of COVID-19 disease. *BMC Psychiatry* 2023; **23**: 33.

179 Kamath A, Yadav A, Baghel J, Mundle S. Locked Down: Experiences of Domestic Violence in Central India. *Glob Health Sci Pract* 2022; **10**: e2100630.

180 Kamoshida S, Nihonmatsu N, Takagi G, Wakashima K. The relationship between family variables and family social problems during the COVID-19 pandemic. *PLoS One* 2022; **17**: e0270210.

181 Karakasi M-V, Voultsos P, Fotou E, *et al.* Emerging trends in domestic homicide/femicide in Greece over the period 2010–2021. *Med Sci Law* 2023; **63**: 120–31.

182 Karp C, Moreau C, Sheehy G, *et al.* Youth Relationships in the Era of COVID-19: A Mixed-Methods Study Among Adolescent Girls and Young Women in Kenya. *Journal of Adolescent Health* 2021; **69**: 754–61.

183 Kassie A, Handebo S, Adugna A, Shitu K. Violence against girls during COVID-19 pandemic and associated factors in Gondar city, North West Ethiopia. *Epidemiol Infect* 2022; **150**: e31.

184 Kaswa R. The impact of the COVID-19 pandemic on healthcare service access for the victims of sexual assault. *South African Family Practice* 2021; **63**. DOI:10.4102/safp.v63i1.5367.

185 Katana E, Amodan BO, Bulage L, *et al.* Violence and discrimination among Ugandan residents during the COVID-19 lockdown. *BMC Public Health* 2021; **21**: 467.

186 Kaur S. Gendered Impact of the COVID-19: Insights from an Interpretative Phenomenological Perspective. *Vikalpa: The Journal for Decision Makers* 2022; **47**: 91–105.

187 Kliem S, von Thadden A, Lohmann A, Kröger C, Baier D. The Effect of the Covid-19 Pandemic on Domestic Violence in Germany: A Comparison of Three Representative Population Surveys. *J Interpers Violence* 2023; **38**: 7296–314.

188 Koly KN, Tasnim Z, Ahmed S, *et al.* Mental healthcare-seeking behavior of women in Bangladesh: content analysis of a social media platform. *BMC Psychiatry* 2022; **22**: 797.

189 Krause KH, DeGue S, Kilmer G, Niolon PH. Prevalence and Correlates of Non-Dating Sexual Violence, Sexual Dating Violence, and Physical Dating Violence Victimization among U.S.

High School Students during the COVID-19 Pandemic: Adolescent Behaviors and Experiences Survey, United States, 2021. *J Interpers Violence* 2023; **38**: 6961–84.

190 Krause KH, Verlenden J V., Szucs LE, *et al.* Disruptions to School and Home Life Among High School Students During the COVID-19 Pandemic — Adolescent Behaviors and Experiences Survey, United States, January–June 2021. *MMWR Suppl* 2022; **71**: 28–34.

191 Krishnamurti T, Davis AL, Quinn B, Castillo AF, Martin KL, Simhan HN. Mobile Remote Monitoring of Intimate Partner Violence Among Pregnant Patients During the COVID-19 Shelter-In-Place Order: Quality Improvement Pilot Study. *J Med Internet Res* 2021; **23**: e22790.

192 Kuhlmann E, Bruns L, Hoeper K, *et al.* Work situation of rheumatologists and residents in times of COVID-19. *Z Rheumatol* 2023; **82**: 331–41.

193 Kulik L, Ramon D. The relationship between family-work conflict and spousal aggression during the COVID-19 pandemic. *Community Work Fam* 2022; **25**: 240–59.

194 Kulik L. Family-Work Conflict and Spousal Aggression: A Comparative Analysis Pre- and Peri- COVID-19 Pandemic. *J Aggress Maltreat Trauma* 2023; **32**: 688–708.

195 Lafta R, Qusay N, Mary M, Burnham G. Violence against doctors in Iraq during the time of COVID-19. *PLoS One* 2021; **16**: e0254401.

196 Lamichhane A, Rana S, Shrestha K, *et al.* Violence and sexual and reproductive health service disruption among girls and young women during COVID-19 pandemic in Nepal: A cross-sectional study using interactive voice response survey. *PLoS One* 2021; **16**: e0260435.

197 Lampe A, Daniels JK, Trawöger I, Beck T, Riedl D. Did domestic violence really increase in the early phase of the COVID-19 pandemic? Results of an interview-based observational study. *Z Psychosom Med Psychother* 2021; **67**: 303–14.

198 Langhinrichsen-Rohling J, Schroeder GE, Langhinrichsen-Rohling RA, *et al.* Couple Conflict and Intimate Partner Violence during the Early Lockdown of the Pandemic: The Good, the Bad, or Is It Just the Same in a North Carolina, Low-Resource Population? *Int J Environ Res Public Health* 2022; **19**: 2608.

199 Lazzerini M, Covi B, Mariani I, *et al.* Quality of facility-based maternal and newborn care around the time of childbirth during the COVID-19 pandemic: online survey investigating maternal perspectives in 12 countries of the WHO European Region. *The Lancet Regional Health - Europe* 2022; **13**: 100268.

200 Lee A, Rivera C, Bunge Montes S, *et al.* Identifying Prevalence and Risk Factors for Intimate Partner Violence in Pregnant Women in Rural Guatemala. *J Womens Health Dev* 2021; **04**: 113–22.

201 Leikuma-Rimicane L, Ceballos RF, Medina MND. Location and Type of Crimes in The Philippines: Insights for Crime Prevention and Management. *International Journal of Criminal Justice Sciences* 2022; **17**: 22–33.

202 Leon-Larios F, Silva Reus I, Lahoz Pascual I, *et al.* Women's Access to Sexual and Reproductive Health Services during Confinement Due to the COVID-19 Pandemic in Spain. *J Clin Med* 2022; **11**: 4074.

203 Leske S, Kõlves K, Crompton D, Arensman E, de Leo D. Real-time suicide mortality data from police reports in Queensland, Australia, during the COVID-19 pandemic: an interrupted time-series analysis. *Lancet Psychiatry* 2021; **8**: 58–63.

204 Lipp NS, Johnson NL. The impact of COVID-19 on domestic violence agency functioning: A case study. *Journal of Social Issues* 2023; **79**: 735–46.

205 Liu R, Li Y, An Y, *et al.* Workplace violence against frontline clinicians in emergency departments during the COVID-19 pandemic. *PeerJ* 2021; **9**: e12459.

206 Logie CH, Okumu M, Latif M, *et al.* Exploring resource scarcity and contextual influences on wellbeing among young refugees in Bidi Bidi refugee settlement, Uganda: findings from a qualitative study. *Confl Health* 2021; **15**: 3.

207 Long M, Huang J, Peng Y, Mai Y, Yuan X, Yang X. The Short- and Long-Term Impact of COVID-19 Lockdown on Child Maltreatment. *Int J Environ Res Public Health* 2022; **19**: 3350.

208 Luebke J, Kako P, Lopez A, *et al.* Barriers Faced by American Indian Women in Urban Wisconsin in Seeking Help Following an Experience of Intimate Partner Violence. *Violence Against Women* 2023; **29**: 2080–103.

209 Lyons M, Brewer G. Experiences of Intimate Partner Violence during Lockdown and the COVID-19 Pandemic. *J Fam Violence* 2022; **37**: 969–77.

210 MacGregor JCD, Burd C, Mantler T, *et al.* Experiences of Women Accessing Violence Against Women Outreach Services in Canada During the COVID-19 Pandemic: a Brief Report. *J Fam Violence* 2023; **38**: 997–1005.

211 Maftei A, Dănilă O. Give me your password! What are you hiding? Associated factors of intimate partner violence through technological abuse. *Current Psychology* 2023; **42**: 8781–97.

212 Mahamid F, Veronese G, Bdier D. Gender-based violence experiences among Palestinian women during the COVID-19 pandemic: mental health professionals' perceptions and concerns. *Confl Health* 2022; **16**: 13.

213 Mahapatro M, Prasad MM, Singh SP. Role of Social Support in Women facing Domestic Violence during Lockdown of Covid-19 while Cohabiting with the Abusers: Analysis of Cases Registered with the Family Counseling Centre, Alwar, India. *J Fam Issues* 2021; **42**: 2609–24.

214 Mahapatro M, Prasad MM, Singh SP. Domestic Violence and Covid-19: Policy and Pattern Analysis of Reported Cases at the Family Counseling Center (FCC) in Alwar, India. *Sexuality Research and Social Policy* 2023; **20**: 1096–104.

215 Maharlouei N, Roozmeh S, Zahed Roozegar M, *et al.* Intimate partner violence during pregnancy in COVID-19 pandemic: a cross-sectional study from South-west of Iran. *BMC Public Health* 2023; **23**: 325.

216 Mahlangu P, Gibbs A, Shai N, Machisa M, Nunze N, Sikweyiya Y. Impact of COVID-19 lockdown and link to women and children's experiences of violence in the home in South Africa. *BMC Public Health* 2022; **22**: 1029.

217 Mahmood KI, Shabu SA, M-Amen KM, *et al.* The Impact of COVID-19 Related Lockdown on the Prevalence of Spousal Violence Against Women in Kurdistan Region of Iraq. *J Interpers Violence* 2022; **37**: NP11811–35.

218 Mahmud M, Riley E. Household response to an extreme shock: Evidence on the immediate impact of the Covid-19 lockdown on economic outcomes and well-being in rural Uganda. *World Dev* 2021; **140**: 105318.

219 Maji S, Bansod S, Singh T. Domestic violence during COVID-19 pandemic: The case for Indian women. *J Community Appl Soc Psychol* 2022; **32**: 374–81.

220 Malkawi SH, Almhdawi K, Jaber AF, Alqatarneh NS. COVID-19 Quarantine-Related Mental Health Symptoms and their Correlates among Mothers: A Cross Sectional Study. *Matern Child Health J* 2021; **25**: 695–705.

221 Mantell JE, Franks J, Lahuerta M, *et al.* Life in the Balance: Young Female Sex Workers in Kenya Weigh the Risks of COVID-19 and HIV. *AIDS Behav* 2021; **25**: 1323–30.

222 Mantler T, Shillington KJ, Yates J, Tryphonopoulos P, Jackson KT, Ford-Gilboe M. Resilience is more than Nature: An Exploration of the Conditions that Nurture Resilience Among Rural Women who have Experienced IPV. *J Fam Violence* 2022; : 1–11.

223 Mantler T, Shillington KJ, Davidson CA, *et al.* Impacts of COVID-19 on the Coping Behaviours of Canadian Women Experiencing Intimate Partner Violence. *Global Social Welfare* 2022; **9**: 141–56.

224 Manzoor MA, Hassan S-U, Muazzam A, Tuarob S, Nawaz R. Social mining for sustainable cities: thematic study of gender-based violence coverage in news articles and domestic violence in relation to COVID-19. *J Ambient Intell Humaniz Comput* 2022; : 1–12.

225 Marchant AD, Gray S, Ludwig DC, Dillon J. What Is the Effect of COVID-19 Social Distancing on Oral and Maxillofacial Trauma Related to Domestic Violence? *Journal of Oral and Maxillofacial Surgery* 2021; **79**: 2319.e1-2319.e8.

226 Marsh SM, Rocheleau CM, Carbone EG, Hartley D, Reichard AA, Tiesman HM. Occurrences of Workplace Violence Related to the COVID-19 Pandemic, United States, March 2020 to August 2021. *Int J Environ Res Public Health* 2022; **19**: 14387.

227 Matose T, Maviza G, Nunu WN. Pervasive irregular migration and the vulnerabilities of irregular female migrants at Plumtree border post in Zimbabwe. *J Migr Health* 2022; **5**: 100091.

228 McCool-Myers M, Kozlowski D, Jean V, Cordes S, Gold H, Goedken P. The COVID-19 pandemic's impact on sexual and reproductive health in Georgia, USA: An exploration of behaviors, contraceptive care, and partner abuse. *Contraception* 2022; **113**: 30–6.

229 McCool-Myers M, Grasso D, Kozlowski D, *et al.* The COVID-19 pandemic's intersectional impact on work life, home life and wellbeing: an exploratory mixed-methods analysis of Georgia women's experiences during the pandemic. *BMC Public Health* 2022; **22**: 1988.

230 McDougal L, Erikat J, Yusufi H, Sahid R, Streuli S, Fielding-Miller R. Understanding the impact of the COVID-19 pandemic on refugee communities in San Diego, California: A participatory action research cross-sectional study. *SSM Popul Health* 2022; **18**: 101110.

231 McKinlay AR, Simon YR, May T, Fancourt D, Burton A. How did UK social distancing restrictions affect the lives of women experiencing intimate partner violence during the COVID-19 pandemic? A qualitative exploration of survivor views. *BMC Public Health* 2023; **23**: 123.

232 McMillan IF, Armstrong LM, Langhinrichsen-Rohling J. Transitioning to parenthood during the pandemic: COVID-19 related stressors and first-time expectant mothers' mental health. *Couple and Family Psychology: Research and Practice* 2021; **10**: 179–89.

233 Abu-Rmeileh NM, Wahdan Y, Mehrtash H, Hamad KA, Awad A, Tunçalp Özge. Exploring women's experiences during childbirth in health facilities during COVID-19 pandemic in occupied palestinian territory: a cross-sectional community survey. *BMC Pregnancy Childbirth* 2022; **22**: 957.

234 Melaku TM, Beeman A. Navigating white academe during crisis: The impact of COVID-19 and racial violence on women of color professionals. *Gend Work Organ* 2023; **30**: 673–91.

235 Mena-Tudela D, Iglesias-Casas S, Cervera-Gasch A, Andreu-Pejó L, González-Chordá VM, Valero-Chillerón MJ. Breastfeeding and Obstetric Violence during the SARS-CoV-2 Pandemic in Spain: Maternal Perceptions. *Int J Environ Res Public Health* 2022; **19**: 15737.

236 Merken S, Slakoff DC, Aujla W, Moton L. Navigating Biases and Distrust of Systems: American and Canadian Intimate Partner Violence Service Providers' Experiences with Trans and Immigrant Women Clients. *Vict Offender* 2023; **18**: 141–68.

237 Miller AP, Mugamba S, Bulamba RM, *et al.* Exploring the impact of COVID-19 on women's alcohol use, mental health, and experiences of intimate partner violence in Wakiso, Uganda. *PLoS One* 2022; **17**: e0263827.

238 Mingude AB, Dejene TM. Prevalence and associated factors of gender based violence among Baso high school female students, 2020. *Reprod Health* 2021; **18**: 247.

239 Moawad AM, El Desouky ED, Salem MR, Elhawary AS, Hussein SM, Hassan FM. Violence and sociodemographic related factors among a sample of Egyptian women during the COVID-19 pandemic. *Egypt J Forensic Sci* 2021; **11**: 29.

240 Moore G, Buckley K, Howarth E, *et al.* Police referrals for domestic abuse before and during the first COVID-19 lockdown: An analysis of routine data from one specialist service in South Wales. *J Public Health (Bangkok)* 2022; **44**: e252–9.

241 Morales Chainé S, López Montoya A, Bosch Maldonado A, *et al.* Mental Health Symptoms, Binge Drinking, and the Experience of Abuse During the COVID-19 Lockdown in Mexico. *Front Public Health* 2021; **9**. DOI:10.3389/fpubh.2021.656036.

242 Morales Chainé S, Robles García R, Bosch A, Treviño Santa Cruz CL. Depressive, Anxious, and Post-Traumatic Stress Symptoms Related to Violence during the COVID-19 Pandemic, by Sex, COVID-19 Status, and Intervention-Seeking Conditions among the General Population. *Int J Environ Res Public Health* 2022; **19**: 12559.

243 Morgan A, Boxall H, Payne JL. Reporting to police by intimate partner violence victim-survivors during the COVID-19 pandemic. *Journal of Criminology* 2022; **55**: 285–305.

244 Morley C, Carrington K, Ryan V, *et al.* Locked Down with the Perpetrator: The Hidden Impacts of COVID-19 on Domestic and Family Violence in Australia. *International Journal for Crime, Justice and Social Democracy* 2021; **10**: 204–22.

245 Moro MF, Calamandrei G, Poli R, *et al.* The Impact of the COVID-19 Pandemic on the Mental Health of Healthcare Workers in Italy: Analyzing the Role of Individual and Workplace-Level Factors in the Reopening Phase After Lockdown. *Front Psychiatry* 2022; **13**. DOI:10.3389/fpsyt.2022.867080.

246 Moss SE, Mahmoudi M. STEM the bullying: An empirical investigation of abusive supervision in academic science. *EClinicalMedicine* 2021; **40**: 101121.

247 Moulin F, Jean F, Melchior M, *et al.* Longitudinal impact of the COVID19 pandemic on mental health in a general population sample in France: Evidence from the COMET Study. *J Affect Disord* 2023; **320**: 275–83.

248 Mubaiwa O, Bradley T, Meme J. The gendered impact of COVID-19 on FGM. *Dev Pract* 2022. DOI:10.1080/09614524.2022.2093331.

249 Muldoon KA, Denize KM, Talarico R, *et al.* COVID-19 pandemic and violence: rising risks and decreasing urgent care-seeking for sexual assault and domestic violence survivors. *BMC Med* 2021; **19**: 20.

250 Muldoon KA, Denize KM, Talarico R, *et al.* COVID-19 and perinatal intimate partner violence: a cross-sectional survey of pregnant and postpartum individuals in the early stages of the COVID-19 pandemic. *BMJ Open* 2021; **11**: e049295.

251 Muldoon KA, Talarico R, Fell DB, Illingworth H, Sampsel K, Manuel DG. Population-Level Trends in Emergency Department Encounters for Sexual Assault Preceding and During the COVID-19 Pandemic Across Ontario, Canada. *JAMA Netw Open* 2022; **5**: e2248972.

252 Muñoz del Carpio-Toia A, Begazo Muñoz del Carpio L, Mayta-Tristan P, Alarcón-Yaquetto DE, Málaga G. Workplace Violence Against Physicians Treating COVID-19 Patients in Peru: A Cross-Sectional Study. *The Joint Commission Journal on Quality and Patient Safety* 2021; **47**: 637–45.

253 Musse JLL, Musse FCC, Pelloso SM, Carvalho MD de B. Violence against health personnel before and during the COVID-19 pandemic. *Rev Assoc Med Bras* 2022; **68**: 1524–9.

254 Mutambara VM, Crankshaw TL, Freedman J. Assessing the Impacts of COVID-19 on Women Refugees in South Africa. *J Refug Stud* 2022; **35**: 704–21.

255 Muthulingam T, Edirisinghe PAS, Wijewardhane HP, *et al.* A Study on Victims of Intimate Partner Violence Reported to Colombo North Teaching Hospital, Sri Lanka During 2019-2021. *Acad Forensic Pathol* 2022; **12**: 95–111.

256 Nagashima-Hayashi M, Durrance-Bagale A, Marzouk M, *et al.* Gender-Based Violence in the Asia-Pacific Region during COVID-19: A Hidden Pandemic behind Closed Doors. *Int J Environ Res Public Health* 2022; **19**: 2239.

257 Nagaswami M V., Yeung A. Intimate Partner Violence Among South Asian Women During the COVID-19 Pandemic: A Cross-Sectional Survey of Prevalence and Risk Factors. *J Immigr Minor Health* 2023; **25**: 854–61.

258 Naghizadeh S, Mirghafourvand M, Mohammadirad R. Domestic violence and its relationship with quality of life in pregnant women during the outbreak of COVID-19 disease. *BMC Pregnancy Childbirth* 2021; **21**: 88.

259 Napier-Raman S, Rattani A, Qaiyum Y, Bose V, Seth R, Raman S. Impact of COVID-19 on the lives of vulnerable young people in New Delhi, India: a mixed method study. *BMJ Paediatr Open* 2021; **5**: e001171.

260 Nardi-Rodríguez A, Paredes-López N. How Can We Increase Neighbors' Intention to Report Intimate Partner Violence Against Women During the Pandemic? *Violence Against Women* 2022; **28**: 2377–97.

261 Nerobkova N, Kim SY, Park E-C, Shin J. Workplace Mistreatment and Health Conditions Prior and during the COVID-19 in South Korea: A Cross-Sectional Study. *Int J Environ Res Public Health* 2022; **19**: 12992.

262 Nesset MB, Gudde CB, Mentzoni GE, Palmstierna T. Intimate partner violence during COVID-19 lockdown in Norway: the increase of police reports. *BMC Public Health* 2021; **21**: 2292.

263 Neufeld MY, Jang H, Caron E, Golz R, Brahmbhatt TS, Sanchez SE. Social Vulnerability and COVID-19: Changes in Trauma Activations at a Safety-Net Hospital. *Journal of Surgical Research* 2022; **276**: 100–9.

264 Nhongo SS, Sklavos A, Lee K, Chan STF, Austin S. The changing face of maxillofacial trauma during the 2020 COVID-19 lockdowns in Melbourne, Australia. *Oral Maxillofac Surg* 2022; **27**: 125–30.

265 Niederkrotenthaler T, Laido Z, Kirchner S, *et al.* Mental health over nine months during the SARS-CoV2 pandemic: Representative cross-sectional survey in twelve waves between April and December 2020 in Austria. *J Affect Disord* 2022; **296**: 49–58.

266 Nishat JF, Shovo T-E-A, Ahammed B, Islam MdA, Rahman MM, Hossain MdT. Mental health status of early married girls during the COVID-19 pandemic: A study in the southwestern region of Bangladesh. *Front Psychiatry* 2023; **13**: 1074208.

267 Nittari G, Sagaro GG, Feola A, Scipioni M, Ricci G, Sirignano A. First Surveillance of Violence against Women during COVID-19 Lockdown: Experience from "Niguarda" Hospital in Milan, Italy. *Int J Environ Res Public Health* 2021; **18**: 3801.

268 Nyashanu M, Karonga T, North G, Mguni M, Nyashanu W. COVID-19 lockdown and mental health: Exploring triggers of mental health distress among women in the Copperbelt province, Zambia. *Int J Ment Health* 2022; **51**: 117–30.

269 Ogunlana MO, Nwosu IB, Fafolahan A, *et al.* Pattern of rape and femicide during COVID-19 lockdown: content and discourse analysis of digital media reports in Nigeria. *J Gend Stud* 2021; : 1–14.

270 O'Hara CA, Tan RKJ. Intimate partner violence before and during the COVID-19 lockdown: findings from a cross-sectional study in Singapore. *Sex Health* 2022; **19**: 192–201.

271 Ohliger E, Umpierrez E, Buehler L, *et al.* Mental health of orthopaedic trauma patients during the 2020 COVID-19 pandemic. *Int Orthop* 2020; **44**: 1921–5.

272 Okunola TO, Olofinbiyi BA, Aduloju OP, *et al.* Preliminary report of sexual assaults at Ekiti Sexual Assault Referral Centre, Ado-Ekiti, Southwest, Nigeria (Moremi Clinic). *Trop Doct* 2022; **52**: 79–83.

273 Olding J, Zisman S, Olding C, Fan K. Penetrating trauma during a global pandemic: Changing patterns in interpersonal violence, self-harm and domestic violence in the Covid-19 outbreak. *The Surgeon* 2021; **19**: e9–13.

274 Oloniniyi IO, Ibigbami O, Oginni OA, *et al.* Prevalence and pattern of intimate partner violence during COVID-19 pandemic among Nigerian adults. *Psychol Trauma* 2023; **15**: 868–76.

275 Oswald DL, Kaugars AS, Tait M. American Women's Experiences With Intimate Partner Violence during the Start of the COVID-19 Pandemic: Risk Factors and Mental Health Implications. *Violence Against Women* 2023; **29**: 1419–40.

276 Ottosson A, Draru J, Mwanzi L, *et al.* Applying the iDARE Methodology in Uganda, Kenya, and Tanzania to Improve Health Outcomes During the COVID-19 Pandemic. *Glob Health Sci Pract* 2022; **10**: e2100623.

277 Owusu-Addo E, Owusu-Addo SB, Bennor DM, *et al.* Prevalence and determinants of sexual abuse among adolescent girls during the COVID-19 lockdown and school closures in Ghana: A mixed method study. *Child Abuse Negl* 2023; **135**: 105997.

278 Özdamar Ünal G, İşcan G, Ünal O. The occurrence and consequences of violence against healthcare workers in Turkey: before and during the COVID-19 pandemic. *Fam Pract* 2022; **39**: 1001–8.

279 Özümerzifon Y, Ross A, Brinza T, Gibney G, Garber CE. Exploring a Dance/Movement Program on Mental Health and Well-Being in Survivors of Intimate Partner Violence During a Pandemic. *Front Psychiatry* 2022; **13**. DOI:10.3389/fpsyt.2022.887827.

280 Pakenham KI, Landi G, Boccolini G, Furlani A, Grandi S, Tossani E. The moderating roles of psychological flexibility and inflexibility on the mental health impacts of COVID-19 pandemic and lockdown in Italy. *J Contextual Behav Sci* 2020; **17**: 109–18.

281 Pallansch J, Milam C, Ham K, *et al.* Intimate Partner Violence, Sexual Assault, and Child Abuse Resource Utilization During COVID-19. *Western Journal of Emergency Medicine* 2022; **23**: 589–96.

282 Pande R, Mohod SS, V P, Shanbhag S, Kumar NS. Impact of the COVID-19-induced lockdown on the incidence of ocular trauma presenting to a tertiary care hospital. *BMJ Open Ophthalmol* 2022; **7**: e000861.

283 Panovska-Griffiths J, Szilassy E, Johnson M, *et al.* Impact of the first national COVID-19 lockdown on referral of women experiencing domestic violence and abuse in England and Wales. *BMC Public Health* 2022; **22**: 504.

284 Paramasivan K, Sudarsanam N, Vellaichamy S, Norris KK, Subburaj R. Crime registration and distress calls during COVID-19: two sides of the coin. *Policing Soc* 2022; **32**: 1124–45.

285 Pattojoshi A, Sidana A, Garg S, *et al.* Staying home is NOT 'staying safe': A rapid 8-day online survey on spousal violence against women during the COVID-19 lockdown in India. *Psychiatry Clin Neurosci* 2021; **75**: 64–6.

286 Peitzmeier SM, Fedina L, Ashwell L, Herrenkohl TI, Tolman R. Increases in Intimate Partner Violence During COVID-19: Prevalence and Correlates. *J Interpers Violence* 2022; **37**: NP20482–512.

287 Peraud W, Quintard B, Constant A. Factors associated with violence against women following the COVID-19 lockdown in France: Results from a prospective online survey. *PLoS One* 2021; **16**. DOI:10.1371/journal.pone.0257193.

288 Peters MJ, Roffey DM, Lefaivre KA. Effect of orthopaedic resident education on screening for intimate partner violence. *Inj Epidemiol* 2021; **8**: 62.

289 Pfitzner N, Fitz-Gibbon K, Meyer S. Responding to women experiencing domestic and family violence during the COVID-19 pandemic: Exploring experiences and impacts of remote service delivery in Australia. *Child Fam Soc Work* 2022; **27**: 30–40.

290 Pfitzner N, Fitz-Gibbon K, True J. When staying home isn't safe: Australian practitioner experiences of responding to intimate partner violence during COVID-19 restrictions. *Journal of Gender-Based Violence* 2022; **6**: 297–314.

291 Phillimore J, Pertek S, Akyuz S, *et al.* "We are Forgotten": Forced Migration, Sexual and Gender-Based Violence, and Coronavirus Disease-2019. *Violence Against Women* 2022; **28**: 2204–30.

292 Pinchoff J, Austrian K, Rajshekhar N, *et al.* Gendered economic, social and health effects of the COVID-19 pandemic and mitigation policies in Kenya: evidence from a prospective cohort survey in Nairobi informal settlements. *BMJ Open* 2021; **11**: e042749.

293 Pincock K, Jones N, Mitu K, Guglielmi S, Iyasu A. COVID-19, state (in)visibility and structural violence in low- and middle-income countries. *Int Soc Sci J* 2022; **72**: 869–85.

294 Pino EC, Gebo E, Dugan E, Jay J. Trends in Violent Penetrating Injuries During the First Year of the COVID-19 Pandemic. *JAMA Netw Open* 2022; **5**: e2145708.

295 Pisl V, Vevera J, Holas J, Volavka J. Violent behavior and the COVID-19 lockdowns: a nationwide register-based study. *CNS Spectr* 2023; **28**: 450–6.

296 Plášilová L, Hůla M, Krejčová L, Klapilová K. The COVID-19 Pandemic and Intimate Partner Violence against Women in the Czech Republic: Incidence and Associated Factors. *Int J Environ Res Public Health* 2021; **18**: 10502.

297 Porter C, Favara M, Sánchez A, Scott D. The impact of COVID-19 lockdowns on physical domestic violence: Evidence from a list randomization experiment. *SSM Popul Health* 2021; **14**: 100792.

298 Poraz Findik OT, Barin GG, Erdogdu Yildirim AB, Perdahli Fis N. The Impact of the COVID-19 Pandemic on Pediatric Mental Health Emergency. *Turkish Archives of Pediatrics* 2023; **58**: 80–8.

299 Preis H, Mahaffey B, Pati S, Heiselman C, Lobel M. Adverse Perinatal Outcomes Predicted by Prenatal Maternal Stress Among U.S. Women at the COVID-19 Pandemic Onset. *Annals of Behavioral Medicine* 2021; **55**: 179–91.

300 Rahman MZ, Mustari S, Ahmed T, Rahman S. Domestic violence in developing countries: An evidence from Bangladesh. *J Hum Behav Soc Environ* 2023; **33**: 593–603.

301 Raj A, Johns NE, Barker KM, Silverman JG. Time from COVID-19 shutdown, gender-based violence exposure, and mental health outcomes among a state representative sample of California residents. *EClinicalMedicine* 2020; **26**: 100520.

302 Ramirez F, Harris R. "We're Essential Because You're Essential": How Louisiana Sexual Assault and Domestic Violence Organizations Adapted Outreach Efforts and Health Messaging during COVID-19. *Commun Stud* 2022; **73**: 229–44.

303 Ranzani C de M, Silva SC, Hino P, Taminato M, Okuno MFP, Fernandes H. Profile and characteristics of violence against older adults during the COVID-19 pandemic. *Rev Lat Am Enfermagem* 2023; **31**: e3825.

304 Rashidi Fakari F, Ahmadi Doulabi M, Mokhtaryan-Gilani T, Akbarzadeh Baghban A, Hajian S. A survey of coping strategies and resilience in women victims of domestic violence during the COVID-19 pandemic in Tehran, 2020. *Brain Behav* 2022; **12**: e2730.

305 Ratnasekera AM, Seng SS, Jacovides CL, *et al.* Rising incidence of interpersonal violence in Pennsylvania during COVID-19 stay-at home order. *Surgery* 2022; **171**: 533–40.

306 Rayhan I, Akter K. Prevalence and associated factors of intimate partner violence (IPV) against women in Bangladesh amid COVID-19 pandemic. *Heliyon* 2021; **7**: e06619.

307 Razavi NS, Adeniyi-Ogunyankin G, Basu S, *et al.* Everyday urbanisms in the pandemic city: a feminist comparative study of the gendered experiences of Covid-19 in Southern cities. *Soc Cult Geogr* 2023; **24**: 582–99.

308 Rees S, Mohsin M, Moussa B, *et al.* Cohort profile: intimate partner violence and mental health among women from refugee background and a comparison group of Australian-born – the WATCH cohort study. *BMJ Open* 2022; **12**: e051887.

309 Reiber F, Bryce D, Ulrich R. Self-protecting responses in randomized response designs: A survey on intimate partner violence during the coronavirus disease 2019 pandemic. *Sociol Methods Res* 2022; : 004912412110431.

310 Riggle EDB, Drabble LA, Bochicchio LA, *et al.* Experiences of the COVID-19 pandemic among African American, Latinx, and White sexual minority women: A descriptive phenomenological study. *Psychol Sex Orientat Gend Divers* 2021; **8**: 145–58.

311 Rivera Rivera L, Séris Martínez M, Reynales Shigematsu LM, *et al.* Violence against Women during the COVID-19 Pandemic in Mexico. *Healthcare* 2023; **11**: 419.

312 Rockowitz S, Stevens LM, Rockey JC, *et al.* Patterns of sexual violence against adults and children during the COVID-19 pandemic in Kenya: a prospective cross-sectional study. *BMJ Open* 2021; **11**: e048636.

313 Romahani S, Rahman MM. Prevalence and factors associated with intimate partner violence during Covid-19 pandemic in rural Samarahan, Sarawak. *Journal of Public Health and Development* 2022; **20**: 214–27.

314 Romito P, Pellegrini M, Saurel-Cubizolles M-J. Intimate Partner Violence Against Women During the COVID-19 Lockdown in Italy: A Multicenter Survey Involving Anti-Violence Centers. *Violence Against Women* 2022; **28**: 2186–203.

315 Romito P, Marchand-Martin L, Pellegrini M, Saurel-Cubizolles M-J. Partner's violence during the COVID-19 lockdown and women's fear: a study involving anti-violence centres in Italy. *Journal of Gender-Based Violence* 2022; **6**: 278–96.

316 Roy CM, Bukuluki P, Casey SE, *et al.* Impact of COVID-19 on Gender-Based Violence Prevention and Response Services in Kenya, Uganda, Nigeria, and South Africa: A Cross-Sectional Survey. *Front Glob Womens Health* 2022; **2**: 780771.

317 Royal K. 'Coronavirus has been the perfect excuse for him to just stop paying': child maintenance and economic abuse in the UK during the COVID-19 outbreak. *Journal of Gender-Based Violence* 2022; **6**: 261–77.

318 Runkle JD, Sugg MM, Yadav S, Harden S, Weiser J, Michael K. Real-Time Mental Health Crisis Response in the United States to COVID-19. *Crisis* 2023; **44**: 29–40.

319 Russell KW, Acker SN, Ignacio RC, *et al.* Child physical abuse and COVID-19: Trends from nine pediatric trauma centers. *J Pediatr Surg* 2022; **57**: 297–301.

320 Sabri B, Hartley M, Saha J, Murray S, Glass N, Campbell JC. Effect of COVID-19 pandemic on women's health and safety: A study of immigrant survivors of intimate partner violence. *Health Care Women Int* 2020; **41**: 1294–312.

321 Sacre H, Hajj A, Badro DA, *et al.* The Combined Outcomes of the COVID-19 Pandemic and a Collapsing Economy on Mental Well-Being: A Cross-Sectional Study. *Psychol Rep* 2022; : 003329412211105.

322 Salameh P, Hajj A, Badro DA, Abou Selwan C, Aoun R, Sacre H. Mental Health Outcomes of the COVID-19 Pandemic and a Collapsing Economy: Perspectives from a Developing Country. *Psychiatry Res* 2020; **294**: 113520.

323 Salman A, Al-Ghadban F, Sigodo KO, Taher AK, Chun S. The Psychological and Social Impacts of Curfew during the COVID-19 Outbreak in Kuwait: A Cross-Sectional Study. *Sustainability* 2021; **13**: 8464.

324 Saloniki E-C, Turnpenny A, Collins G, Marchand C, Towers A-M, Hussein S. Abuse and Wellbeing of Long-Term Care Workers in the COVID-19 Era: Evidence from the UK. *Sustainability* 2022; **14**: 9620.

325 Salt E, Wiggins AT, Cooper GL, *et al.* A comparison of child abuse and neglect encounters before and after school closings due to SARS-Cov-2. *Child Abuse Negl* 2021; **118**: 105132.

326 Sánchez OR, Vale DB, Rodrigues L, Surita FG. Violence against women during the COVID-19 pandemic: An integrative review. *International Journal of Gynecology and Obstetrics* 2020; **151**: 180–7.

327 Sanz-Barbero B, Saurina C, Serra L, *et al.* Prevalence and associated factors with sexual violence victimisation youth before, during and after the COVID-19 lockdown: a cross-sectional study in Spain. *BMJ Open* 2021; **11**: e055227.

328 Sanz-Barbero B, Estévez-Garcia F, La Parra-Casado D, Lopez-Ossorio JJ, Vives-Cases C. Intimate partner violence complaints during COVID-19 lockdown in Spain: a cross-sectional and a case–control study. *Eur J Public Health* 2023; **33**: 536–42.

329 Sapire R, Ostrowski J, Maier M, Samari G, Bencomo C, McGovern T. COVID-19 and gender-based violence service provision in the United States. *PLoS One* 2022; **17**: e0263970.

330 Sarı Doğan F, Öztürk TC. The effect of the COVID-19 pandemic on forensic cases admitted to an emergency department. *Forensic Sci Med Pathol* 2022; **19**: 169–74.

331 Schaffer O, Xie F, Cheng D, Grossman SN, Galetta SL, Balcer LJ. Trends in concussion mechanism of injury during the COVID-19 pandemic. *J Neurol Sci* 2023; **445**: 120538.

332 Schokkenbroek JM, Anrijs S, Ponnet K, Hardyns W. Locked Down Together: Determinants of Verbal Partner Violence During the COVID-19 Pandemic. *Violence Gend* 2021; **8**: 148–53.

333 Sediri S, Zgueb Y, Ouanes S, *et al.* Women's mental health: acute impact of COVID-19 pandemic on domestic violence. *Arch Womens Ment Health* 2020; **23**: 749–56.

334 Sefid Fard Jahromi M, Eghbal MH, Rahmanian V. Epidemiology of suicide and suicide attempts in Jahrom district, Southern Iran in light of COVID pandemic: A prospective observational study. *Health Sci Rep* 2022; **5**: e933.

335 Sefidgarbaei F, Mansouri F. The impacts of COVID-19 on the women heading their households in Iran. *J Gend Stud* 2022; **31**: 742–54.

336 Serrano Oswald SE. Gender based political violence against women in Mexico from a regional perspective. *Asia-Pacific Journal of Regional Science* 2023; **7**: 135–57.

337 Serrano-Ibáñez ER, Ramírez-Maestre C, Ruiz-Párraga GT, Esteve R, López-Martínez AE. Pain Interference, Resilience, and Perceived Well-Being During COVID-19: Differences Between Women With and Without Trauma Exposure Prior to the Pandemic. *Int J Public Health* 2022; **67**: 1604443.

338 Shahid E, Fasih U, Taqi U, Jafri AR. An unusual rise in cases of assault in ophthalmic practice during COVID-19 lockdown in a tertiary care hospital. *Oman J Ophthalmol* 2022; **15**: 309–14.

339 Shahjalal Md, Gow J, Alam MM, *et al.* Workplace Violence Among Health Care Professionals in Public and Private Health Facilities in Bangladesh. *Int J Public Health* 2021; **66**. DOI:10.3389/ijph.2021.1604396.

340 Sharma S, Wong D, Schomberg J, *et al.* COVID-19: Differences in sentinel injury and child abuse reporting during a pandemic. *Child Abuse Negl* 2021; **116**: 104990.

341 Sharma P, Khokhar A. Domestic Violence and Coping Strategies Among Married Adults During Lockdown Due to Coronavirus Disease (COVID-19) Pandemic in India: A Cross-Sectional Study. *Disaster Med Public Health Prep* 2022; **16**: 1873–80.

342 Shechory Bitton M. Intimate partner violence in the shadow of COVID-19 and its associations with stress, function and support among the Israeli general population. *Stress and Health* 2023; **39**: 673–83.

343 Shewangzaw Engda A, Dargie Wubetu A, Kasahun Amogne F, Moltot Kitaw T. Intimate partner violence and COVID-19 among reproductive age women: A community-based cross-sectional survey, Ethiopia. *Women's Health* 2022; **18**: 174550652110689.

344 Shillington KJ, Jackson KT, Davidson CA, *et al.* Riding on resilience: impacts of the COVID-19 pandemic on women experiencing intimate partner violence. *SN Social Sciences* 2022; **2**: 92.

345 Shin SY, Choi Y-J. Comparison of Cyberbullying before and after the COVID-19 Pandemic in Korea. *Int J Environ Res Public Health* 2021; **18**: 10085.

346 Shitu S, Yeshaneh A, Abebe H. Intimate partner violence and associated factors among reproductive age women during COVID-19 pandemic in Southern Ethiopia, 2020. *Reprod Health* 2021; **18**: 246.

347 Shoaei NK, Asadi N, Salmani M. The relationship between mental health and violence toward women during the COVID-19 pandemic. *BMC Psychiatry* 2022; **22**: 783.

348 Sigursteinsdottir H, Karlsdottir FB. Does Social Support Matter in the Workplace? Social Support, Job Satisfaction, Bullying and Harassment in the Workplace during COVID-19. *Int J Environ Res Public Health* 2022; **19**: 4724.

349 Sileo KM, Muhumuza C, Helal T, *et al.* Exploring the effects of COVID-19 on family planning: results from a qualitative study in rural Uganda following COVID-19 lockdown. *Reprod Health* 2023; **20**: 31.

350 Singh JK, Duncan TK. Examining the Domestic Violence Crisis in Ventura County in the Wake of the COVID-19 Pandemic. *Am Surg* 2022; : 000313482211115.

351 Smith RN, Nyame-Mireku A, Zeidan A, *et al.* Intimate Partner Violence at a Level-1 Trauma Center During the COVID-19 Pandemic: An Interrupted Time Series Analysis. *Am Surg* 2022; **88**: 1551–3.

352 Soeiro RE, Rocha L, Surita FG, Bahamondes L, Costa ML. A neglected population: Sexual and reproductive issues among adolescent and young Venezuelan migrant women at the northwestern border of Brazil. *International Journal of Gynecology & Obstetrics* 2022; **157**: 51–8.

353 Sorenson SB, Sinko L, Berk RA. The Endemic Amid the Pandemic: Seeking Help for Violence Against Women in the Initial Phases of COVID-19. *J Interpers Violence* 2021; **36**: 4899–915.

354 Sosa Lovera A, Ureña AJ, Arias J, Araujo Rodríguez A, Canario Guzmán JA. Psychological helpline in response to the COVID-19 pandemic in the Dominican Republic. *Couns Psychother Res* 2022; **22**: 534–41.

355 Souma T, Komura K, Arai T, Shimada T, Kanemasa Y. Changes in Collective Efficacy's Preventive Effect on Intimate Partner Violence during the COVID-19 Pandemic. *Int J Environ Res Public Health* 2022; **19**: 12849.

356 Speed A, Thomson C, Richardson K. Stay Home, Stay Safe, Save Lives? An Analysis of the Impact of COVID-19 on the Ability of Victims of Gender-based Violence to Access Justice. *The Journal of Criminal Law* 2020; **84**: 539–72.

357 Stewart SL, Toohey A, Celebre A, Poss JW. Abuse, Mental State, and Health Factors Pre and during the COVID-19 Pandemic: A Comparison among Clinically Referred Adolescents in Ontario, Canada. *Int J Environ Res Public Health* 2021; **18**: 10184.

358 Tadesse AW, Tarekegn SM, Wagaw GB, Muluneh MD, Kassa AM. Prevalence and Associated Factors of Intimate Partner Violence Among Married Women During COVID-19 Pandemic Restrictions: A Community-Based Study. *J Interpers Violence* 2022; **37**: NP8632–50.

359 Teixeira AL, Cerejo D, Rosa M do R, Lisboa M. Effects of the COVID-19 Pandemic on the Lives of Women with Different Socioeconomic Backgrounds and Victimization Experiences in Portugal. *Soc Sci* 2022; **11**: 258.

360 Tesfaw LM, Kassie AB, Flatie BT. Sexual Violence and Other Complications of Corona Virus in Amhara Metropolitan Cities, Ethiopia. *Risk Manag Healthc Policy* 2021; **Volume 14**: 3563–73.

361 Teshome A, Gudu W, Bekele D, Asfaw M, Enyew R, Compton SD. Intimate partner violence among prenatal care attendees amidst the COVID-19 crisis: The incidence in Ethiopia. *International Journal of Gynecology & Obstetrics* 2021; **153**: 45–50.

362 Testoni I, Tredici L, Biancalani G, Bucuţă M, Armezzani M, Orkibi H. Anti-Violence Centers in Italy During the COVID-19 Emergency: Support Strategies for Women Victims of Violence. *OBM Neurobiol* 2022; **06**: 1–19.

363 Tharshini NK, Zamri Hassan, Faizah Haji Mas'ud. Cybercrime Threat Landscape amid the Movement Control Order in Malaysia. *International Journal of Business and Society* 2021; **22**: 1589–601.

364 Thiara RK, Roy S. 'The disparity is evident': COVID-19, violence against women and support for Black and minoritised survivors. *Journal of Gender-Based Violence* 2022; **6**: 315–30.

365 Tierolf B, Geurts E, Steketee M. Domestic violence in families in the Netherlands during the coronavirus crisis: A mixed method study. *Child Abuse Negl* 2021; **116**: 104800.

366 Tiesman H, Marsh S, Konda S, *et al.* Workplace violence during the COVID-19 pandemic: March–October, 2020, United States. *J Safety Res* 2022; **82**: 376–84.

367 Toccalino D, Haag H (Lin), Estrella MJ, *et al.* Addressing the Shadow Pandemic: COVID-19 Related Impacts, Barriers, Needs, and Priorities to Health Care and Support for Women Survivors of Intimate Partner Violence and Brain Injury. *Arch Phys Med Rehabil* 2022; **103**: 1466–76.

368 Todorovic K, O'Leary E, Ward KP, *et al.* Prevalence, increase and predictors of family violence during the COVID-19 pandemic, using modern machine learning approaches. *Front Psychiatry* 2022; **13**. DOI:10.3389/fpsyt.2022.883294.

369 Tosson EE, Saudi RA. Change in spousal violence before and during Covid-19 pandemic in Egypt. *Family Medicine & Primary Care Review* 2021; **23**: 481–7.

370 Tracy BM, Whitson AK, Chen J, Weiss BD, Sims CA. Examining Violence Against Women at a Regional Level 1 Trauma Center During the COVID-19 Pandemic. *Am Surg* 2022; **88**: 404–8.

371 Treglia M, Pallocci M, Passalacqua P, *et al.* Matricide During Covid-19 Pandemic: An Unusual Case of Family Violence. *Romanian Journal of Legal Medicine* 2021; **29**: 272–7.

372 Tripathi P, Dwivedi PS, Sharma S. Psychological impact of domestic violence on women in India due to COVID-19. *Int J Hum Rights Healthc* 2023; **16**: 146–61.

373 Tripathi P, Dwivedi PS, Sharma S. Domestic Violence against Women during the Covid-19: A Case Study of Bihar (India). *J Int Womens Stud* 2022; **24**. https://www.scopus.com/inward/record.uri?eid=2-s2.0-85136704259&partnerID=40&md5=822d51c50717d1b79c2423be03df5e81.

374 Valencia Londoño PA, Nateras González ME, Bruno Solera C, Paz PS. The exacerbation of violence against women as a form of discrimination in the period of the COVID-19 pandemic. *Heliyon* 2021; **7**: e06491.

375 van der Velden PG, Contino C, Das M, Leenen J, Wittmann L. Differences in mental health problems, coping self-efficacy and social support between adults victimised before and adults victimised after the COVID-19 outbreak: population-based prospective study. *The British Journal of Psychiatry* 2022; **220**: 265–71.

376 Vives-Cases C, Parra-Casado D La, Estévez JF, Torrubiano-Domínguez J, Sanz-Barbero B. Intimate Partner Violence against Women during the COVID-19 Lockdown in Spain. *Int J Environ Res Public Health* 2021; **18**: 4698.

377 Vives-Cases C, La Parra-Casado D, Briones-Vozmediano E, *et al.* Coping with intimate partner violence and the COVID-19 lockdown: The perspectives of service professionals in Spain. *PLoS One* 2021; **16**: e0258865.

378 Vu M, Makelarski JA, Winslow VA, *et al.* Racial and Ethnic Disparities in Health-Related Socioeconomic Risks during the Early COVID-19 Pandemic: A National Survey of U.S. Women. *J Womens Health* 2021; **30**: 1375–85.

379 Wada OZ, Olawade DB, Amusa AO, Moses JO, Eteng GJ. Gender-based violence during COVID-19 lockdown: case study of a community in Lagos, Nigeria. *Afr Health Sci* 2022; **22**: 79–87.

380 Walklate S, Godfrey B, Richardson J. Changes and continuities in police responses to domestic abuse in England and Wales during the Covid-19 'lockdown'. *Policing Soc* 2022; **32**: 221–33.

381 Wallace WC, County K, Mason R, Humphrey A. The Trinidad and Tobago Covid-19 Domestic Violence Victimization and Perpetration Study. *J Fam Violence* 2022; : 1–12.

382 Wang W, Lu L, Kelifa MM, *et al.* Mental Health Problems in Chinese Healthcare Workers Exposed to Workplace Violence During the COVID-19 Outbreak: A Cross-Sectional Study Using Propensity Score Matching Analysis. *Risk Manag Healthc Policy* 2020; **Volume 13**: 2827–33.

383 Wang Y, Karver TS, Barrington C, *et al.* Structural and Psychosocial Impacts of the COVID-19 Pandemic on HIV Care and Treatment Outcomes Among Female Sex Workers in the Dominican Republic. *JAIDS Journal of Acquired Immune Deficiency Syndromes* 2022; **89**: 481–8.

384 Warren S, Morley C, Clarke J, *et al.* Weaponizing COVID-19: How the Pandemic Influenced the Behavior of Those Who Use Violence in Domestic and Family Relationships. *Violence Against Women* 2022; **28**: 3415–37.

385 Wathen CN, Burd C, MacGregor JCD, Veenendaal J, McLean I, Mantler T. "We're so limited with what we actually can do if we follow all the rules": a qualitative study of the impact of COVID-19 public health protocols on violence against women services. *BMC Public Health* 2022; **22**: 1175.

386 Wilson CA, Dalton-Locke C, Johnson S, Simpson A, Oram S, Howard LM. Challenges and opportunities of the COVID-19 pandemic for perinatal mental health care: a mixed-methods study of mental health care staff. *Arch Womens Ment Health* 2021; **24**: 749–57.

387 Wirawan GBS, Hanipraja MA, Chrysanta G, *et al.* Anxiety and prior victimization predict online gender-based violence perpetration among Indonesian young adults during COVID-19 pandemic: cross-sectional study. *Egypt J Forensic Sci* 2022; **12**: 31.

388 Wong SMY, Wong CWC, Hui CLM, *et al.* Stressful events as correlates of depressive and PTSD symptoms in Hong Kong women during social unrest and COVID-19 pandemic. *J Affect Disord* 2022; **300**: 263–8.

389 Wood L, Schrag RV, Baumler E, *et al.* On the Front Lines of the COVID-19 Pandemic: Occupational Experiences of the Intimate Partner Violence and Sexual Assault Workforce. *J Interpers Violence* 2022; **37**: NP9345–66.

390 Wood SN, Yirgu R, Wondimagegnehu A, *et al.* Impact of the COVID-19 pandemic on intimate partner violence during pregnancy: evidence from a multimethods study of recently pregnant women in Ethiopia. *BMJ Open* 2022; **12**: e055790.

391 Workman A, Kruger E, Dune T. Policing victims of partner violence during COVID-19: a qualitative content study on Australian grey literature. *Policing Soc* 2021; **31**: 544–64.

392 Wu F, Zhou L, Chen C, *et al.* Association between intimate partner violence and prenatal anxiety and depression in pregnant women: a cross-sectional survey during the COVID-19 epidemic in Shenzhen, China. *BMJ Open* 2022; **12**: e055333.

393 Xavier Hall CD, Javanbakht M, Iyer C, *et al.* Examining the impact of social distancing and methamphetamine use on sexual risk and intimate partner violence in sexual and gender minority young adults during the COVID-19 pandemic. *Drug Alcohol Depend* 2022; **232**: 109231.

394 Xu H, Zeng J, Cao Z, Hao H. The Relationship between Intimate Partner Violence and Online Help-Seeking: A Moderated Mediation Model of Emotion Dysregulation and Perceived Anonymity. *Int J Environ Res Public Health* 2022; **19**: 8330.

395 Xue J, Chen J, Chen C, Hu R, Zhu T. The Hidden Pandemic of Family Violence During COVID-19: Unsupervised Learning of Tweets. *J Med Internet Res* 2020; **22**: e24361.

396 Yamaoka Y, Hosozawa M, Sampei M, *et al.* Abusive and positive parenting behavior in Japan during the COVID-19 pandemic under the state of emergency. *Child Abuse Negl* 2021; **120**: 105212.

397 Yan E, Lai DWL, Lee VWP, Bai X, K. L. Ng H. Abuse and Discrimination Experienced by Older Women in the Era of COVID-19: A Two-Wave Representative Community Survey in Hong Kong. *Violence Against Women* 2022; **28**: 1750–72.

398 Yari A, Zahednezhad H, Gheshlagh RG, Kurdi A. Frequency and determinants of domestic violence against Iranian women during the COVID-19 pandemic: a national cross-sectional survey. *BMC Public Health* 2021; **21**: 1727.

399 Yasmin F, Jatoi HN, Abbasi MS, *et al.* Psychological Distress, Anxiety, Family Violence, Suicidality, and Wellbeing in Pakistan During the COVID-19 Lockdown: A Cross-Sectional Study. *Front Psychol* 2022; **13**: 830935.

400 Yılmaz Karaman İG, Akı Z, Çanakçı ME, Altınöz AE, Özakın E. Violence Against Women During COVID-19 Pandemic: A Comparative Study from a Turkish Emergency Department. *Prehosp Disaster Med* 2022; **37**: 462–7.

401 Yoosefi Lebni J, Irandoost SF, Xosravi T, *et al.* Explaining the problems faced by Iranian housewives during the COVID-19 quarantine period, and their adaption strategies: A qualitative study. *Women's Health* 2021; **17**: 174550652110632.

402 Yoosefi Lebni J, Enayat H, Irandoost SF, Dehghan AA. Exploring the Challenges of Afghan Refugee Women Facing COVID-19: A Qualitative Study in Iran. *Front Public Health* 2022; **10**: 838965.

403 Yoshioka T, Okubo R, Tabuchi T, Odani S, Shinozaki T, Tsugawa Y. Factors associated with serious psychological distress during the COVID-19 pandemic in Japan: a nationwide cross-sectional internet-based study. *BMJ Open* 2021; **11**: e051115.

404 Yousef AM, Sehlo MG, Mohamed AE. The negative psychological impact of COVID-19 pandemic on mothers of children with attention deficit hyperactivity disorder (ADHD): a cross-section study. *Middle East Current Psychiatry* 2021; **28**: 57.

405 Zagloul NM, Farghaly RM, ELKhatib H, Issa SY, El-Zoghby SM. Technology facilitated sexual violence: a comparative study between working and non-working females in Egypt before and during the COVID-19 pandemic. *Egypt J Forensic Sci* 2022; **12**: 21.

406 Zaigham M, Linden K, Sengpiel V, *et al.* Large gaps in the quality of healthcare experienced by Swedish mothers during the COVID-19 pandemic: A cross-sectional study based on WHO standards. *Women and Birth* 2022; **35**: 619–27.

407 Zsilavecz A, Wain H, Bruce JL, *et al.* Trauma patterns during the COVID-19 lockdown in South Africa expose vulnerability of women. *South African Medical Journal* 2020; **110**: 1110.

408 Zulaika G, Bulbarelli M, Nyothach E, *et al.* Impact of COVID-19 lockdowns on adolescent pregnancy and school dropout among secondary schoolgirls in Kenya. *BMJ Glob Health* 2022; **7**: e007666.

409 Al-Rantisi AM, Faraj OU. The consequences of government measures in Palestine to mitigate the impact of COVID-19 on gender-based violence. *Journal of Adult Protection* 2022; **24**: 215–30.

410 Boxall H, Morgan A. Who is most at risk of physical and sexual partner violence and coercive control during the COVID-19 pandemic? *Trends and Issues in Crime and Criminal Justice* 2021. https://www.scopus.com/inward/record.uri?eid=2-s2.0-85106368337&partnerID=40&md5=680faa490fd123e1907301ea4441d692.

411 Cannon CE, Ferreira R, Buttell F. A disaster's disparate impacts: analysing perceived stress and personal resilience across gender and race. *Disasters* 2022. DOI:https://dx.doi.org/10.1111/disa.12558.

412 Dey D, Tripathi P. Compromised well-being: implications on female geriatric abuse during the COVID-19 crisis in India. *Int J Hum Rights Healthc* 2022; published online Aug 19. DOI:10.1108/IJHRH-02-2022-0008.

413 Jetelina KK, Knell G, Molsberry RJ. Changes in intimate partner violence during the early stages of the COVID-19 pandemic in the USA. *Injury Prevention* 2021; **27**: 93–7.

414 Cantarero TN, Perez AS, Costa SM, Rodriguez JJ V. Television Coverage of Gender-Based Violence in Spain During Lockdown. *Violence Gend* 2022; **9**: 105–14.

415 Morgan A, Boxall H. Social isolation, time spent at home, financial stress and domestic violence during the COVID-19 pandemic. *Trends and Issues in Crime and Criminal Justice* 2020. https://www.scopus.com/inward/record.uri?eid=2-s2.0-85106349878&partnerID=40&md5=1881b3e4e638b34b60d903c277bace6c.

416 Morales-Arjona I, Pastor-Moreno G, Ruiz-Pérez I, Sordo L, Henares-Montiel J. Characterization of Cyberbullying Victimization and Perpetration Before and During the COVID-19 Pandemic in Spain. *Cyberpsychol Behav Soc Netw* 2022; **25**: 733–43.

417 Castillo JG, Moscoso B. COVID-19 Pandemic and Violence: Contagions and Curfew Policy on Female Homicides. *Violence Gend* 2022; **9**: 170–8.

418 Bardales Mendoza OT, Meza Díaz R, Carbajal M. Feminicide Violence Before and During the COVID-19 Health Emergency. *Violence Gend* 2022; **9**: 30–5.

419 Nabukeera M. Prevention and response to gender-based violence (GBV) during novel Covid-19 lock-down in Uganda. *The Journal of Adult Protection* 2021; **23**: 116–33.
